# Individual social contact data and population mobility data as early markers of SARS-CoV-2 transmission dynamics during the first wave in Germany—an analysis based on the COVIMOD study

**DOI:** 10.1186/s12916-021-02139-6

**Published:** 2021-10-14

**Authors:** Damilola Victoria Tomori, Nicole Rübsamen, Tom Berger, Stefan Scholz, Jasmin Walde, Ian Wittenberg, Berit Lange, Alexander Kuhlmann, Johannes Horn, Rafael Mikolajczyk, Veronika K. Jaeger, André Karch

**Affiliations:** 1 Institute of Epidemiology and Social Medicine, University of Münster, Münster, Germany; 2 Immunization Unit, Robert Koch Institute, Berlin, Germany; 3 Institute for Medical Epidemiology, Biostatistics and Informatics, University of Halle, Halle, Germany; 4 Department of Epidemiology, Helmholtz Centre for Infection Research, Brunswick, Germany; 5 German Center for Infection Research, Hannover-Braunschweig site, Brunswick, Germany; 6 Center for Health Economics Research Hannover (CHERH), Leibniz Universität Hannover, Hanover, Germany; 7 Biomedical Research in End-Stage and Obstructive Lung Disease Hannover (BREATH), German Center for Lung Research (DZL), Hanover, Germany; 8 Faculty of Medicine, University of Halle, Halle, Germany

**Keywords:** Contact patterns, COVID-19, Contact surveys, Pandemic

## Abstract

**Background:**

The effect of contact reduction measures on infectious disease transmission can only be assessed indirectly and with considerable delay. However, individual social contact data and population mobility data can offer near real-time proxy information. The aim of this study is to compare social contact data and population mobility data with respect to their ability to reflect transmission dynamics during the first wave of the SARS-CoV-2 pandemic in Germany.

**Methods:**

We quantified the change in social contact patterns derived from self-reported contact survey data collected by the German COVIMOD study from 04/2020 to 06/2020 (compared to the pre-pandemic period from previous studies) and estimated the percentage mean reduction over time. We compared these results as well as the percentage mean reduction in population mobility data (corrected for pre-pandemic mobility) with and without the introduction of scaling factors and specific weights for different types of contacts and mobility to the relative reduction in transmission dynamics measured by changes in *R* values provided by the German Public Health Institute.

**Results:**

We observed the largest reduction in social contacts (90%, compared to pre-pandemic data) in late April corresponding to the strictest contact reduction measures. Thereafter, the reduction in contacts dropped continuously to a minimum of 73% in late June. Relative reduction of infection dynamics derived from contact survey data underestimated the one based on reported *R* values in the time of strictest contact reduction measures but reflected it well thereafter. Relative reduction of infection dynamics derived from mobility data overestimated the one based on reported *R* values considerably throughout the study. After the introduction of a scaling factor, specific weights for different types of contacts and mobility reduced the mean absolute percentage error considerably; in all analyses, estimates based on contact data reflected measured *R* values better than those based on mobility.

**Conclusions:**

Contact survey data reflected infection dynamics better than population mobility data, indicating that both data sources cover different dimensions of infection dynamics. The use of contact type-specific weights reduced the mean absolute percentage errors to less than 1%. Measuring the changes in mobility alone is not sufficient for understanding the changes in transmission dynamics triggered by public health measures.

**Supplementary Information:**

The online version contains supplementary material available at 10.1186/s12916-021-02139-6.

## Background

The role of social contacts in the spread of respiratory infections has been discussed extensively in the year 2020 due to the global outbreak of severe acute respiratory syndrome coronavirus type 2 (SARS-CoV-2) [1, 2]. As of March 2021, over 100 million confirmed cases and over 2.5 million deaths have been recorded worldwide [2]. SARS-CoV-2 is primarily transmitted via droplets and aerosols, so person-to-person contacts are a strong determinant of transmission dynamics [2–4]. Non-pharmaceutical interventions (NPIs) focusing on the reduction of person-to-person contacts are one of the cornerstones of the pandemic response. In the middle of March 2020, Germany mandated school and kindergarten closures, postponed academic semesters, prohibited visiting of nursing homes and restricted the number of people allowed at public and private gatherings in an attempt to protect the vulnerable groups [5]. In the following weeks, contact reduction measures were implemented on a population level by regulating the maximum number of close social contacts outside one’s household and by closing non-essential shops as well as places for leisure activities [5]. After a considerable reduction in reported case numbers, federal governments decided to ease these restrictions gradually starting at the beginning of May 2020.

Social contact patterns are known to be a critical factor for the transmission dynamics of respiratory infections [4, 6–10]. However, empirical social contact data have been scarce before the emergence of SARS-CoV-2 [11–13]. One exception is the POLYMOD study, a large-scale survey that described social mixing patterns in eight European countries [12]. In 2005/2006, POLYMOD measured contacts of more than 7000 participants across eight European countries [12]. Contact patterns observed in POLYMOD have been widely used to parametrize various mathematical models of infectious disease dynamics [3, 4, 12, 14].

During the SARS-CoV-2 pandemic, contact surveys were initiated in several countries to understand the effect of contact precaution measures on social contact patterns [3, 4, 10, 15–19]. While contact surveys offer a direct approach to social contact patterns, they are time- and cost-intensive and need to be initiated actively. Mobile phone-based mobility data offer a complementary approach to infer changes in contact patterns in a population. Google and Apple granted free access to anonymized mobility data in a global attempt to provide insights into the change of mobility during the pandemic given different physical distancing policies [20, 21]. Several SARS-CoV-2 modelling studies assumed that aggregated mobility data can be used as a proxy for the actual number and intensity of contacts of individuals in a defined population, although mobility data measure only certain dimensions of contact behaviour. In this article, we present survey-based social contact data for the first wave of the pandemic in Germany and assess their ability to reflect transmission dynamics 10 days later (measured by reported reproduction number (*R* estimates)) when compared to open source population mobility data from Google and Apple [20–22].

## Methods

### Contact surveys

#### Pandemic contact survey—COVIMOD

The contact survey COVIMOD was initiated in April 2020 based on participants of the online panel i-say.com. To ensure the samples’ broad representativeness of the German population, participants were recruited by sending email invitations to existing members of the panel based on age, sex and regional quotas. To gain information on children’s social contacts, a defined subgroup of adult participants with under-aged children (< 18 years of age) living in their household were invited to provide information as a proxy for their children. This approach, however, resulted in the sample being no longer representative of the German population as we under-sampled the middle-aged participants who instead filled out the questionnaire for their children. The first COVIMOD survey wave was launched on 30/04/2020 corresponding to the time of the strictest contact reduction measures in Germany. Survey waves 2 to 4 were launched during a time of a gradual easing of the contact reduction measures in May and June 2020. For wave 1, a sample of 1500 participants was recruited with an expected response rate of 85% for the next survey waves. Before the launch of survey wave 4, the sample was increased by 1000 additional participants.

The COVIMOD questionnaire is based on the questionnaire of the CoMix study and includes questions on demographics, current behaviours, attitudes towards SARS-CoV-2 and the social contacts of participants [3]. Participants were asked to provide each social contact between 5 am the preceding day and 5 am the day of the survey, the age and sex of the contact, the duration they spend with each contact, the setting where the contact occurred and if the contact was a household member or not. The questionnaire can be found in Additional file 1.

We defined a contact in COVIMOD in line with the POLYMOD study’s definition as “people who you met in person and with whom you exchanged at least a few words, or with whom you had physical contact” [12]. During survey waves 1 and 2, participants were asked to provide each contact separately. Instead of providing each contact one by one, some participants included a group of contacts as one contact (e.g. “customers”). For these groups, we assumed a specific number of social contacts (Additional file 2). From survey wave 3 onwards, participants were offered the opportunity to provide a number of additional contacts (group contacts) they were not able to list individually in case they had too many contacts.

As participants were offered to enter these additional contacts separately, we used different analysis approaches to work with these contacts (sensitivity analyses). The main scenario includes all reported contacts plus group contacts weighted for the German population for COVIMOD and POLYMOD. Unweighted results and those without group contacts can be found in Additional file 3.

#### Pre-pandemic contact survey—POLYMOD

The European contact survey POLYMOD was used as a baseline pre-pandemic comparison. In Germany, POLYMOD was conducted paper-based with the help of a market research company in 2005/2006. Further details about POLYMOD can be found elsewhere [12]. As in COVIMOD, participants in POLYMOD were also allowed to enter the number of additional contacts (group contacts) they had if participants had too many contacts to report them separately.

### Mobility data

We obtained publicly available aggregated mobility data from the Google COVID-19 Community Mobility Reports and from the COVID-19 Apple Mobility trends for the times corresponding to the COVIMOD survey waves [20, 21].

Google COVID-19 Community Mobility Reports provide the percentage change in mobility from February 2020 onwards compared to the median of the corresponding weekday between 03/01/2020 and 06/02/2020. Google COVID-19 Community Mobility Reports use aggregated information about true individual movement histories to provide location-specific changes in mobility over time. Data are stratified by the destination of the movement, i.e. retail and recreation, grocery and pharmacy, transit stations, workplace, residential and parks. COVID-19 Apple Mobility trends provide information about the relative volume of requests for directions for all weeks in 2020 compared to a base volume on 13/01/2020.

### Reproduction number estimates by the German Public Health Institute

*R* values used in our analysis as the “reference standard” for infection dynamics were obtained from the German Public Health Institute (Robert Koch Institute (RKI)) [22, 23]. The method applied by the RKI to obtain current *R* values is based on the reported numbers of individuals notified for being newly infected with SARS-CoV-2 and includes a nowcasting approach taking into account the delay in diagnosis, reporting and data delivery. If possible, incident cases are attributed to the day of first symptoms (an information available for the majority of cases in the German notification system). If this information is not available, it is imputed taking into account measured delays from the day of the first symptom to the notification date, age of the case and day and week of notification. Based on this nowcasting, RKI estimates the time-dependent reproduction number [24]. The 4-day reproduction number calculated by the RKI provides information on the transmission dynamics 8 to 13 days prior [23]. The *R* values are continuously corrected retrospectively for delayed notifications. We used *R* values provided by the RKI for 10 days after the timing of our survey waves as a reference for the comparison of infection dynamics. Since we extracted *R* values more than 1 year after the day they were calculated for, all delayed notifications were already accounted for. The *R* values based on case numbers as reported by RKI reflect both changes in transmission dynamics due to contact reduction measures as well as due to developing immunity in the population, while contact survey and mobility data cannot take into account population immunity. For this analysis, we assumed that SARS-CoV-2 immunity in the population is negligible for our analyses as this study only includes the first wave of the SARS-CoV-2 pandemic in Germany, and seroprevalence estimates for this period are below 1% in representative studies [25].

### Data management and statistical analyses

#### Contact surveys

As the COVIMOD sample is not fully representative of the German population, we used data from the 2011 census to apply survey weights based on the participants’ age, sex, household size and region of residence [26]. The region of residence was not available for POLYMOD, so the POLYMOD data were only weighted according to the participants’ age, sex and household size using the R package “survey” [27]. As the COVIMOD data collection was not always started on the same day of the week and the duration of the survey waves did vary slightly, we also weighted both COVIMOD and POLYMOD for weekdays/weekends.

We calculated the mean number of social contacts per participant per day as well as the 95% confidence interval of the bootstrapped mean of 1000 samples. We stratified social contacts by age group, sex, household size and the day of the week. Additionally, we assessed setting-specific contacts, i.e. home, childcare/school/university, work, public transport and others; childcare/school/university contacts were assessed in the subgroup of participants who reported to attend childcare, school or university, and work contacts were assessed in the subgroup of participants who worked full- or part-time. We calculated social contact matrices for the age-specific mean number of direct social contacts using the “socialmixr” package in R [28]. To obtain the final contact matrices, the age-specific mean number of daily contacts were adjusted, so that the total number of contacts of one group with another was the same as vice versa [28]. For the calculation of the contact matrices, participants who reported more than 100 group contacts were excluded from the analysis (COVIMOD: wave 3, 6 participants; wave 4, 13 participants; POLYMOD, 10 participants).

To assess how changes in infection dynamics are reflected by contact survey data, we applied two different approaches. First, we performed a simple analysis for which we calculated the mean relative reduction in contacts for each COVIMOD wave when compared to pre-pandemic data. For this, we translated the number of the mean contacts and the corresponding 95% confidence interval values into a mean relative reduction from baseline, i.e. in this case, the number of mean contacts before the SARS-CoV-2 pandemic as estimated in the POLYMOD study.

Second, we performed a more complex analysis by using additional information from the contact survey for calculating the next-generation matrix. We assumed that the next-generation matrix for SARS-CoV-2 is a function of the age-specific effective contact rate, given by the number of age-specific contacts multiplied by the probability of transmission per contact, and the duration of infectiousness [29]. Hence, the basic reproduction number (R0) is proportional to the dominant eigenvalue of the contact matrix [30]. To be able to calculate *R* as the result of a relative reduction in R0, we assumed that the social contact patterns before the implementation of the contact reduction measures were similar to the POLYMOD contact patterns and that the duration of infectiousness and the per-contact transmission probability remained constant. Additionally, we assumed that the transmission probability did not depend on age. Under these assumptions, the relative reduction of *R* compared to R0 is equivalent to the reduction in the contact matrices’ dominant eigenvalue allowing us to estimate the reproduction number corresponding to contacts recorded in COVIMOD. We assumed R0 during the first wave in Germany to follow a normal distribution with a mean of 2.6 and a standard deviation of 0.54 [3]. We drew 10,000 bootstrap samples from POLYMOD and COVIMOD to assess uncertainty.

Similar to the first approach, we then translated the *R* estimates from the COVIMOD study into a mean relative reduction from baseline, i.e. in this case, the basic reproduction number (assumed as R0 = 2.6).

#### Mobility data

We used mobility data collected for the same time intervals as the COVIMOD waves’ timings and compared it to the pre-pandemic data available from the respective data sources. In addition to assessing the distinct movement types provided by Google, we also composed an indicator for overall mobility by averaging across all the movement types separately for both the Google mobility data and the Apple mobility data (with the exception of movements to parks as this is expected to vary considerably during seasons).

We calculated the mean relative change compared to pre-pandemic data within the time intervals corresponding to the COVIMOD waves as well as the 95% confidence interval of the bootstrapped mean of 1000 samples for Google and Apple mobility. In line with the approach we applied for COVIMOD and POLYMOD, we weighted the population mobility data for weekdays/weekends.

#### RKI reproduction number estimates

We calculated the mean *R* estimates for the corresponding time intervals 10 days after the COVIMOD waves as well as 95% confidence interval of the bootstrapped mean of 1000 samples based on the daily *R* estimates provided by the RKI, the German Public Health Institute. We then translated the mean and 95% confidence interval value into a relative reduction from baseline, i.e. in this case, the basic reproduction number (assumed as R0 = 2.6 during the first wave in Germany), to provide a reference standard for infection dynamics against which the changes in social contact data and population mobility data could be compared.

#### Weights by contact type and calibration of scaling factors

As the probability that a contact leads to a transmission varies according to the setting, we performed additional analyses using two different concepts to take this into account. First, we assigned different but specific weights to home contacts/home mobility and non-home contacts/non-home mobility (i.e. all other contact settings combined) based on setting-specific secondary attack rates (SAR) from a systematic review by Thompson et al. [31]. Based on Thompson et al., the household SAR was estimated to be 21.1 and the SAR in a healthcare setting, at the workplace and with casual close contacts to be 3.6%, 1.9% and 1.2%, respectively. We used normalised weights based on household SAR and the average of the healthcare, workplace and casual close contacts (SAR = 2.23%) and applied the household weight to the home contacts/home mobility and the non-household weight to the non-home contacts/non-home mobility. We then allowed for an additional scaling factor per contact survey approach, i.e. simple approach—mean relative reduction in contacts, complex approach—contact data with next-generation matrix, google mobility data; the same scaling factor was used within each approach for all waves as well as for all types of contacts in the contact survey approaches and all types of mobility, in the mobility approach. We used this scaling approach with the aim to obtain the minimum residual sum of squares across the four survey waves when compared to our reference standard, i.e. relative reductions estimated based on *R* values reported by the RKI. For a better understanding of the effect of contact/mobility-type weights, we also performed an analysis in which we fitted the scaling factor with the same weight for all types of contacts and mobility. In the second concept, we did not apply pre-defined weights for home/non-home contacts and for home/non-home mobility but fitted them from the data by allowing independent scaling factors for home contacts and home mobility and non-home contacts and non-home mobility per approach, i.e. simple approach—mean relative reduction in contacts, complex approach—contact data with next-generation matrix, google mobility data. By doing so, we estimated the relative weights for both contact/mobility types based on the data collected for this study and did not take into account external information for transmission probabilities in different settings. The optim function in R was used for the fitting/scaling. Apple mobility data could not be used for these analyses as there is no differentiation in home/non-home mobility available.

#### Comparison of the results of the different approaches with the reference standard

For all analyses, we calculated the mean absolute percentage error of the estimates obtained by the approaches for the COVIMOD contact data as well as for the Google and Apple mobility data when compared to the reference standard of relative changes in infection dynamics based on *R* estimates from RKI. We did this in the base case concept without scaling factor and contact type-specific weighting, as well in all three concepts with scaling factors. Moreover, we applied repeated measures ANOVA to assess the differences between error rates provided by the different data sources.

R version 4.0.2 was used for all analyses [32]. Further specifications of the analyses can be found in Additional file 4.

## Results

### Participant characteristics of POLYMOD and COVIMOD

During POLYMOD, 1341 participants were surveyed in Germany; they recorded a total of 27,154 contacts. In the first COVIMOD wave, we surveyed 1560 participants who recorded a total of 3256 social contacts; this changed to 1356 participants with a total of 4852 contacts in the second survey wave, 1081 participants with a total of 6344 in the third wave and 1890 participants with a total of 13,471 contacts in the fourth wave.

The youngest participants in all COVIMOD waves were younger than 1 (the parents were surveyed as a proxy), and the oldest was 91 years of age. Between 47% and 50% of all COVIMOD participants were female (Table 1). In POLYMOD and all COVIMOD waves, the median household size of the participants was 3 (POLYMOD IQR 2–4, COVIMOD wave 1 IQR 2–4, wave 2 IQR 2–3, wave 3 IQR 2–3, wave 4 IQR 1–3). In COVIMOD survey waves 1, 2 and 3, most participants reported their social contacts on a Thursday, whereas in wave 4, most contacts were reported on a Monday; less than a quarter of participants reported the contacts during the weekend (Table 1).

**Table 1:** Participant characteristics in the COVIMOD survey waves one to four compared to the POLYMOD survey

	POLYMOD		COVIMOD							
			Wave 1		Wave 2		Wave 3		Wave 4	
			30/04 to 06/05/2020		14/05 to 21/05/2020		28/05 to 04/06/2020		11/06 to 22/06/2020	
	***N***	Percent	***N***	Percent	***N***	Percent	***N***	Percent	***N***	Percent
	1341		1560		1356		1081		1890	
**Age category**										
0–4	89	6.9	46	2.9	36	2.7	21	1.9	56	3.0
5–9	92	7.1	48	3.1	41	3.0	30	2.8	62	3.3
10–14	110	8.5	73	4.7	63	4.7	45	4.2	87	4.6
15–19	121	9.3	95	6.1	66	4.9	45	4.2	108	5.7
20–24	117	9.0	83	5.3	60	4.4	28	2.6	109	5.8
25–34	132	10.2	173	11.1	148	10.9	96	8.9	219	11.6
35–44	156	12.0	137	8.8	124	9.2	91	8.4	164	8.7
45–54	184	14.2	235	15.1	209	15.4	174	16.1	275	14.6
55–64	160	12.4	265	17.0	244	18.0	237	21.9	321	17.0
65–69	74	5.7	270	17.3	245	18.1	199	18.4	313	16.6
70–74	33	2.5	89	5.7	73	5.4	79	7.3	118	6.2
75–79	14	1.1	35	2.2	34	2.5	29	2.7	48	2.5
80+	13	1.0	11	0.7	11	0.8	7	0.6	10	0.5
Missing	46	–	0	–	2	–	0	–	0	–
**Sex of participants**										
Female	722	55.4	748	48.1	638	47.1	536	49.6	901	47.8
Male	581	44.6	806	51.9	717	52.9	544	50.4	985	52.2
Missing	38	–	6	–	1	–	1	–	4	–
**Household size**										
1	250	18.6	232	14.9	256	18.9	268	24.8	487	25.8
2	411	30.6	412	26.4	351	25.9	270	25.0	439	23.2
3	339	25.3	514	32.9	447	33.0	343	31.7	544	28.8
4 or more	341	25.4	402	25.8	302	22.3	200	18.5	420	22.2
**Weekday for which contacts were reported**										
Monday	227	17.2	50	3.2	60	4.4	128	11.8	642	34.0
Tuesday	237	18.0	63	4.0	89	6.6	143	13.2	246	13.0
Wednesday	222	16.8	54	3.5	293	21.6	87	8.0	172	9.1
Thursday	179	13.6	914	58.6	613	45.2	489	45.2	320	16.9
Friday	186	14.1	144	9.2	117	8.6	132	12.2	196	10.4
Saturday	152	11.5	88	5.6	63	4.6	66	6.1	81	4.3
Sunday	117	8.9	247	15.8	121	8.9	36	3.3	233	12.3
Missing	21	–	0	–	0	–	0	–	0	–

Missing in COVIMOD included participants who preferred not to answer the question

A comparison of the characteristics of the German population and the POLYMOD and COVIMOD participants can be found in Additional file 3, Table 1. Participant characteristics after weighting can be found in Additional file 3, Table 1.1a. The analyses hereafter are based on the weighted data including group contacts.

### Number of social contacts

**Fig. 1:**
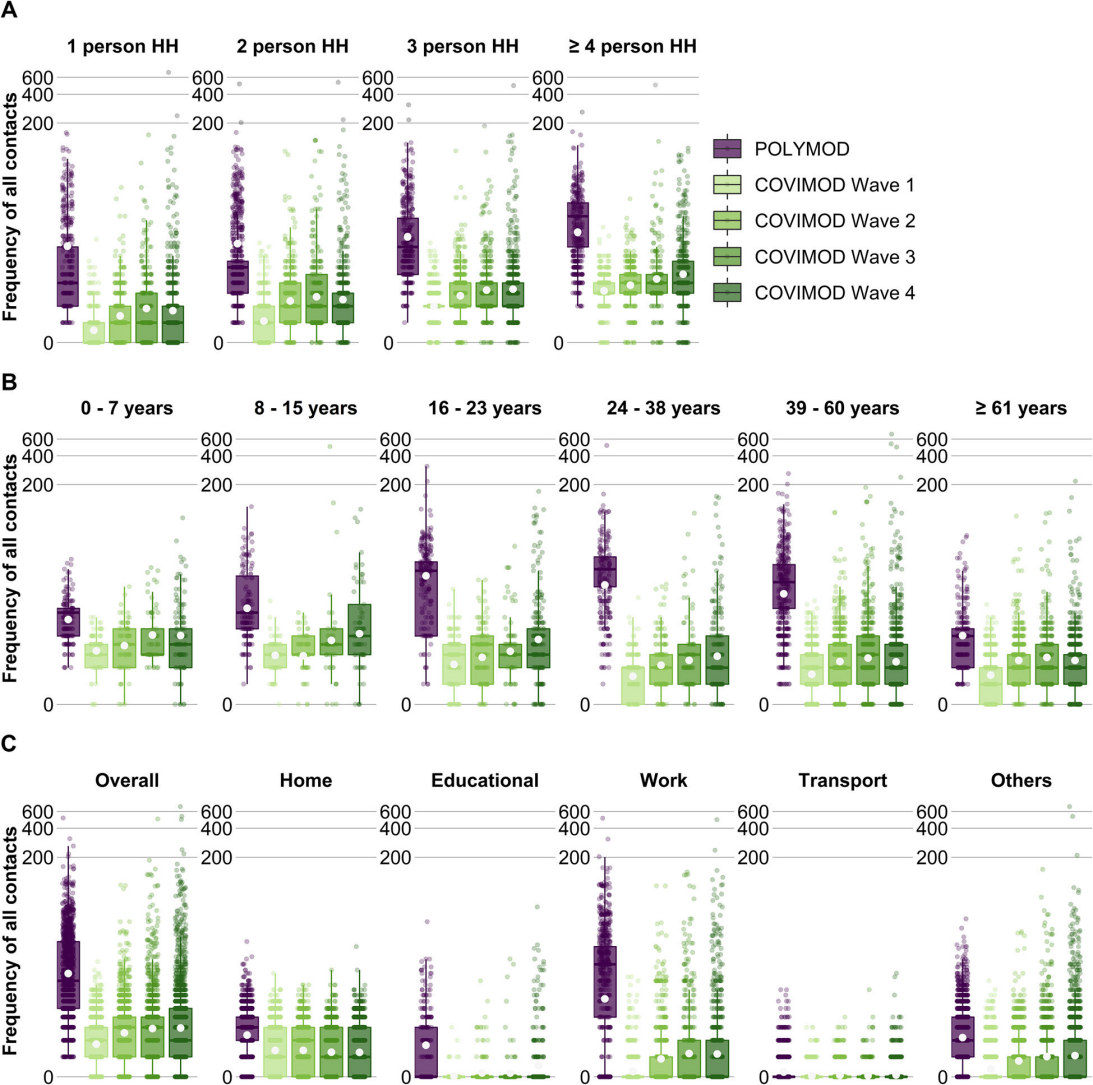
Number of all contacts during the POLYMOD and COVIMOD surveys. Displayed are the number of contacts **A** stratified by household size, **B** stratified by age and **C** according to the settings in which the contact took place. Boxes represent the 25th, 50th and 75th percentiles, the whiskers represent the 10th and 90th percentile and the white dots represent the mean. Note: the displayed educational contacts are based only on the group of participants who attended an educational facility (kindergarten, school, university), and work contacts are based only on the group of participants who reported to work full-/part-time. Participants with no contacts are displayed as 0 on the log-scale of the *y*-axis

The mean number of contacts measured per participant during all COVIMOD waves (wave 1, 2.0 contacts (SD 1.9); wave 2, 3.3 contacts (SD 4.7); wave 3, 6.2 contacts (SD 18.4); wave 4, 6.9 contacts (SD 326.3)) was considerably lower in comparison with the 18.9 contacts (SD 24.6) measured in POLYMOD in the pre-pandemic period (Fig. 1C; Additional file 3 Table 1.2a). The reduction in the number of overall contacts between POLYMOD and COVIMOD was consistent across age, sex, household size and weekday (Fig. 1; Additional file 3 Table 1.2a).

While the mean number of home contacts was stable across all COVIMOD waves (and just a little bit lower than in POLYMOD, Fig. 1, Additional file 3 Table 1.2b), contacts at work and in educational settings were dramatically reduced during the first COVIMOD wave. Contacts at work increased gradually thereafter but remained much lower than in POLYMOD even for survey wave 4; educational contacts started to increase only at survey wave 4 as schools were closed before (Fig. 1, Additional file 3 Table 1.2b). Moreover, the distribution of contacts observed changed considerably over the different COVIMOD waves. While the maximum number of contacts reported overall and in specific settings was clearly reduced in the first COVIMOD wave, it approximated the one reported in POLYMOD already in waves 2 and 3 and reached it in wave 4 (although the mean and median contact numbers were still clearly reduced). The number of contacts in different settings for the other analyses can be found in Additional file 3 Tables 2.2b, 3.2b and 4.2b.

POLYMOD and COVIMOD participants in all age groups shared the majority of their contacts with individuals of similar age, demonstrating the expected age-assortative pattern (Additional file 3 Figure 1.5; Additional file 5). Contact matrices derived from the first two COVIMOD waves were dominated by contacts at home, revealing mainly contacts with life partners and children. This changed slowly through survey waves 3 and 4 due to the gradual increase in work and leisure time (“other”) contacts, which resulted in a broader distribution of the age of potential contact persons (Additional file 3 Figure 1.5).

### Representation of transmission dynamics by contact survey and mobility data

**Fig. 2:**
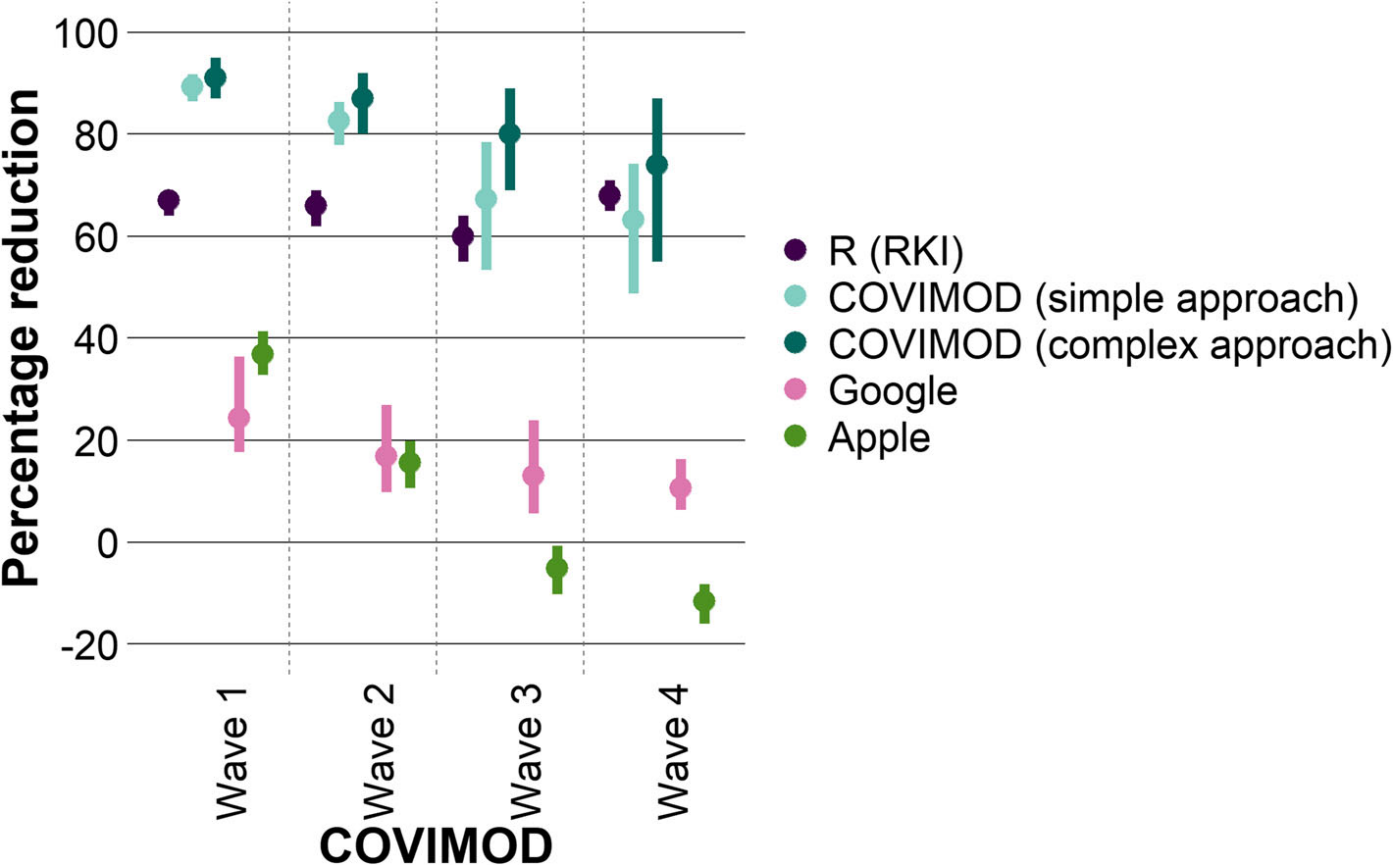
Comparison of the relative reduction in transmission dynamics based on different input data. Displayed are the mean and bootstrapped 95% confidence interval of the relative reduction of the *R* estimates from the RKI compared to the basic reproduction number, the relative reduction in the number of social contacts of the COVIMOD study for the simple and the more complex approach compared to the contacts before the SARS-CoV-2 pandemic as well as of the mobility data (Google and Apple) compared to the mobility before the SARS-CoV-2 pandemic

**Fig. 3:**
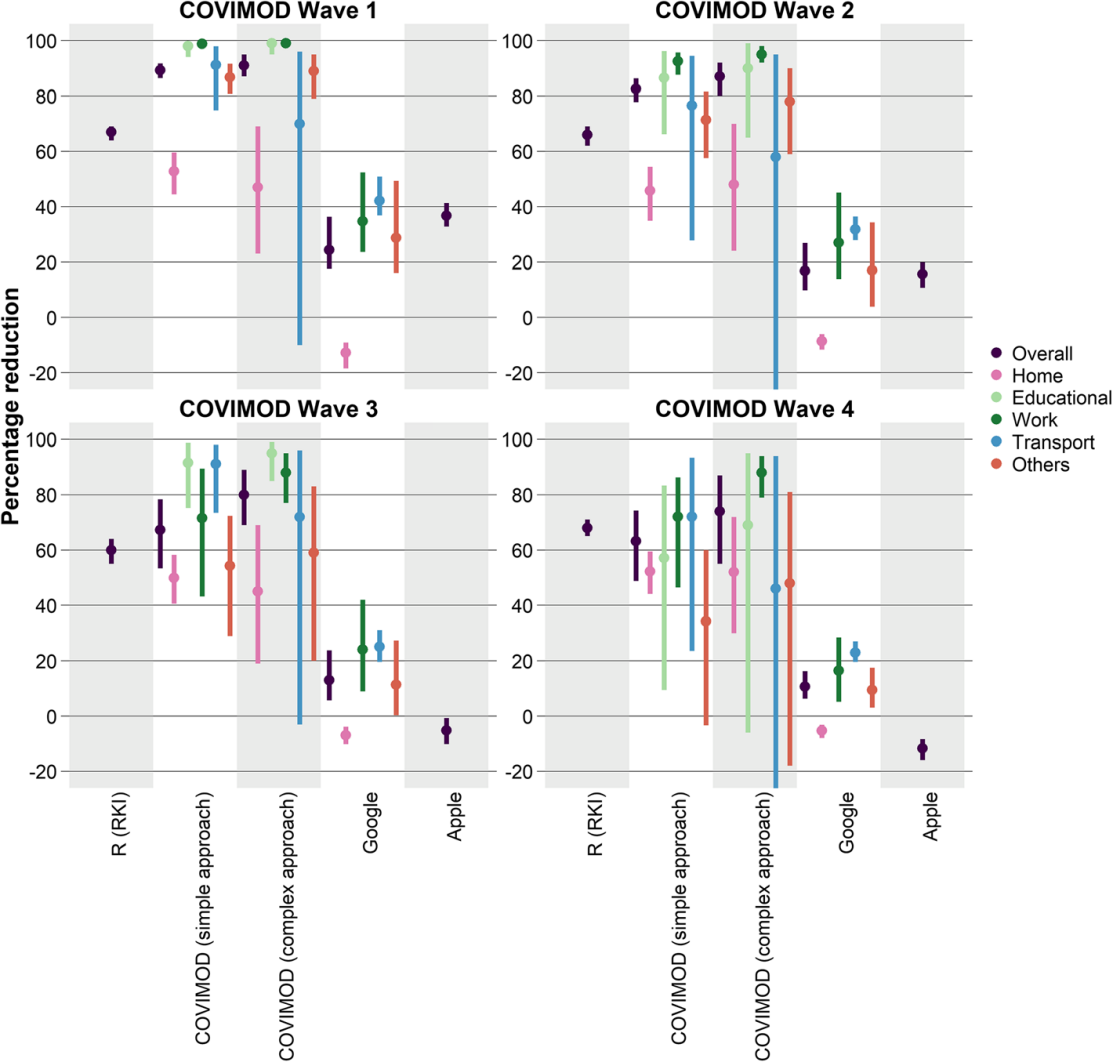
Comparison of the relative reduction in transmission dynamics by settings based on different input data. Displayed are the mean and bootstrapped 95% confidence interval of the percentage reduction of the *R* estimates from the RKI and *R* estimates obtained from COVIMOD (the complex approach) compared to the basic reproduction number, the percentage reduction in the number of social contacts of the COVIMOD study (simple approach) compared to pre-pandemic times and the percentage reduction of the Google and Apple mobility data

In the base case approach without scaling factors and contact type-specific weighting, the mean *R* estimated based on the next-generation matrices of COVIMOD data was smaller than 1 in all COVIMOD waves (representing a mean relative reduction in contacts of at least 75%); we observed the highest mean relative reduction with 91% at the end of April (survey wave 1), which corresponds to the time of the strictest contact reduction measures. Subsequently, the mean relative reduction decreased with time as the contact reduction measures were loosened (wave 2, 87%; wave 3, 80%; wave 4, 74%; Figs. 2 and 3; Additional file 3 Table 1.3b).

A very similar pattern both in the lowering of the mean relative reduction and in the level of the relative reduction was seen with the simple approach based only on the reduction of the number of contacts itself. We observed a mean relative reduction between 89% at the end of April and 63% in the middle of June 2020 (survey wave 4; Figs. 2 and 3).

Compared to the relative reductions estimated based on *R* values reported by the RKI, relative reductions in contacts measured by COVIMOD both in the simple and the more complex approach were higher during the first survey wave but fit quite well during waves 2 to 4. Mobility reduction estimates based on Google and Apple data were considerably smaller than relative reductions estimated based on *R* values reported by the RKI throughout the entire study (Figs. 2 and 3; Additional file 3 Table 1.3a and b). Both mobility data sources found mobility patterns similar to pre-pandemic data already during the time of survey waves 1 and 2, while reported *R* values 10 days later were still considerably below 1.

**Fig. 4:**
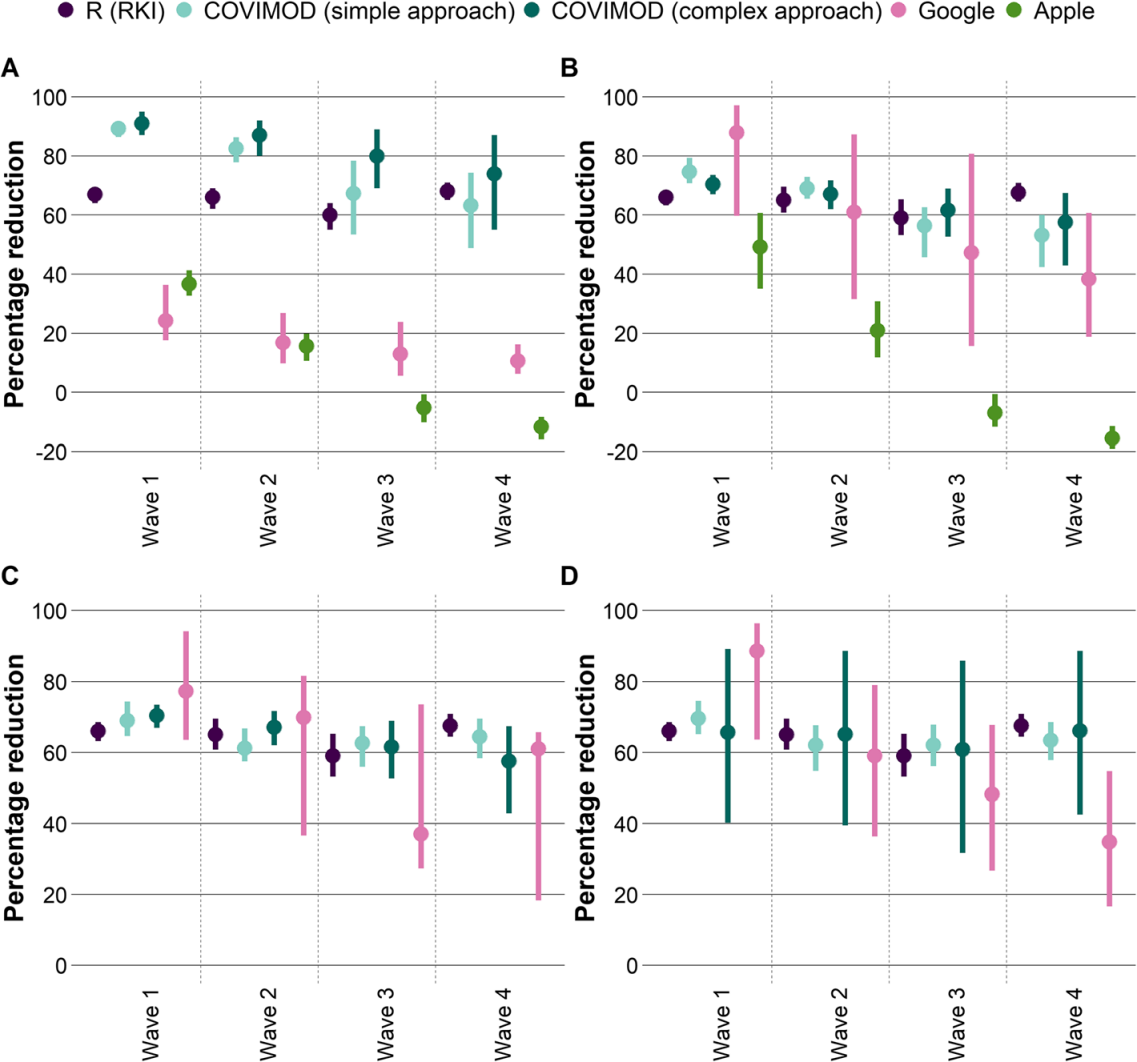
Comparison of the estimates of the relative reduction in transmission dynamics by scaling/weighting approach. In each figure, the mean and bootstrapped 95% confidence interval of the percentage reduction of the reference standard, i.e. the *R* estimates from the RKI compared to the basic reproduction number is displayed; the mean and bootstrapped 95% confidence interval of the percentage reduction in the social contact data of the COVIMOD study for the simple and the more complex approach are displayed as well as the mean and bootstrapped 95% confidence interval of the percentage reduction in the mobility data (Google and Apple) compared to the mobility before the SARS-CoV-2 pandemic. Displayed is the base case approach without scaling/weighting applied (**A**) and with a scaling factor but without separate weighing for home/non-home contacts (**B**). (**C**) shows the estimates relative reduction in transmission dynamics with fitted weights for home/non-home contacts/mobility and (**D**) with normalised weights for home/non-home contacts/mobility by [31] as well as allowing a scaling factor

The mean absolute percentage error of the relative reduction measured in COVIMOD based only on the reduction of contacts itself (the simple approach) was 19% (SD 12), measured in COVIMOD based on the more complex derivation of the next-generation matrix was 28% (SD 12), measured based on Google mobility data was 75% (SD 8) and measured based on Apple mobility data was 87% (SD 33). The mean absolute percentage error (MAPE) of the simple and more complex COVIMOD approach were smaller than the ones obtained via Google (*p* < 0.001 for both approaches) and Apple mobility data (*p* = 0.010 and *p* = 0.015). The introduction of a scaling factor reduced MAPE values considerably, especially for both COVIMOD approaches (Fig. 4).

According to the systematic review of Thompson et al. [31], the SAR for the healthcare/workplace/casual close contacts is around 10% of that of household contacts. When we fitted the reduction in social contacts based on the simple approach to the relative reductions estimated based on *R* values reported by the RKI, the best fit for the relative transmission risk for non-home contacts compared with home contacts was obtained with a very similar estimate of around 8% compared to around 20% in the mobility data. The mean absolute percentage error decreased to 5% (SD 0.7%) based on COVIMOD and to 18% (SD 14%) based on Google when we used this approach to derive contact/mobility type-specific weights and scaling factors. Applying the estimates from Thompson et al. [31], the mean absolute percentage error was very similar for COVIMOD (5%, SD 0.25%) but larger for estimates based on Google mobility (27%, SD 17%). An even lower mean absolute percentage error was obtained by using the more complex contact survey data approach based on the next-generation matrix (mean absolute percentage error of 1% (SD 1%) for both weighting based on estimates by Thompson et al. [31] and fitting of home/non-home contacts (Fig. 4).

## Discussion

In this study, we quantified the relative reduction in contacts based on contact survey data and publicly available mobility data. We found that both data sources represent different dimensions of transmission dynamics; changes in contact patterns measured in survey data represented transmission dynamics (measured as *R*) better than the changes measured in aggregated mobility data independently of the introduction of contact- and mobility type-specific weights and the use of scaling factors. Non-pharmaceutical interventions introduced in Germany during the first wave of the SARS-CoV-2 pandemic were, however, associated with both a considerable reduction in social contacts reported in contact surveys as well as with reductions in mobility patterns. The results of our study indicate that deriving contact behaviour from mobility data alone, as it was often the case in political decision-making during the first and second wave, is not suitable for making real-time inferences on the effects of public health measures on the transmission dynamics in a population. Mobility data used in this study suggested that contact behaviour went back to normal almost instantly after the contact reduction measures were relaxed, which did not reflect the observed *R* values. A reason for that might be that people still tried to minimise close contacts outside their own households and maximised distance to the contacts they had, although their mobility, e.g. back to work, already reached almost pre-pandemic levels. Therefore, a complementary approach including both aspects, i.e. social contact behaviour as well as mobility behaviour, is necessary to fully reflect transmissions dynamics. Although repeated contact surveys need considerable investment in terms of time and costs, the potential benefits and financial savings if used as a near real-time proxy for transmission dynamics on a population level are likely to outweigh the efforts needed. Benefits include a better preparedness towards expected case numbers as well as earlier information on the effect of newly introduced contact reduction measures, which allows timely adaptation if needed.

In our study, we found a 73% mean reduction in contacts across the first four waves of COVIMOD (i.e. from April to June 2020) which is consistent with studies from other European countries [3, 4]. Even though the reported number of daily contacts increased over the survey waves, it was still considerably lower than in POLYMOD, indicating sustainable behaviour change even after the end of the strictest contact reduction measures. We found an increased variance in the reported daily number of contacts as the COVIMOD waves progressed, with the maximum number of contacts increasing from 16 in survey wave 1 to 674 in wave 4, while median contact numbers were not affected similarly. Since SARS-CoV-2 has been shown to be associated with a high variance in the number of transmissions arising from one infectious individual [33], this sharp increase in the maximum number of contacts has huge implications for the risk of superspreading events as the direct aftermath of the end of public health interventions. Participants aged 60 and above reported fewer contacts in all COVIMOD waves as well as a larger reduction to pre-pandemic values when compared to children and middle-aged persons. This should be taken into account when assessing the effects of vaccination prioritisation strategies in combination with NPIs, as people in this age group are known to be more vulnerable to SARS-CoV-2 infections [15].

We further observed a smaller and more stable reduction in home contacts than in work, educational and leisure time contacts, which confirms that reduction in contacts is location-specific [3]. This is reasonable as most of the social distancing measures implemented at that time had their main impact outside the household. We confirmed that the majority of remaining contacts under strict contact reduction measures happens between life partners and parents and children, which mirrors the huge role of this transmission setting under contact reduction measures [7, 34].

When introducing contact- and mobility type-specific weights representing different transmission probabilities for home and non-home contacts/mobility, we were able to considerably reduce the differences in estimates for transmission dynamics when compared to the reported *R* values 10 days later, even if scaling factors had been fitted to the different data source models before. However, the remaining differences were in all analyses much smaller for estimates based on contact survey data than for mobility data. These results show that the presented approach might be suitable for a near real-time estimation of transmission dynamics based on contact survey data alone or in combination with mobility data. A data-driven estimation (based on contact survey data) of the relative transmission risk at home compared to non-home transmission resulted in estimates very similar to those derived from setting-specific secondary attack rates reported in the literature. Our results indicate that the differentiation in home and non-home contacts based on contact survey data supports the representation of the true role of different types of contacts for transmission dynamics.

Our analyses suggest that the use of contact survey data, especially after weighing for home and non-home contacts together with an additional scaling factor, can indeed be used as an early marker of current transmission dynamics, especially if they are mainly determined by contact reduction measures. We show that aggregated mobility data offer a different behavioural perspective but can also contribute to a better understanding of how transmission dynamics might develop in near real-time. The analyses performed in this study were rather simplistic by nature, as they aimed to provide an overall estimate of transmission dynamics without differentiating by too many different factors and without a formal dynamic mathematical model. In reality, the information provided about changes over time in contact settings, intensities and frequencies with contact partners offers especially for contact survey data but also for mobility data much wider perspectives. Since these analyses require a dynamic modelling approach taking into account various other assumptions not necessarily available in the early phases of an epidemic, they might not be as suitable for near real-time communication with decision-makers as the simpler approaches presented here. However, future analyses should focus on using the available contact and mobility data to construct and validate multi-layer mathematical models which take into account mobility data for large scale movements and contact survey data for small scale effect contacts, and this combines the strengths of the different data sources.

Our study has several limitations. COVIMOD data are not fully representative of the German population since some adult participants with under-aged children living in their households were invited to provide information as a proxy for their children. Moreover, the elderly (> 70 years) and the very young (< 10 years of age) are underrepresented in COVIMOD. We tried to correct for that by introducing weights for sex, age and household size; however, there were no relevant differences in the results of the unweighted and weighted analyses. Participants in COVIMOD were asked to record their contacts retrospectively so that different forms of information bias could have been introduced. For example, it might be challenging to remember a higher number of contacts, or the participants’ willingness to report high numbers of contacts individually might be lower as this is quite tedious and time-consuming. We tried to minimise this by allowing the participants to record group contacts. We also cannot rule out that COVIMOD attracted specifically participants who adhered to social distancing rules as these individuals might be more likely to respond to health surveys. This could have led to an overestimation of the relative reduction of contacts and could explain the gap between relative reductions in social contacts and reported *R* values. We tried to minimise this bias by using an established online panel not focusing on healthcare questions as the platform for COVIMOD. Even though contact-related questions were similarly phrased between POLYMOD and COVIMOD, POLYMOD was paper-based, and COVIMOD surveys were web-based. Previous research suggested that participants might report more contacts in paper-based surveys than in web-based surveys [11, 35]. Future research will be conducted on the differences between web- and paper-based contacts during the pandemic. However, our findings are consistent with other studies that examined social contact patterns under strict contact reduction measures [3, 4, 15, 36]. We used aggregated mobility data in our study that were freely available and have been discussed as a potential real-time proxy for SARS-CoV-2 transmission dynamics. Although we took advantage of two different data sources representing complementary ways to define mobility, our results cannot be automatically generalised to other ways of measuring mobility (e.g. based on individual movement patterns). The *R* values derived from RKI represent the changes in transmission dynamics based on contact reduction measures as well as population immunity, while contact survey data and mobility data can only assess the former. Since population immunity was below 1% in the study period, this is unlikely to have played a major role in this analysis but needs to be taken into account for future studies. Application of scaling factors, which include information on developing population immunity, might be a useful tool for later phases of an epidemic.

## Conclusions

In summary, our study provides a comprehensive quantification of social contacts and mixing patterns as well as aggregated mobility information relevant to the spread of SARS-CoV-2 during spring and summer 2020 in Germany. Our results indicate that population-based contact surveys provide a suitable platform for near real-time assessment of transmission dynamics for respiratory infections in a population in the absence of population immunity. Aggregated mobility data as a proxy for effective contacts did not show the same degree of persistent reduction. The introduction of contact and mobility type-specific weights led to a considerable improvement in the reflection of reported changes in case numbers 10 days later. Mobility data and social contact data provide information on different dimensions of human behaviour. A complementary approach including both aspects, social contact behaviour and mobility behaviour might be needed to reflect transmission dynamics best.

## Supplementary Information

**Additional file 1.** COVIMOD questionnaire. This additional file includes the questionnaire.

**Additional file 2.** Consideration of additional contacts. This file illustrates how additional contacts were dealt with in the data management process.

**Additional file 3.** Additional results. This file provides results additional to the ones provided in the manuscript.

**Additional file 4.** Additional data analyses information. This file provides more data analyses details.

**Additional file 5.** Contact matrices values. This file provides the values of the contact matrices displayed in the additional file 3.

## Data Availability

The data are available from the corresponding author upon valid scientific request.

## Abbreviations

COVID-19: Coronavirus disease 2019
NPIs: Non-pharmaceutical interventions
R0: Basic reproduction number
*R*: Reproduction number
RKI: Robert Koch Institute
SARS-CoV-2: Severe acute respiratory syndrome coronavirus 2
