# Supplementary material for: Individual social contact data and population mobility data as early markers of SARS-CoV-2 transmission dynamics during the first wave in Germany—an analysis based on the COVIMOD study
Source: BMC Med. 2021 Oct 14;19:271. doi: 10.1186/s12916-021-02139-6 (PMC8515158; doi:10.1186/s12916-021-02139-6)
Supplement: Supplementary file 2 — Additional file 2. Consideration of additional contacts. This file illustrates how additional contacts were dealt with in the data management process. [file 12916_2021_2139_MOESM2_ESM.pdf]

Individual social contact data and population mobility data as early markers of SARS-CoV-2 transmission dynamics during the first wave in Germany – an analysis based on the COVIMOD study

Additional File 2: How additional contacts were considered in COVIMOD

Damilola Victoria Tomori [1], Nicole Rübsamen [1], Tom Berger [1], Stefan Scholz [2], Jasmin Walde [1], Ian Wittenberg [3], Berit Lange [4,5], Alexander Kuhlmann[6,7], Johannes Horn[3], Rafael Mikolajczyk [3], Veronika K Jaeger [1*], André Karch [1*]

[1]Institute of Epidemiology and Social Medicine, University of Münster, Münster, Germany

[2]Immunization Unit, Robert Koch-Institute, Berlin, Germany

[3]Institute for Medical Epidemiology, Biostatistics and Informatics, University of Halle, Halle, Germany

[4]Department of Epidemiology, Helmholtz Centre for Infection Research, Braunschweig, Germany

[5]German Center for Infection Research, Hannover-Braunschweig site, Germany

[6]Center for Health Economics Research Hannover (CHERH), Leibniz Universität Hannover, Hanover, Germany

[7]Biomedical Research in End-Stage and Obstructive Lung Disease Hannover (BREATH), German Center for Lung Research (DZL), Hanover, Germany

Corresponding author

Veronika K. Jaeger, PhD

Institute of Epidemiology and Social Medicine

University of Münster

Domagkstraße 3

48149 Münster

veronika.jaeger@ukmuenster.de

In some rare cases, participants did not enter one contact per line in the contact diary, but instead entered "5 colleagues" or "grandparents". In this case, if the number of additional contacts is known (e.g. "3 colleagues"), we considered 3 contacts (in the same age group/contact setting/physical contact as the original contact) instead of 1 contact.

Example

Consider a hypothetical participant, J, who reported "3 colleagues" as one contact in the age group "30-40 years". J had contact a non-skin-to-skin contact with them at work. In this case, we considered that participant J had 3 contacts, each contact aged "30-40 years", met at work and non-physical.

If the number of additional contacts is unknown, e.g. the participant only stated "friends" or "grandparents", we assumed the number of contacts provided in Table 1.

Table 1. Assumed number of additional contacts**.**

Work colleagues/colleagues	Assumed 10
Friends	Assumed 5
Patients	Assumed 20
School class	Assumed 15
Physician's assistants	Assumed 3
Residents of the nursing home	Assumed 20
Persons cared for at work	Assumed 20
Kindergarten	Assumed 15
Clients	Assumed 30
Grandchildren	Assumed 3
Supermarket	Assumed 5
Suppliers	Assumed 5
Children	Assumed 3
Persons from my rented apartment	Assumed 3
Bank employee	Assume 2
