# Supplementary material for: Individual social contact data and population mobility data as early markers of SARS-CoV-2 transmission dynamics during the first wave in Germany—an analysis based on the COVIMOD study
Source: BMC Med. 2021 Oct 14;19:271. doi: 10.1186/s12916-021-02139-6 (PMC8515158; doi:10.1186/s12916-021-02139-6)

Individual social contact data reflected SARS-CoV-2 transmission dynamics during the first wave in Germany better than population mobility data – an analysis based on the COVIMOD study.

Additional File 3: Additional results

Damilola Victoria Tomori[1], Nicole Rübsamen[1], Tom Berger[1], Stefan Scholz[2], Jasmin Walde[1], Ian Wittenberg[3], Berit Lange[4,5], Alexander Kuhlmann[6,7], Johannes Horn[3], Rafael Mikolajczyk[3], Veronika K Jaeger[1*], André Karch[1*]

[1]Institute of Epidemiology and Social Medicine, University of Münster, Münster, Germany

[2]Immunization Unit, Robert Koch-Institute, Berlin, Germany

[3]Institute for Medical Epidemiology, Biostatistics and Informatics, University of Halle, Halle, Germany

[4]Department of Epidemiology, Helmholtz Centre for Infection Research, Braunschweig, Germany

[5]German Center for Infection Research, Hannover-Braunschweig site, Germany

[6]Center for Health Economics Research Hannover (CHERH), Leibniz Universität Hannover, Hanover, Germany

[7]Biomedical Research in End-Stage and Obstructive Lung Disease Hannover (BREATH), German Center for Lung Research (DZL), Hanover, Germany

Corresponding author

Veronika K. Jaeger, PhD

Institute of Epidemiology and Social Medicine

University of Münster

Domagkstraße 3

48149 Münster

veronika.jaeger@ukmuenster.de

*contributed equally as last authors

Patient characteristics in COVIMOD compared to the German population 3
1. WEIGHTED ANALYSIS INCLUDING GROUP CONTACTS.....................................4
1.1. Participant characteristics...4
1.2. Number of social contacts 5
1.3. Comparison of the relative reduction in transmission dynamics.....................................8
1.4. Boxplots of the number of overall contacts..12
1.5. Social contact patterns..14
2. UNWEIGHTED ANALYSIS INCLUDING GROUP CONTACTS.............................16
2.1. Participant characteristics...16
2.2. Number of social contacts 18
2.3. Comparison of the relative reduction in transmission dynamics....................................21
2.5. Social contact patterns..29
2.6. Relative reductions in transmission dynamics of SARS-CoV-2 31
3. WEIGHTED ANALYSIS WITHOUT GROUP CONTACTS 33
3.2. Number of social contacts 33
3.3. Comparison of the relative reduction in transmission dynamics...................................35
3.4. Boxplots of the number of overall contacts in various settings......................................38
3.5. Social contact patterns..43
3.6. Relative reductions in transmission dynamics of SARS-CoV-2 44
4. UNWEIGHTED ANALYSIS WITHOUT GROUP CONTACTS 46
4.2. Number of social contacts 46
4.3. Comparison of the relative reduction in transmission dynamics....................................49
4.4. Boxplots of the number of overall in various settings..52
4.5. Social contact patterns..57
4.6. Relative reductions in transmission dynamics of SARS-CoV-2 58

Patient characteristics in COVIMOD compared to the German population

Table 1. Participant characteristics in the COVIMOD survey Waves 1 to 4 and in the POLYMOD survey, in comparison with the German population

Data of the German population are based on https://www.zensus2011.de/DE/Zensus2011/zensus2011_node.html

	German population	POLYMOD	COVIMOD			
			Wave 1	Wave 2	Wave 3	Wave 4
**Age category**						
0 – 9	8.6%	14.0%	6.0%	5.7%	4.7%	6.3%
10 – 19	9.9%	17.8%	10.8%	9.6%	8.4%	10.3%
20 – 29	12.1%	14.8%	9.6%	7.9%	6.2%	11.0%
30 – 39	11.8%	9.7%	11.8%	12.0%	9.3%	10.4%
40 – 49	16.6%	14.0%	8.2%	9.1%	8.8%	8.9%
50 – 59	14.5%	12.8%	19.7%	20.2%	22.2%	18.6%
60 - 69	11.1%	12.2%	25.3%	26.7%	29.6%	25.2%
70 - 79	10.1%	3.6%	7.9%	7.9%	10.0%	8.8%
80 +	5.3%	1.0%	0.7%	0.8%	0.7%	0.5%
**Sex of participants**						
Female	48.8%	55.4%	48.1%	47.1%	49.6%	47.8%
Male	51.2%	44.6%	51.9%	52.9%	50.4%	52.2%
**Household size**						
1	17.1%	18.6%	14.9%	18.9%	24.8%	25.8%
2	31.1%	30.6%	26.4%	25.9%	25.0%	23.2%
3	20.1%	25.3%	32.9%	33.0%	31.7%	28.8%
4	19.4%	18.2%	12.8%	11.1%	9.9%	11.3%
5	7.6%	4.8%	9.6%	8.3%	6.6%	8.1%
6 or more	4.7%	2.4%	3.4%	2.9%	2.0%	2.9%

1. WEIGHTED ANALYSIS INCLUDING GROUP CONTACTS

COVIMOD Wave 3 - 4 as well as POLYMOD include group contacts

1.1. Participant characteristics

1.1.a. Table 1.1a Participant characteristics in the COVIMOD survey Waves 1 to 4 and in the POLYMOD survey for the weighted analysis including group contacts

	POLYMOD	COVIMOD			
		Wave 1	Wave 2	Wave 3	Wave 4
	percent	percent	percent	percent	percent
**Age category**					
0 - 4	1.9	4.7	3.4	2.4	4.1
5 - 9	4.5	3.7	5.1	5.9	5.5
10 - 14	2.8	3.2	3.9	4.1	5.0
15 - 19	2.5	7.6	6.8	5.6	5.6
20 - 24	9.4	7.8	6.6	3.6	5.5
25 - 34	6.8	13.5	13.1	18.4	16.7
35 - 44	11.8	17.0	14.8	13.3	15.0
45 - 54	21.5	18.0	19.2	12.7	16.9
55 - 64	6.2	7.3	7.5	10.4	8.3
65 - 69	6.2	5.2	6.0	5.6	4.2
70 - 74	8.8	6.8	6.2	10.9	7.2
75 - 79	1.8	2.8	3.9	2.9	3.3
80 +	15.8	2.4	3.5	4.2	2.6
**Sex of participants**					
Female	41.4	50.1	50.7	54.0	51.1
Male	58.6	49.9	49.3	46.0	48.9
**Household size**					
1	14.1	15.8	17.1	20.7	17.7
2	22.5	29.0	26.2	29.9	26.4
3	11.9	23.3	23.9	18.9	22.5
4 or more	51.5	31.9	32.8	30.4	33.4
**Weekdays**					
Monday	14.3	3.8	3.7	9.5	31.9
Tuesday	11.2	4.5	8.0	8.3	12.5
Wednesday	10.2	3.2	13.8	4.9	8.8
Thursday	26.7	50.0	38.0	31.0	11.1
Friday	13.4	5.6	6.9	9.5	7.1
Saturday	11.9	8.1	10.9	20.7	7.6
Sunday	12.3	24.8	18.6	16.0	21.1

1.2. Number of social contacts

1.2.a. Table 1.2a Number of recorded overall contacts per participant per day stratified by age, gender, household size, and day of the week in the COVIMOD survey Waves 1 to 4 and the POLYMOD survey for the weighted analysis including group contacts.

	**POLYMOD**		**COVIMOD**							
			**Wave 1**		**Wave 2**		**Wave 3**		**Wave 4**	
	**Mean (SD)**	**Min, Max**	**Mean (SD)**	**Min, Max**	**Mean (SD)**	**Min, Max**	**Mean (SD)**	**Min, Max**	**Mean (SD)**	**Min, Max**
	**18.9 (24.6)**	**1,512**	**2.0 (1.9)**	**0, 16**	**3.3 (4.7)**	**0,102**	**6.2 (18.4)**	**0,500**	**6.9 (26.3)**	**0,674**
**Age category**										
0 - 4	9.4 (5.5)	2,30	2.9 (1.6)	0,8	5.0 (2.6)	0,14	6.0 (6.5)	2,24	9.4 (14.0)	0,89
5 - 9	8.4 (5.0)	2,43	3.5 (1.8)	0,8	3.9 (3.3)	0,17	5.4 (5.3)	0,27	4.9 (4.4)	0,31
10 - 14	15.7 (15.6)	2,61	3.2 (1.6)	0,12	3.9 (2.2)	0,9	15.1 (51.4)	0,500	8.8 (12.7)	0,110
15 - 19	30.9 (25.4)	1,215	3.2 (2.0)	0,8	4.4 (3.2)	0,20	3.7 (3.7)	0,27	6.8 (13.3)	0,113
20 - 24	22.6 (23.7)	1,310	2.0 (2.2)	0,16	3.3 (2.7)	0,20	5.2 (8.6)	0,45	8.8 (20.4)	0,169
25 - 34	40.5 (41.4)	2,512	1.7 (1.8)	0,13	3.0 (3.2)	0,18	6.0 (13.1)	0,100	7.2 (16.6)	0,150
35 - 44	25.4 (16.6)	1,111	2.1 (2.0)	0,11	3.3 (2.8)	0,33	8.3 (22.7)	0,150	5.0 (29.7)	0,674
45 - 54	29.3 (31.8)	1,260	1.8 (1.5)	0,9	3.4 (8.2)	0,102	11.3 (27.9)	0,131	9.5 (48.3)	0,532
55 - 64	14.0 (20.8)	1,150	1.8 (1.5)	0,12	3.3 (4.0)	0,42	3.8 (9.9)	0,186	10.3 (24.8)	0,127
65 - 69	10.0 (7.9)	1,58	1.6 (1.7)	0,11	3.4 (6.6)	0,93	4.1 (9.3)	0,101	4.8 (17.5)	0,216
70 - 74	8.4 (8.7)	1,25	1.1 (1.4)	0,8	2.6 (3.1)	0,25	3.0 (2.1)	0,13	3.1 (4.5)	0,37
75 - 79	5.0 (4.0)	1,14	1.5 (1.0)	0,5	2.6 (1.7)	0,9	2.8 (4.1)	0,32	2.1 (1.8)	0,17
80 +	4.2 (2.0)	2,8	0.7 (1.0)	0,3	0.7 (1.9)	0,9	0.2 (0.8)	0,7	0.5 (1.1)	0,4
**Sex of participants**										
Female	21.3 (31.5)	1,512	2 (1.9)	0,16	3.4 (5.5)	0,102	6.6 (18.6)	0,186	6.5 (28.1)	0,493
Male	17.3 (18.0)	1,310	2 (1.8)	0,12	3.2 (3.7)	0,93	5.7 (18.2)	0,500	7.3 (24.3)	0,674
**Household size**										
1	11.0 (18.3)	1,158	0.7 (1.3)	0,12	1.6 (2.4)	0,42	2.8 (9.0)	0,150	5.3 (30.3)	0,674
2	10.1 (19.9)	1,512	1.3 (1.5)	0,11	3.3 (7.6)	0,101	8.4 (24.0)	0,132	4.9 (17.8)	0,532
3	16.7 (23.7)	1,310	2.0 (1.4)	0,11	3.3 (3.5)	0,102	4.1 (7.9)	0,186	8.0 (40.3)	0,493
4 or more	25.5 (26.2)	2,260	3.4 (1.8)	0,16	4.1 (2.9)	0,32	7.5 (20.9)	0,500	8.6 (15.2)	0,109

	POLYMOD		COVIMOD							
			Wave 1		Wave 2		Wave 3		Wave 4	
	**Mean (SD)**	**Min, Max**	**Mean (SD)**	**Min, Max**	**Mean (SD)**	**Min, Max**	**Mean (SD)**	**Min, Max**	**Mean (SD)**	**Min, Max**
	**18.9 (24.6)**	**1,512**	**2.0 (1.9)**	**0, 16**	**3.3 (4.7)**	**0,102**	**6.2 (18.4)**	**0,500**	**6.9 (26.3)**	**0,674**
**Weekdays**										
Monday	19.1 (14.3)	1,124	2.6 (1.7)	0,6	3.6 (3.2)	0,24	7.2 (14.2)	0,60	8.0 (26.8)	0,674
Tuesday	23.7 (36.3)	1,512	2.8 (2.4)	0,8	4.1 (5.2)	0,102	6.0 (6.8)	0,32	15.4 (54.9)	0,493
Wednesday	16.1 (23.3)	1,158	3.3 (3.2)	0,13	3.0 (3.5)	0,42	8.1 (16.3)	0,80	6.2 (14.2)	0,109
Thursday	11.5 (13.5)	1,260	1.9 (1.8)	0,16	3.2 (3.8)	0,93	8.4 (23.6)	0,186	3.9 (11.0)	0,216
Friday	22.5 (35.3)	1,162	2.1 (2.1)	0,8	4.4 (3.9)	0,32	9.0 (35.0)	0,500	4.8 (15.7)	0,238
Saturday	22.7 (27.0)	1,215	1.8 (1.4)	0,8	4.0 (9.8)	0,101	2.9 (2.5)	0,33	6.0 (11.9)	0,95
Sunday	24.5 (19.0)	1,96	1.9 (1.5)	0,12	2.5 (2.6)	0,21	3.2 (6.7)	0,99	3.1 (8.6)	0,126

1.2.b. Table 1.2.b Number of recorded overall contacts per different settings in the COVIMOD survey Waves 1 to 4 and the POLYMOD survey for the weighted analysis including group contacts.

Note: *The displayed educational contacts are based on the group of participants who attended an educational facility (kindergarten, school, university) and work contacts are based on the group of participants who reported to work full-/part-time*

	**POLYMOD**		**COVIMOD**							
			**Wave 1**		**Wave 2**		**Wave 3**		**Wave 4**	
	**Mean (SD)**	**Min, Max**	**Mean (SD)**	**Min, Max**	**Mean (SD)**	**Min, Max**	**Mean (SD)**	**Min, Max**	**Mean (SD)**	**Min, Max**
Overall	18.9 (24.6)	1,512	2.0 (1.9)	0,16	3.3 (4.7)	0,102	6.2 (18.4)	0,500	6.9 (26.3)	0,674
Home	3.3 (2.3)	0,26	1.6 (1.5)	0,12	1.8 (1.7)	0,9	1.6 (1.5)	0,13	1.6 (1.6)	0,23
Educational	1.8 (3.8)	0,42	0.0 (0.2)	0,3	0.2 (0.9)	0,10	0.1 (1.0)	0,17	0.7 (3.0)	0,60
Work	20.3 (29.5)	0,509	0.2 (0.9)	0,11	1.5 (6.1)	0,100	5.6 (20.4)	0,140	5.6 (30.9)	0,491
Transport	0.6 (1.7)	0,8	0.0 (0.2)	0,3	0.1 (0.4)	0,4	0.0 (0.2)	0,8	0.1 (0.5)	0,12
Others	3.4 (4.6)	0,45	0.4 (1.0)	0,10	0.9 (1.8)	0,33	1.5 (4.9)	0,149	2.1 (14.1)	0,674

1.3. Comparison of the relative reduction in transmission dynamics

1.3.a. Table 1.3a Comparison of the relative reduction in transmission dynamics based on different input data. Given are the mean and bootstrapped 95% confidence interval of the relative percentage reduction of the R estimates from the RKI compared to the basic reproduction number and the relative reduction in the number of social contacts from COVIMOD compared to estimates from POLYMOD as well as of mobility estimates compared to pre-pandemic mobility for the weighted analysis including group contact.

	**COVIMOD**	**R (RKI)**	**Google**	**Apple**
	**Relative Mean Percentage Reduction (95%CI)**			
**Overall**				
30th April - 6th May 2020	89.30 (86.44,91.71)	67.00 (69.00,64.00)	24.31 (36.37,17.66)	36.77 (41.31, 32.79)
14th May - 21st May 2020	82.56 (77.78,86.32)	66.00 (69.00,62.00)	16.85 (26.87, 9.80)	15.65 (19.90, 10.67)
28th May - 4th June 2020	67.32 (53.40,78.36)	60.00 (64.00,55.00)	13.05 (23.80, 5.65)	-5.18 (-0.74,-10.19)
11th June - 22nd June 2020	63.23 (48.75,74.27)	68.00 (71.00,65.00)	10.63 (16.25, 6.28)	-11.65 (-8.31,-15.99)
**Home**				
30th April - 6th May 2020	52.78 (44.43,59.60)	-	-12.71 (-9.14,-18.42)	-
14th May - 21st May 2020	45.80 (34.90,54.45)	-	-8.62 (-6.00,-11.62)	-
28th May - 4th June 2020	49.89 (40.56,58.24)	-	-6.88 (-3.88,-10.12)	-
11th June - 22nd June 2020	52.27 (44.11,59.42)	-	-5.25 (-3.17, -7.91)	-
**Educational**				
30th April - 6th May 2020	97.97 (94.07,99.73)	-	-	-
14th May - 21st May 2020	86.52 (66.17,96.21)	-	-	-
28th May - 4th June 2020	91.58 (75.25,98.69)	-	-	-
11th June - 22nd June 2020	57.11 (9.51,83.37)	-	-	-
**Work**				
30th April - 6th May 2020	98.77 (97.80,99.39)	-	34.71 (52.42,23.58)	-
14th May - 21st May 2020	92.52 (87.66,95.69)	-	27.00 (45.10,13.87)	-
28th May - 4th June 2020	71.53 (43.22,89.40)	-	24.12 (42.00, 9.00)	-
11th June - 22nd June 2020	72.10 (46.45,86.23)	-	16.50 (28.50, 5.17)	-

	COVIMOD	R (RKI)	Google	Apple
	**Relative Mean Percentage Reduction (95%CI)**			
**Transport**				
30th April - 6th May 2020	91.16 (74.80,97.95)	-	42.14 (50.85,36.86)	-
14th May - 21st May 2020	76.52 (27.74,94.47)	-	31.75 (36.38,27.88)	-
28th May - 4th June 2020	91.15 (73.46,97.98)	-	25.12 (31.12,19.63)	-
11th June - 22nd June 2020	72.02 (23.62,93.37)	-	23.00 (27.00,19.67)	-
**Others**				
30th April - 6th May 2020	86.70 (80.86,91.55)	-	28.71 (49.35,16.14)	-
14th May - 21st May 2020	71.41 (57.60,81.67)	-	17.06 (34.31, 3.94)	-
28th May - 4th June 2020	54.29 (29.00,72.34)	-	11.44 (27.37, 0.32)	-
11th June - 22nd June 2020	34.23 (-3.36,60.00)	-	9.46 (17.50, 3.09)	-

1.3.b. Table 1.3b Effective reproduction number at the timing of COVIMOD survey Waves 1 to 4, estimated effective reproduction number based on the reduction of social contacts at the times of COVIMOD survey Waves 1 to 4 assuming values of the basic reproduction number of Norm (2.6, SD=0.54) with 10000 bootstrapped samples in various settings and in different time frames for the weighted analysis including group contacts

	**COVIMOD**		**R (RKI)**	
	**Mean reproduction number (95%CI)**	**Percent Mean Reduction (95%CI)**	**Mean reproduction number (95%CI)**	**Percent Mean Reduction (95%CI)**
**Overall**				
30th April - 6th May 2020	0.25 (0.14,0.36)	91.00 (95.00,87.00)	0.88 (0.82,0.94)	67.00 (69.00,64.00)
14th May - 21st May 2020	0.36 (0.21,0.53)	87.00 (92.00,80.00)	0.91 (0.81,1.00)	66.00 (69.00,62.00)
28th May - 4th June 2020	0.54 (0.29,0.83)	80.00 (89.00,69.00)	1.06 (0.94,1.19)	60.00 (64.00,55.00)
11th June - 22nd June 2020	0.68 (0.35,1.18)	74.00 (87.00,55.00)	0.84 (0.76,0.91)	68.00 (71.00,65.00)
**Home**				
30th April - 6th May 2020	1.39 (0.82,2.01)	47.00 (69.00,23.00)	-	-
14th May - 21st May 2020	1.37 (0.80,1.99)	48.00 (70.00,24.00)	-	-
28th May - 4th June 2020	1.44 (0.82,2.11)	45.00 (69.00,19.00)	-	-
11th June - 22nd June 2020	1.27 (0.74,1.84)	52.00 (72.00,30.00)	-	-
**Educational**				
30th April - 6th May 2020	0.05 (0.01,0.15)	99.00 (100.00,95.00)	-	-
14th May - 21st May 2020	0.26 (0.04,0.92)	90.00 (99.00,65.00)	-	-
28th May - 4th June 2020	0.14 (0.03,0.40)	95.00 (99.00,85.00)	-	-
11th June - 22nd June 2020	0.82 (0.14,2.76)	69.00 (95.00,-6.00)	-	-
**Work**				
30th April - 6th May 2020	0.04 (0.02,0.07)	99.00 (100.00,98.00)	-	-
14th May - 21st May 2020	0.14 (0.07,0.23)	95.00 (98.00,92.00)	-	-
28th May - 4th June 2020	0.32 (0.14,0.61)	88.00 (95.00,77.00)	-	-

	COVIMOD		R (RKI)	
	**Mean reproduction number (95%CI)**	**Percent Mean Reduction (95%CI)**	**Mean reproduction number (95%CI)**	**Percent Mean Reduction (95%CI)**
11th June - 22nd June 2020	0.33 (0.16,0.56)	88.00 (94.00,79.00)	-	-
**Transport**				
30th April - 6th May 2020	0.79 (0.12,2.87)	70.00 (96.00, -10.00)	-	-
14th May - 21st May 2020	1.11 (0.15,4.23)	58.00 (95.00, -62.00)	-	-
28th May - 4th June 2020	0.73 (0.11,2.70)	72.00 (96.00, -3.00)	-	-
11th June - 22nd June 2020	1.42 (0.17,6.25)	46.00 (94.00,-140.00)	-	-
30th April - 6th May 2020	0.29 (0.13,0.55)	89.00 (95.00, 79.00)	-	-
14th May - 21st May 2020	0.58 (0.27,1.09)	78.00 (90.00, 59.00)	-	-
28th May - 4th June 2020	1.07 (0.45,2.08)	59.00 (83.00, 20.00)	-	-
11th June - 22nd June 2020	1.36 (0.51,3.09)	48.00 (81.00,-18.00)	-	-

1.4. **Boxplots of the number of overall contacts**

Boxes represent the 25th, 50th and 75th percentiles, the whiskers represent the 10th and 90th percentile and the white dots represent the mean. Participants with no contacts are displayed as 0 on the log-scale of the y-axis.
Note: *The displayed work contacts are based on the group of participants who reported to work full-/part-time.*

1.4.a. Figure 1.4a Displayed are the number of social contacts stratified by sex (a) Female and (b) Male for the weighted analysis including group contacts in overall contacts.

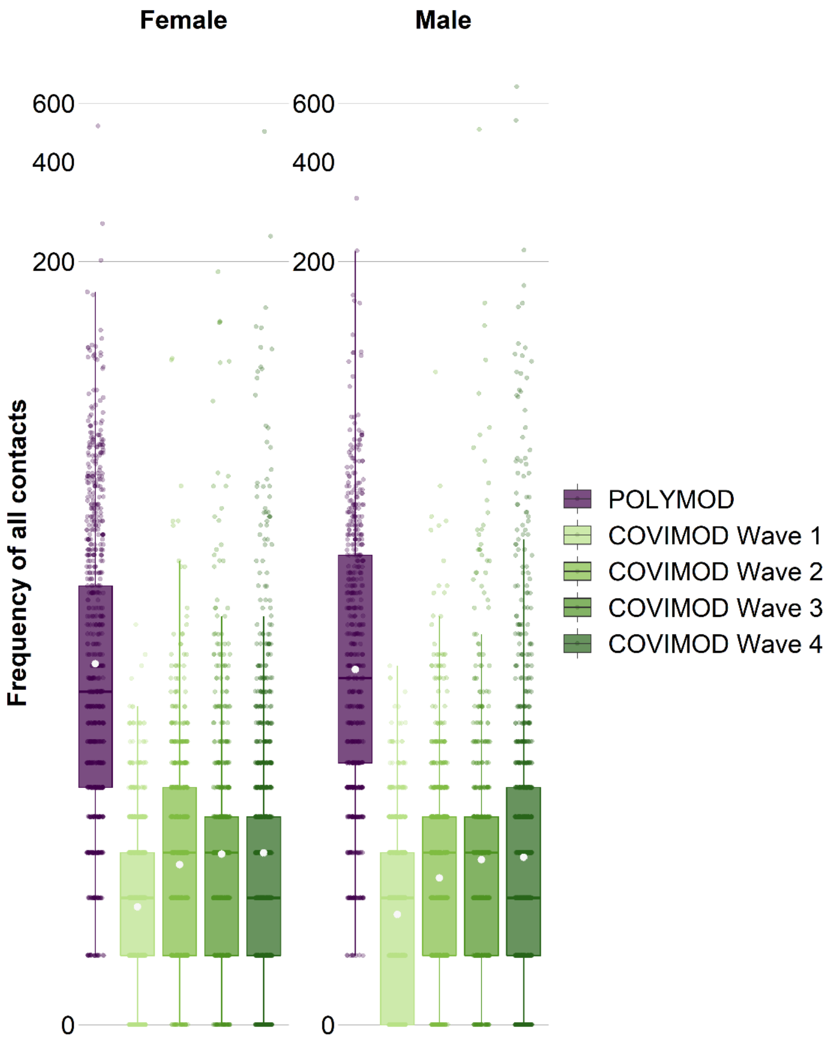

1.4.b. Figure 1.4b Displayed are the number of social contacts stratified by day of the week (a) Monday, (b) Tuesday, (c) Wednesday, (d) Thursday, (e) Friday, (f) Saturday and (g) Sunday for the weighted analysis including group contacts in overall contacts.

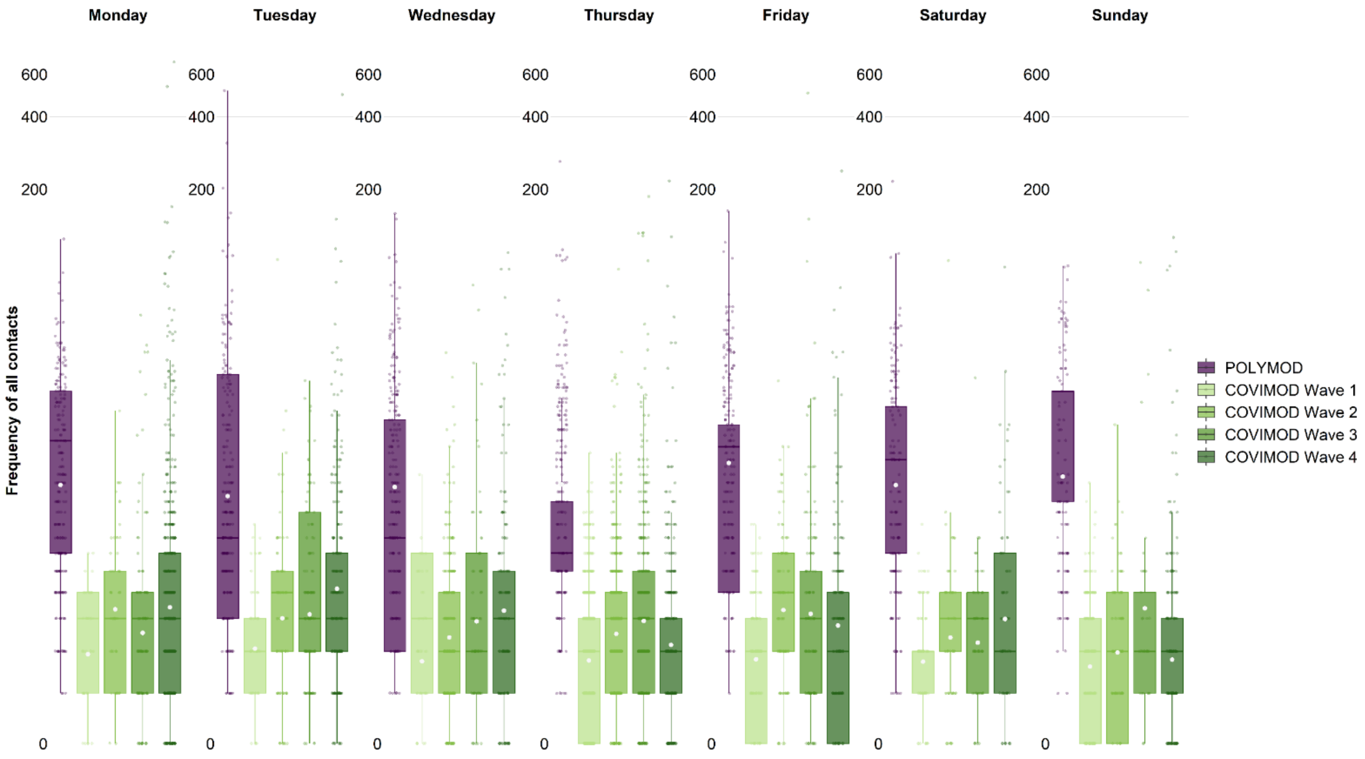

1.5. Social contact patterns

1.5. Figure 1.5 Social contact matrices with the mean total number of reported daily social contacts by participants in different age groups with individuals in other age groups in POLYMOD and COVIMOD survey waves 1 to 4 in various settings. Displayed are (a) the overall number of contacts, (b) home contacts, (c) educational contacts, (d) work contacts, (e) public transport contacts and (f) other contacts for the weighted analysis including group contacts.

Note: *Participants with more than more than 100 group contacts were removed in COVIMOD and POLYMOD. Specifically, 6 participants and 13 participants were removed in Wave 3 and Wave 4 respectively, while 10 participants were removed in POLYMOD.*

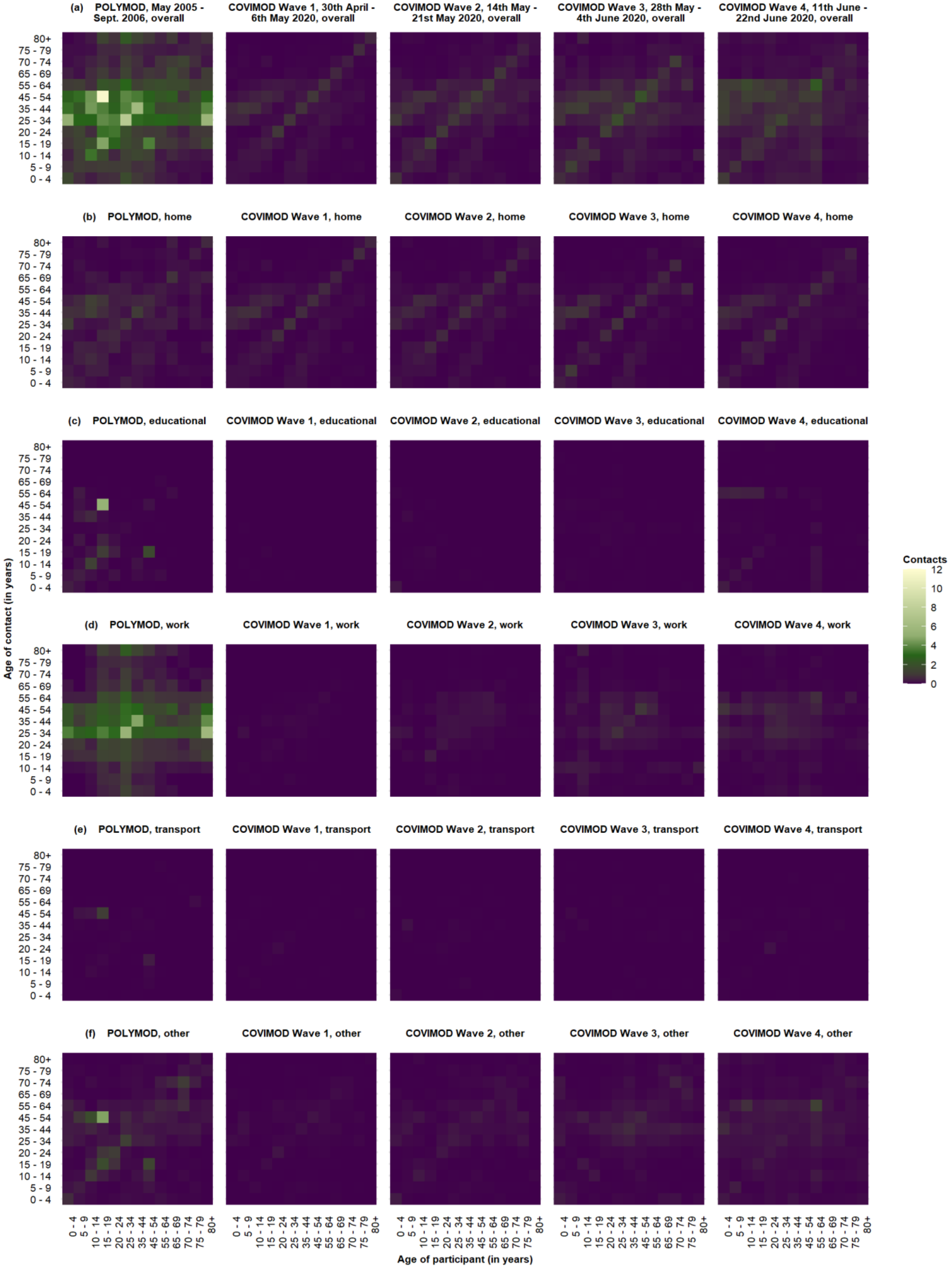
(a) POLYMOD, May 2005 - Sept. 2006, overall
COVIMOD Wave 1, 30th April - 6th May 2020, overall
COVIMOD Wave 2, 14th May - 21st May 2020, overall
COVIMOD Wave 3, 28th May - 4th June 2020, overall
COVIMOD Wave 4, 11th June - 22nd June 2020, overall
(b) POLYMOD, home
COVIMOD Wave 1, home
COVIMOD Wave 2, home
COVIMOD Wave 3, home
COVIMOD Wave 4, home
(c) POLYMOD, educational
COVIMOD Wave 1, educational
COVIMOD Wave 2, educational
COVIMOD Wave 3, educational
COVIMOD Wave 4, educational
(d) POLYMOD, work
COVIMOD Wave 1, work
COVIMOD Wave 2, work
COVIMOD Wave 3, work
COVIMOD Wave 4, work
(e) POLYMOD, transport
COVIMOD Wave 1, transport
COVIMOD Wave 2, transport
COVIMOD Wave 3, transport
COVIMOD Wave 4, transport
(f) POLYMOD, other
COVIMOD Wave 1, other
COVIMOD Wave 2, other
COVIMOD Wave 3, other
COVIMOD Wave 4, other
Age of contact (in years)
80+
75 - 79
70 - 74
65 - 69
55 - 64
45 - 54
35 - 44
25 - 34
20 - 24
15 - 19
10 - 14
5 - 9
0 - 4
Age of participant (in years)
Contacts
12
10
8
6
4
2
0

2. UNWEIGHTED ANALYSIS INCLUDING GROUP CONTACTS

COVIMOD Wave 3 - 4 as well as POLYMOD include group contacts

2.1. Participant characteristics

2.1.a. Table 2.1a Participant characteristics in the COVIMOD survey Waves 1 to 4 and in the POLYMOD survey for the unweighted analysis including group contacts

	POLYMOD		COVIMOD							
			Wave 1		Wave 2		Wave 3		Wave 4	
	N	percent	N	percent	N	percent	N	percent	N	percent
	**1341**	**-**	**1560**	**-**	**1356**	**-**	**1081**	**-**	**1890**	**-**
**Age category**										
0 - 4	89	6.9	46	2.9	36	2.7	21	1.9	56	3.0
5 - 9	92	7.1	48	3.1	41	3.0	30	2.8	62	3.3
10 - 14	110	8.5	73	4.7	63	4.7	45	4.2	87	4.6
15 - 19	121	9.3	95	6.1	66	4.9	45	4.2	108	5.7
20 - 24	117	9.0	83	5.3	60	4.4	28	2.6	109	5.8
25 - 34	132	10.2	173	11.1	148	10.9	96	8.9	219	11.6
35 - 44	156	12.0	137	8.8	124	9.2	91	8.4	164	8.7
45 - 54	184	14.2	235	15.1	209	15.4	174	16.1	275	14.6
55 - 64	160	12.4	265	17.0	244	18.0	237	21.9	321	17.0
65 - 69	74	5.7	270	17.3	245	18.1	199	18.4	313	16.6
70 - 74	33	2.5	89	5.7	73	5.4	79	7.3	118	6.2
75 - 79	14	1.1	35	2.2	34	2.5	29	2.7	48	2.5
80 +	13	1.0	11	0.7	11	0.8	7	0.6	10	0.5
Missing	46	-	0	-	2	-	0	-	0	-
**Sex of participants**										
Female	722	55.4	748	48.1	638	47.1	536	49.6	901	47.8
Male	581	44.6	806	51.9	717	52.9	544	50.4	985	52.2
Missing	38	-	6	-	1	-	1	-	4	-
**Household size**										
1	250	18.6	232	14.9	256	18.9	268	24.8	487	25.8
2	411	30.6	412	26.4	351	25.9	270	25.0	439	23.2
3	339	25.3	514	32.9	447	33.0	343	31.7	544	28.8
4 or more	341	25.4	402	25.8	302	22.3	200	18.5	420	22.2
Missing	0	-	0	-	0	-	0	-	0	-
**Weekdays**										
Monday	227	17.2	50	3.2	60	4.4	128	11.8	642	34.0
Tuesday	237	18.0	63	4.0	89	6.6	143	13.2	246	13.0
Wednesday	222	16.8	54	3.5	293	21.6	87	8.0	172	9.1
Thursday	179	13.6	914	58.6	613	45.2	489	45.2	320	16.9
Friday	186	14.1	144	9.2	117	8.6	132	12.2	196	10.4
Saturday	152	11.5	88	5.6	63	4.6	66	6.1	81	4.3

	POLYMOD		COVIMOD							
			Wave 1		Wave 2		Wave 3		Wave 4	
	**N**	**percent**	**N**	**percent**	**N**	**percent**	**N**	**percent**	**N**	**percent**
	**1341**	**-**	**1560**	**-**	**1356**	**-**	**1081**	**-**	**1890**	**-**
Sunday	117	8.9	247	15.8	121	8.9	36	3.3	233	12.3
Missing	21	-	0	-	0	-	0	-	0	-

2.2. **Number of social contacts**

2.2.a. Table 2.2a Number of recorded overall contacts per participant per day stratified by age, gender, household size, and day of the week in the COVIMOD survey Waves 1 to 4 and the POLY MOD survey for the unweighted analysis including group contacts.

	**POLYMOD**			**COVIMOD**											
				**Wave 1**			**Wave 2**			**Wave 3**			**Wave 4**		
	**N**	**Mean (SD)**	**Min, Max**	**N**	**Mean (SD)**	**Min, Max**	**N**	**Mean (SD)**	**Min, Max**	**N**	**Mean (SD)**	**Min, Max**	**N**	**Mean (SD)**	**Min, Max**
	**1341**	**20.2 (28.5)**	**1,512**	**1560**	**2.1 (1.9)**	**0,16**	**1356**	**3.6 (6.1)**	**0,102**	**1081**	**5.9 (20.3)**	**0,500**	**1890**	**7.1 (27.9)**	**0,674**
**Age category**															
0 - 4	89	9.1 (6.1)	2,30	46	3.8 (1.9)	0,8	36	4.7 (2.7)	0,14	21	7.3 (6.5)	2, 24	56	12.9 (29.2)	0,198
5 - 9	92	8.4 (5.7)	2,43	48	3.5 (1.7)	0,8	41	4.4 (3.2)	0,17	30	6.9 (6.7)	0, 27	62	6.4 (5.5)	0, 31
10 - 14	110	13.3 (12.6)	2,61	73	3.2 (1.8)	0,12	63	3.3 (2.0)	0,9	45	19.0 (76.1)	0,500	87	9.9 (17.7)	0,110
15 - 19	121	32.8 (28.6)	1,215	95	3.3 (2.0)	0,8	66	4.1 (3.7)	0,20	45	5.1 (6.0)	0, 27	108	11.1 (20.2)	0,113
20 - 24	117	29.1 (32.9)	1,310	83	2.0 (2.6)	0,16	60	3.0 (3.3)	0,20	28	5.7 (9.7)	0, 45	109	9.9 (23.6)	0,169
25 - 34	132	31.9 (50.9)	2,512	173	1.7 (1.8)	0,13	148	2.8 (3.2)	0,18	96	5.2 (12.7)	0,100	219	7.1 (19.0)	0,150
35 - 44	156	25.0 (21.7)	1,111	137	1.9 (2.2)	0,11	124	3.0 (3.7)	0,33	91	7.8 (22.3)	0,150	164	8.1 (53.2)	0,674
45 - 54	184	26.2 (35.1)	1,260	235	1.8 (1.7)	0,9	209	4.0 (10.3)	0,102	174	4.6 (11.3)	0,131	275	9.4 (47.0)	0,532
55 - 64	160	13.9 (19.5)	1,150	265	1.9 (1.7)	0,12	244	3.7 (5.1)	0,42	237	5.6 (16.1)	0,186	321	4.7 (11.6)	0,127
65 - 69	74	8.6 (10.6)	1,58	270	1.8 (1.7)	0,11	245	3.9 (7.6)	0,93	199	5.2 (12.5)	0,101	313	5.4 (16.9)	0,216
70 - 74	33	5.8 (5.7)	1,25	89	1.8 (1.5)	0,8	73	3.1 (3.5)	0,25	79	3.2 (2.5)	0, 13	118	3.1 (4.5)	0, 37
75 - 79	14	4.6 (3.7)	1,14	35	1.7 (1.4)	0,5	34	2.6 (2.1)	0,9	29	3.9 (5.9)	0, 32	48	3.2 (3.0)	0, 17
80 +	13	3.7 (1.8)	2,8	11	1.1 (1.0)	0,3	11	2.0 (2.8)	0,9	7	2.1 (2.4)	0, 7	10	1.7 (1.9)	0, 4
**Sex of participants**															
Female	722	21.4 (31.4)	1,512	748	2.2 (2.0)	0,16	638	3.9 (6.8)	0,102	536	5.7 (15.1)	0,186	901	6.5 (22.3)	0,493

	POLYMOD			COVIMOD											
				Wave 1			Wave 2			Wave 3			Wave 4		
	**N**	**Mean (SD)**	**Min, Max**	**N**	**Mean (SD)**	**Min, Max**	**N**	**Mean (SD)**	**Min, Max**	**N**	**Mean (SD)**	**Min, Max**	**N**	**Mean (SD)**	**Min, Max**
	**1341**	**20.2 (28.5)**	**1,512**	**1560**	**2.1 (1.9)**	**0,16**	**1356**	**3.6 (6.1)**	**0,102**	**1081**	**5.9 (20.3)**	**0,500**	**1890**	**7.1 (27.9)**	**0,674**
Male	581	19.1 (24.9)	1,310	806	2.0 (1.8)	0,12	717	3.3 (5.3)	0,93	544	6.0 (24.4)	0,500	985	7.5 (31.7)	0,674
**Household size**															
1	250	18.3 (23.7)	1,158	232	0.8 (1.5)	0,12	256	2.1 (4.2)	0,42	268	3.6 (11.2)	0,150	487	5.9 (34.6)	0,674
2	411	19.9 (34.1)	1,512	412	1.3 (1.6)	0,11	351	3.8 (8.4)	0,101	270	6.3 (17.6)	0,132	439	6.8 (30.4)	0,532
3	339	20.9 (27.9)	1,310	514	2.2 (1.5)	0,11	447	3.6 (6.0)	0,102	343	5.3 (12.6)	0,186	544	7.4 (27.2)	0,493
4 or more	341	21.3 (24.6)	2,260	402	3.5 (1.9)	0,16	302	4.5 (3.5)	0,32	200	9.3 (36.9)	0,500	420	8.6 (14.3)	0,109
**Weekdays**															
Monday	227	17.4 (17.2)	1,124	50	2.2 (1.7)	0,6	60	4.3 (4.1)	0,24	128	4.6 (9.2)	0, 60	642	8.7 (37.1)	0,674
Tuesday	237	22.0 (45.8)	1,512	63	2.4 (2.0)	0,8	89	5.0 (11.2)	0,102	143	4.6 (5.4)	0, 32	246	9.8 (34.8)	0,493
Wednesday	222	19.7 (23.8)	1,158	54	2.3 (2.5)	0,13	293	3.3 (4.4)	0,42	87	5.6 (11.9)	0, 80	172	7.2 (15.5)	0,109
Thursday	179	20.2 (27.8)	1,260	914	2.1 (1.9)	0,16	613	3.4 (5.5)	0,93	489	5.7 (15.8)	0,186	320	4.5 (15.6)	0,216
Friday	186	21.8 (22.7)	1,162	144	2.1 (1.8)	0,8	117	4.4 (4.5)	0,32	132	10.0 (46.0)	0,500	196	5.9 (19.1)	0,238
Saturday	152	19.5 (24.5)	1,215	88	2.0 (1.6)	0,8	63	4.2 (12.6)	0,101	66	2.9 (4.1)	0, 33	81	5.6 (11.7)	0, 95
Sunday	117	20.9 (21.9)	1,96	247	1.9 (1.9)	0,12	121	2.5 (2.8)	0,21	36	8.8 (20.7)	0, 99	233	5.1 (19.1)	0,198

2.2.b. Table 2.2.b Number of recorded overall contacts per different settings in the COVIMOD survey Waves 1 to 4 and the POLYMOD survey for the unweighted analysis including group contacts.

Note: *The displayed educational contacts are based on the group of participants who attended an educational facility (kindergarten, school, university) and work contacts are based on the group of participants who reported to work full-/part-time*

	**POLYMOD**			**COVIMOD**											
				**Wave 1**			**Wave 2**			**Wave 3**			**Wave 4**		
	**N**	**Mean (SD)**	**Min, Max**	**N**	**Mean (SD)**	**Min, Max**	**N**	**Mean (SD)**	**Min, Max**	**N**	**Mean (SD)**	**Min, Max**	**N**	**Mean (SD)**	**Min, Max**
Overall	1341	20.2 (28.5)	1,512	1560	2.1 (1.9)	0,16	1356	3.6 (6.1)	0,102	1081	5.9 (20.3)	0,500	1890	7.1 (27.9)	0,674
Home	1341	2.8 (2.3)	0,26	1560	1.6 (1.5)	0,12	1356	1.6 (1.6)	0,9	1081	1.5 (1.5)	0,13	1890	1.5 (1.7)	0,23
Educational	199	2.8 (4.7)	0,42	310	0.0 (0.2)	0,3	247	0.2 (1.0)	0,10	179	0.3 (1.9)	0,17	385	1.2 (4.3)	0,60
Work	715	18.5 (33.2)	0,509	690	0.4 (1.2)	0,11	613	2.0 (7.2)	0,100	476	4.0 (15.1)	0,140	809	4.6 (23.0)	0,491
Transport	1341	0.3 (0.8)	0,8	1560	0.0 (0.3)	0,3	1356	0.1 (0.4)	0,4	1081	0.1 (0.4)	0,8	1890	0.1 (0.6)	0,12
Others	1341	3.0 (3.8)	0,45	1560	0.4 (1.0)	0,10	1356	1.1 (2.4)	0,33	1081	1.9 (6.7)	0,149	1890	2.8 (21.2)	0,674

2.3. Comparison of the relative reduction in transmission dynamics

2.3.a. Table 2.3a Comparison of the relative reduction in transmission dynamics based on different input data. Given are the mean and bootstrapped 95% confidence interval of the relative percentage reduction of the R estimates from the RKI compared to the basic reproduction number and the relative reduction in the number of social contacts from COVIMOD compared to estimates from POLYMOD as well as of mobility estimates compared to pre-pandemic mobility for the unweighted analysis including group contact.

	**COVIMOD**	**R (RKI)**	**Google**	**Apple**
	**Relative Mean Percentage Reduction (95%CI)**			
**Overall**				
30th April - 6th May 2020	89.65 (88.77,90.57)	67.00 (69.00,64.00)	24.31 (36.37,17.66)	36.77 (41.31, 32.77)
14th May - 21st May 2020	82.34 (80.15,84.35)	66.00 (69.00,62.00)	16.85 (26.87, 9.80)	15.64 (19.91, 10.65)
28th May - 4th June 2020	70.73 (62.72,76.83)	60.00 (64.00,55.00)	13.05 (23.80, 5.65)	-5.18 (-0.74, -10.19)
11th June - 22nd June 2020	64.82 (57.75,71.02)	68.00 (71.00,65.00)	10.63 (16.25, 6.28)	-11.66 (-8.32, -15.99)
**Home**				
30th April - 6th May 2020	43.68 (39.85,47.19)	-	-12.71 (-9.14, -18.42)	-
14th May - 21st May 2020	43.38 (39.52,47.18)	-	-8.62 (-6.00, -11.62)	-
28th May - 4th June 2020	47.66 (43.63,51.76)	-	-6.88 (-3.88, -10.12)	-
11th June - 22nd June 2020	46.96 (43.31,50.57)	-	-5.25 (-3.17, -7.91)	-
**Educational**				
30th April - 6th May 2020	98.70 (97.40,99.66)	-	-	-
14th May - 21st May 2020	91.33 (85.70,95.63)	-	-	-
28th May - 4th June 2020	86.94 (73.96,95.88)	-	-	-
11th June - 22nd June 2020	57.38 (35.49,74.19)	-	-	-
**Work**				
30th April - 6th May 2020	98.10 (97.50,98.60)	-	34.71 (52.42,23.58)	-
14th May - 21st May 2020	89.18 (84.97,92.18)	-	27.00 (45.10,13.88)	-
28th May - 4th June 2020	78.18 (69.80,85.10)	-	24.12 (42.00, 9.00)	-
11th June - 22nd June 2020	75.04 (64.86,83.16)	-	16.50 (28.50, 5.17)	-
**Transport**				
30th April - 6th May 2020	83.09 (76.65,88.24)	-	42.14 (50.85,36.86)	-
14th May - 21st May 2020	71.15 (61.32,79.27)	-	31.75 (36.38,27.88)	-
28th May - 4th June 2020	75.02 (63.14,84.06)	-	25.12 (31.12,19.63)	-
11th June - 22nd June 2020	61.91 (49.32,73.07)	-	23.00 (27.00,19.67)	-
**Others**				
30th April - 6th May 2020	85.67 (83.65,87.43)	-	28.71 (49.35,16.14)	-
14th May - 21st May 2020	65.43 (60.51,69.75)	-	17.06 (34.31, 3.94)	-
28th May - 4th June 2020	38.89 (23.83,52.09)	-	11.44 (27.37, 0.32)	-
11th June - 22nd June 2020	7.93 (-28.09,34.95)	-	9.46 (17.50, 3.09)	-

2.3.b. Table 2.3b Effective reproduction number at the timing of COVIMOD survey Waves 1 to 4, estimated effective reproduction number based on the reduction of social contacts at the times of COVIMOD survey Waves 1 to 4 assuming values of the basic reproduction number of Norm (2.6, SD=0.54) and the reduction in mobility at the times of the COVIMOD survey Waves, with 10000 bootstrapped samples in various settings and in different time frames for the unweighted analysis including group contacts

	**COVIMOD**		**R (RKI)**	
	**Mean reproduction number (95%CI)**	**Percent Mean Reduction Number (95%CI)**	**Mean reproduction number (95%CI)**	**Percent Mean Reduction Number (95%CI)**
**Overall**				
30th April - 6th May 2020	0.32 (0.19,0.45)	88.00 (93.00,83.00)	0.88 (0.82,0.94)	67.00 (69.00,64.00)
14th May - 21st May 2020	0.45 (0.27,0.64)	83.00 (90.00,76.00)	0.90 (0.81,0.99)	66.00 (69.00,62.00)
28th May - 4th June 2020	0.66 (0.38,0.95)	75.00 (86.00,64.00)	1.06 (0.94,1.19)	60.00 (64.00,55.00)
11th June - 22nd June 2020	0.78 (0.46,1.13)	70.00 (83.00,57.00)	0.84 (0.76,0.91)	68.00 (71.00,65.00)
**Home**				
30th April - 6th May 2020	1.60 (0.96,2.28)	39.00 (64.00,13.00)	-	-
14th May - 21st May 2020	1.58 (0.93,2.24)	40.00 (65.00,14.00)	-	-
28th May - 4th June 2020	1.56 (0.91,2.25)	40.00 (65.00,14.00)	-	-
11th June - 22nd June 2020	1.41 (0.83,2.01)	46.00 (69.00,23.00)	-	-
**Educational**				
30th April - 6th May 2020	0.07 (0.02,0.16)	98.00 (100.00,94.00)	-	-
14th May - 21st May 2020	0.34 (0.12,0.76)	87.00 (96.00,71.00)	-	-
28th May - 4th June 2020	0.53 (0.16,1.23)	80.00 (94.00,53.00)	-	-
11th June - 22nd June 2020	1.24 (0.60,2.18)	53.00 (77.00,17.00)	-	-
**Work**				
30th April - 6th May 2020	0.05 (0.03,0.08)	99.00 (99.00,97.00)	-	-
14th May - 21st May 2020	0.18 (0.10,0.27)	94.00 (97.00,90.00)	-	-
28th May - 4th June 2020	0.36 (0.19,0.57)	87.00 (93.00,79.00)	-	-
11th June - 22nd June 2020	0.44 (0.25,0.66)	84.00 (91.00,75.00)	-	-
**Transport**				

	COVIMOD		R (RKI)	
	**Mean reproduction number (95%CI)**	**Percent Mean Reduction Number (95%CI)**	**Mean reproduction number (95%CI)**	**Percent Mean Reduction Number (95%CI)**
30th April - 6th May 2020	0.66 (0.31,1.16)	75.00 (89.00,56.00)	-	-
14th May - 21st May 2020	0.94 (0.45,1.65)	64.00 (83.00,37.00)	-	-
28th May - 4th June 2020	0.74 (0.33,1.35)	72.00 (88.00,49.00)	-	-
11th June - 22nd June 2020	1.22 (0.57,2.45)	54.00 (79.00, 6.00)	-	-
**Others**				
30th April - 6th May 2020	0.40 (0.23,0.64)	85.00 (92.00,76.00)	-	-
14th May - 21st May 2020	0.72 (0.43,1.04)	73.00 (84.00,60.00)	-	-
28th May - 4th June 2020	1.43 (0.79,2.24)	46.00 (70.00,14.00)	-	-
11th June - 22nd June 2020	1.53 (0.88,2.26)	42.00 (67.00,14.00)	-	-

2.4. **Boxplots of the number of overall contacts in various settings**

Boxes represent the 25th, 50th and 75th percentiles, the whiskers represent the 10th and 90th percentile and the white dots represent the mean. Participants with no contacts are displayed as 0 on the log-scale of the y-axis.

2.4.a. Figure 2.4a Displayed are the number of social contacts by setting: (a) the overall number of contacts, (b) home contacts, (c) educational contacts, (d) work contacts, (e) public transport contacts and (f) other contacts for the unweighted analysis including group contacts in overall contacts.

Note: *The displayed educational contacts are based on the group of participants who attended an educational facility (kindergarten, school, university) and work contacts are based on the group of participants who reported to work full-/part-time*

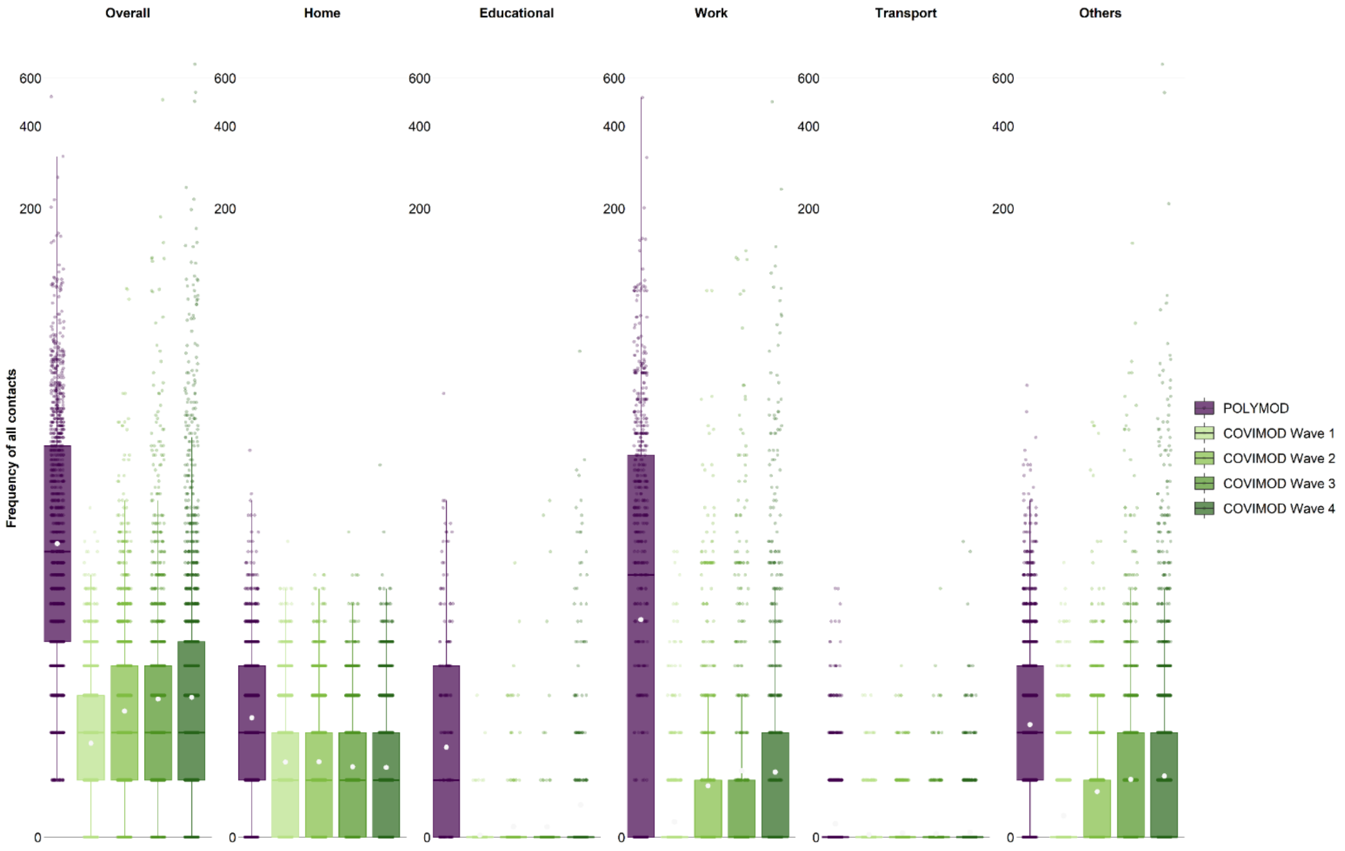

2.4.b. Figure 2.4b Displayed are the number of social contacts stratified by age (a) 0 - 7 years, (b) 8 – 15 years, (c) 16 – 23 years, (d) 24 – 38 years, (e) 39 – 60 years and (f) 61 years or more for the unweighted analysis including group contacts in overall contacts.

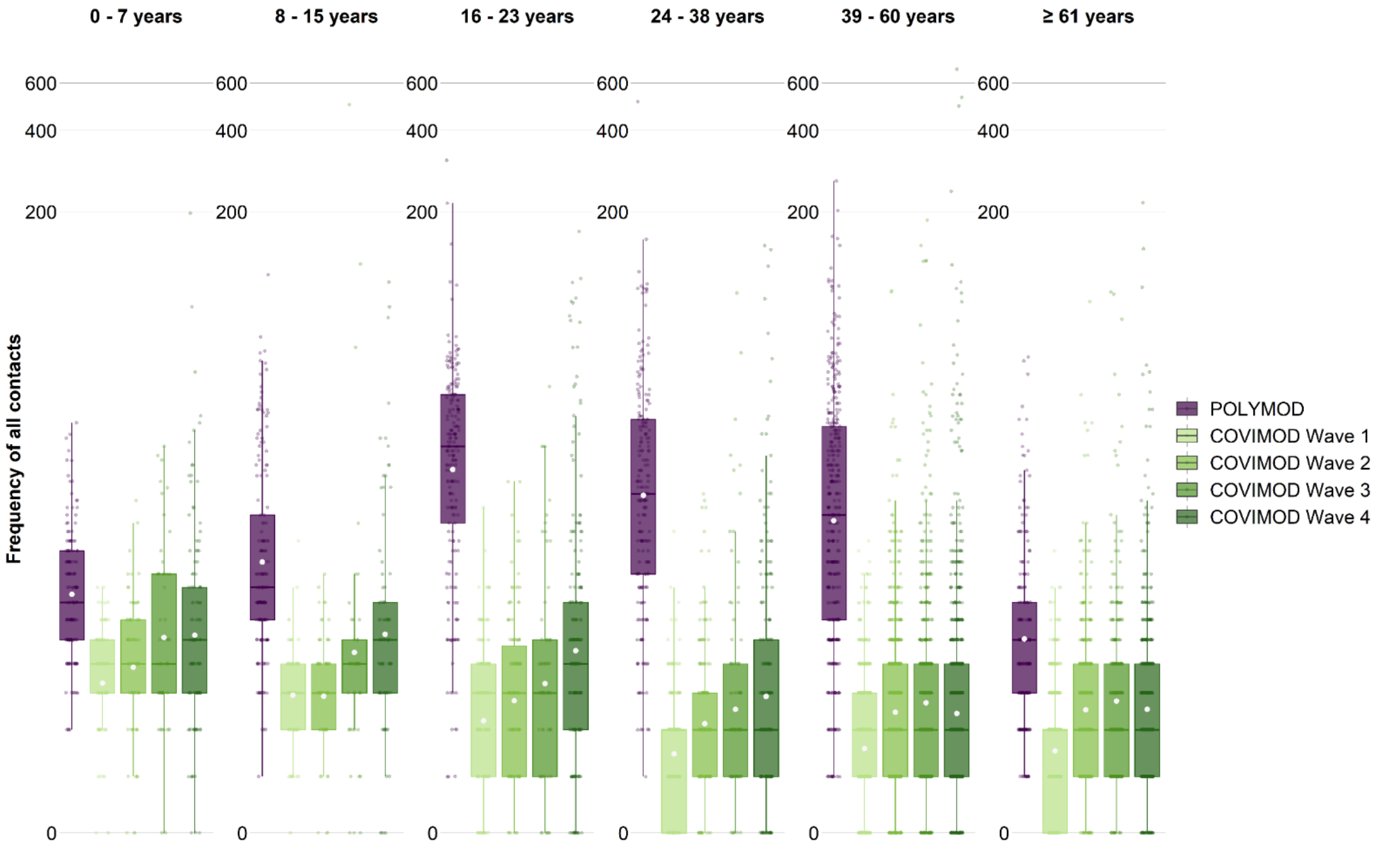

2.4.c. Figure 2.4c Displayed are the number of social contacts stratified by household size (a) Household size of 1, (b) Household size of 2, (c) Household size of 3 and (d) Household size of 4 or more for the unweighted analysis including group contacts in overall contacts.

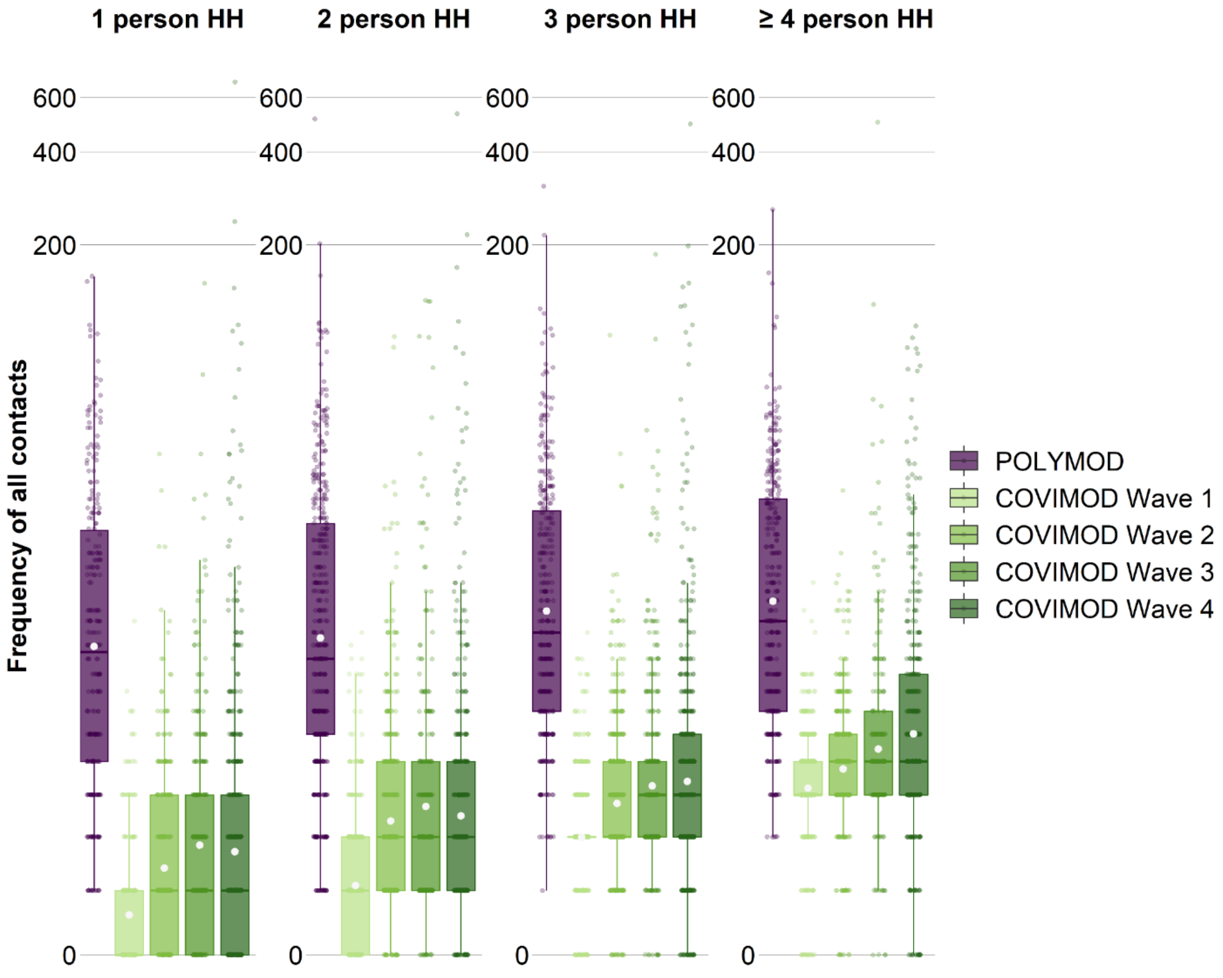

2.4.d. Figure 2.4d Displayed are the number of social contacts stratified by (a) Female and (b) Male for the unweighted analysis including group contacts in overall contacts.

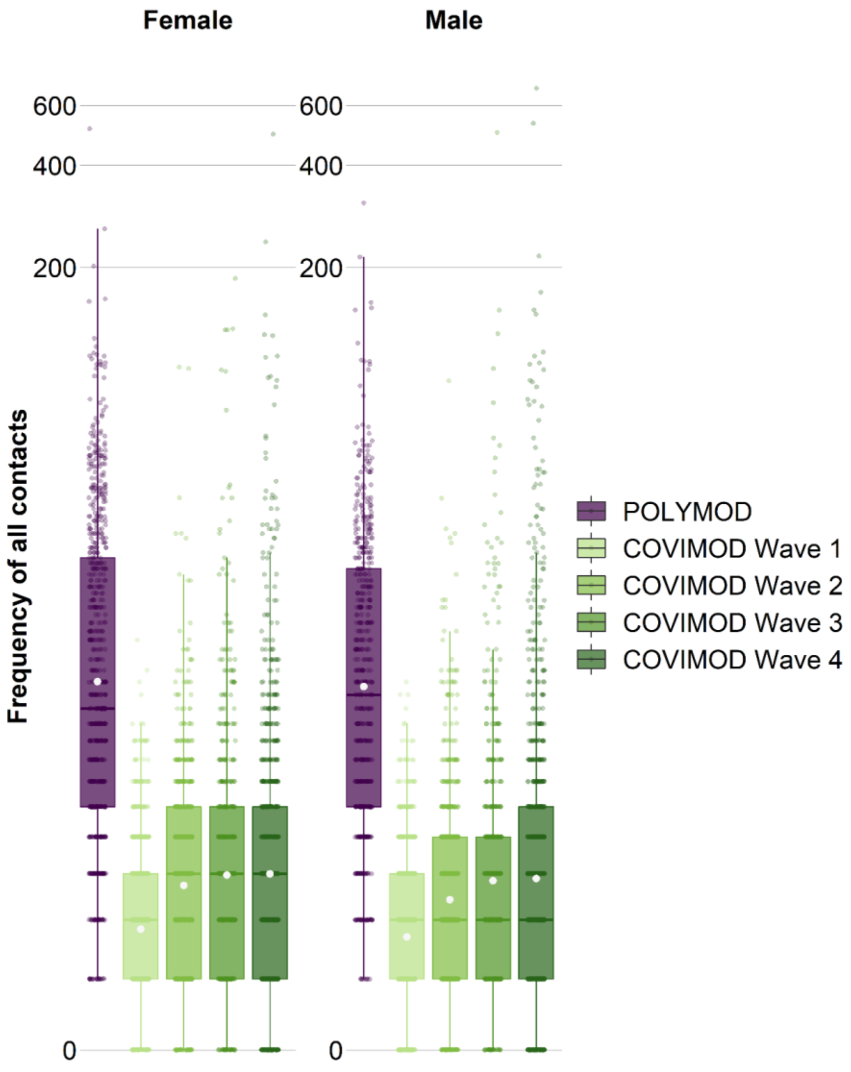

2.4.e. Figure 2.4e Displayed are the number of social contacts stratified by day of the week (a) Monday, (b) Tuesday, (c) Wednesday, (d) Thursday, (e) Friday, (f) Saturday and (g) Sunday for the unweighted analysis including group contacts in overall contacts.

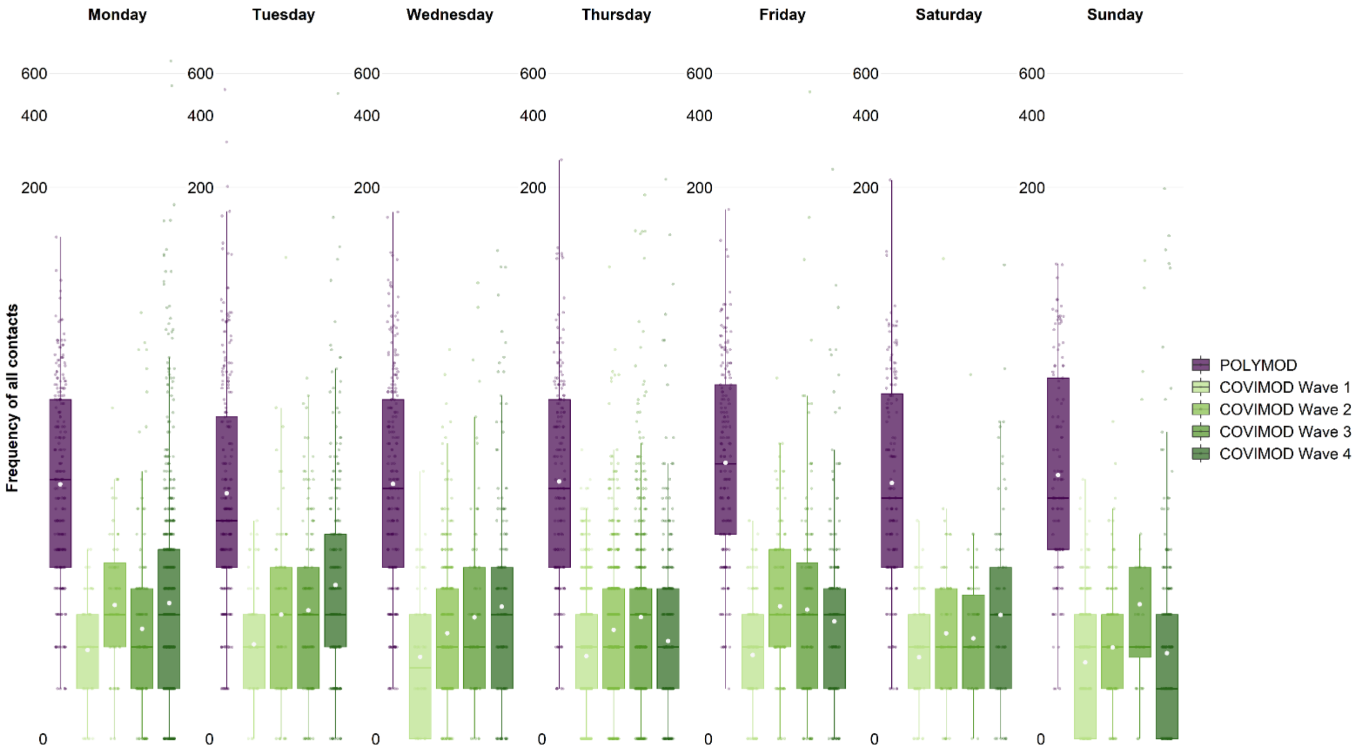

2.5. **Social contact patterns**

2.5.a. Figure 2.5a Social contact matrices with the mean total number of reported daily social contacts by participants in different age groups with individuals in other age groups in POLYMOD and COVIMOD survey waves 1 to 4 in various settings. Displayed are (a) the overall number of contacts, (b) home contacts, (c) educational contacts, (d) work contacts, (e) public transport contacts and (f) other contacts for the unweighted analysis including group contacts.

Note: *Participants with more than more than 100 group contacts were removed in COVIMOD and POLYMOD. Specifically, 6 participants and 13 participants were removed in Wave 3 and Wave 4 respectively, while 10 participants were removed in POLYMOD*

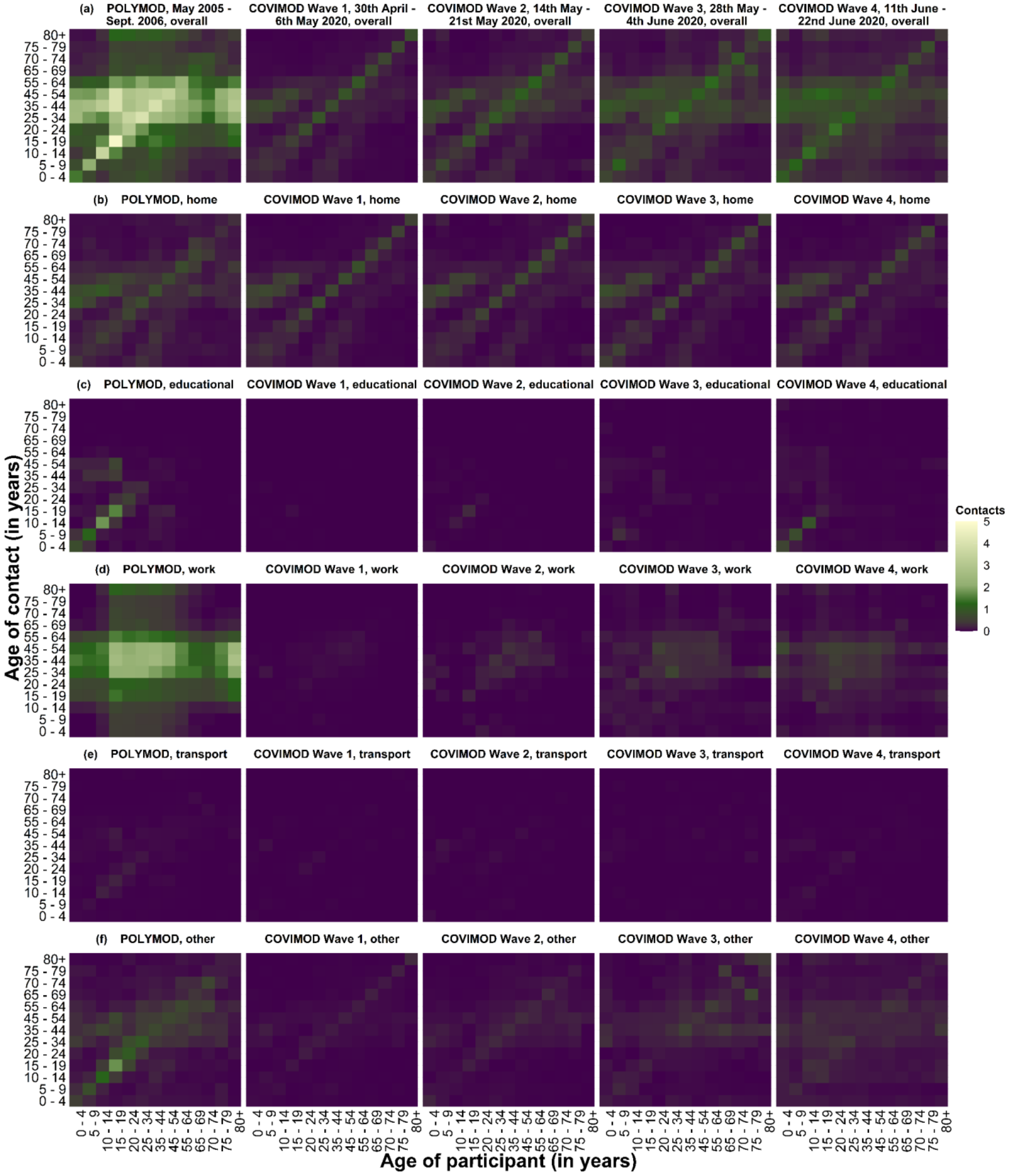

(a) POLYMOD, May 2005 - Sept. 2006, overall
COVIMOD Wave 1, 30th April - 6th May 2020, overall
COVIMOD Wave 2, 14th May - 21st May 2020, overall
COVIMOD Wave 3, 28th May - 4th June 2020, overall
COVIMOD Wave 4, 11th June - 22nd June 2020, overall
(b) POLYMOD, home
COVIMOD Wave 1, home
COVIMOD Wave 2, home
COVIMOD Wave 3, home
COVIMOD Wave 4, home
(c) POLYMOD, educational
COVIMOD Wave 1, educational
COVIMOD Wave 2, educational
COVIMOD Wave 3, educational
COVIMOD Wave 4, educational
(d) POLYMOD, work
COVIMOD Wave 1, work
COVIMOD Wave 2, work
COVIMOD Wave 3, work
COVIMOD Wave 4, work
(e) POLYMOD, transport
COVIMOD Wave 1, transport
COVIMOD Wave 2, transport
COVIMOD Wave 3, transport
COVIMOD Wave 4, transport
(f) POLYMOD, other
COVIMOD Wave 1, other
COVIMOD Wave 2, other
COVIMOD Wave 3, other
COVIMOD Wave 4, other
Age of contact (in years)
Age of participant (in years)
80+
75 - 79
70 - 74
65 - 69
55 - 64
45 - 54
35 - 44
25 - 34
20 - 24
15 - 19
10 - 14
5 - 9
0 - 4
Contacts
5
4
3
2
1
0

2.6. **Relative reductions in transmission dynamics of SARS-CoV-2**

2.6.a. Figure 2.6a Comparison of the relative reduction in transmission dynamics based on different input data.

Displayed are the mean and bootstrapped 95% confidence interval of the relative reduction of the R estimates from the RKI compared to the basic reproduction number and the relative reduction in the number of social contacts (COVIMOD-simple approach) as well as of mobility estimates compared to pre-pandemic times (A) displays the overall relative reduction and (B) displays the relative reduction by setting for the unweighted analysis including group contacts.

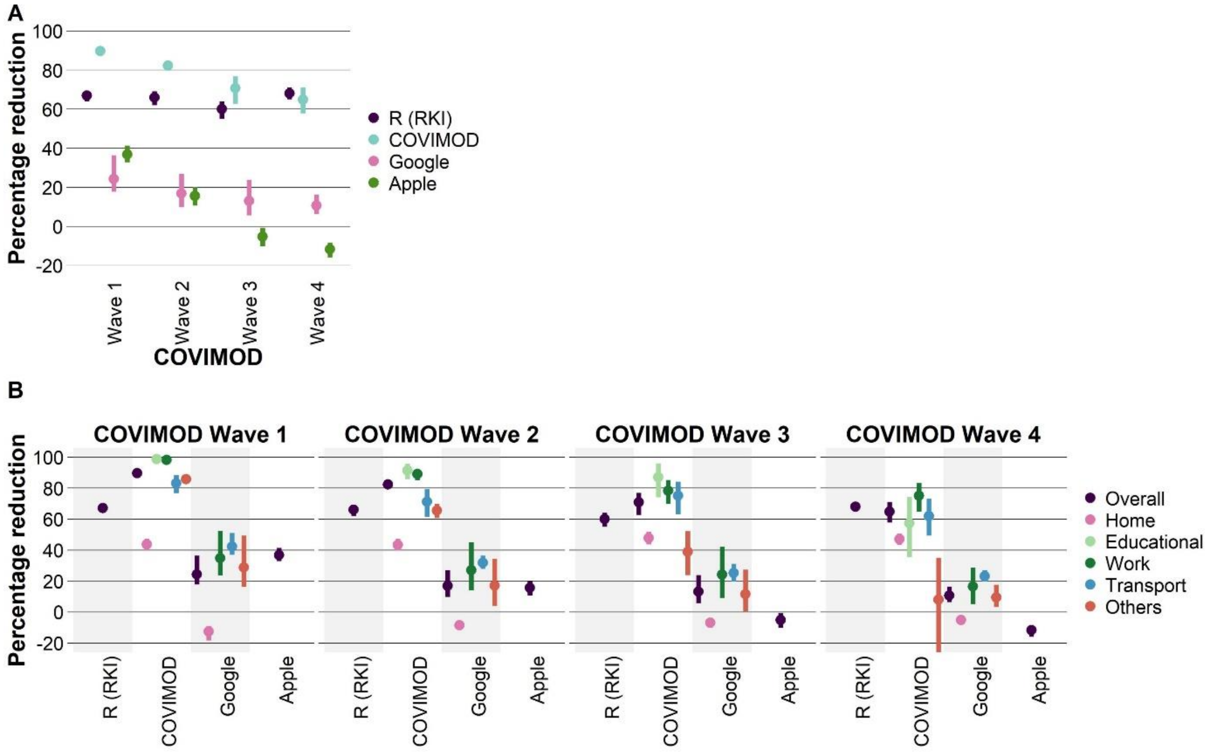

2.6.b. Figure 2.6b Comparison of the percentage reduction in transmission dynamics based on different input data.

Displayed are the mean and bootstrapped 95% confidence interval of the percentage reduction of the R estimates from the RKI and R estimates obtained from COVIMOD (complex approach) compared to the basic reproduction number and the percentage reduction of Google and Apple mobility data (A) displays the overall percentage reduction and (B) displays the percentage reduction by setting for the unweighted analysis including group contacts.

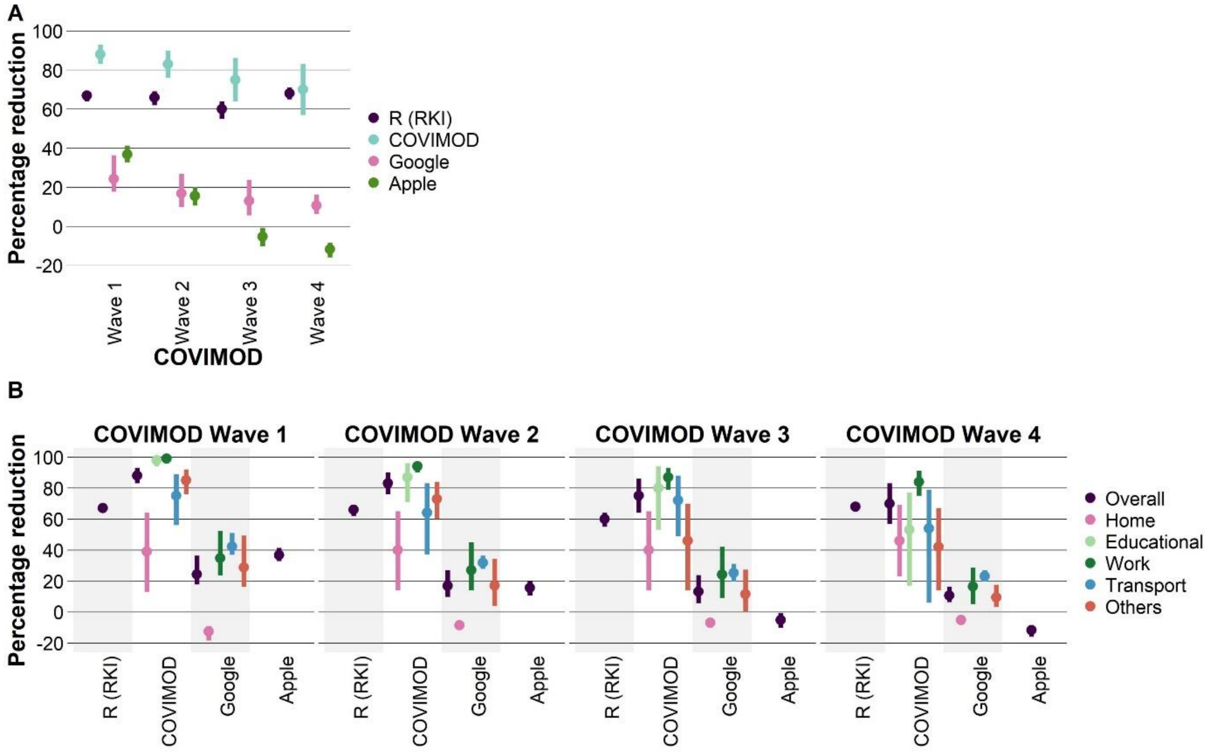

3. **WEIGHTED ANALYSIS WITHOUT GROUP CONTACTS**

3.2. **Number of social contacts**

3.2.a. Table 3.2a Number of recorded overall contacts per participant per day stratified by age, gender, household size, and day of the week in the COVIMOD survey Waves 1 to 4 and the POLYMOD survey for the weighted analysis without group contacts.

	**POLYMOD**		**COVIMOD**							
			**Wave 1**		**Wave 2**		**Wave 3**		**Wave 4**	
	**Mean (SD)**	**Min, Max**	**Mean (SD)**	**Min, Max**	**Mean (SD)**	**Min, Max**	**Mean (SD)**	**Min, Max**	**Mean (SD)**	**Min, Max**
	**8.4 (6.7)**	**1,58**	**2.0 (1.9)**	**0,16**	**3.3 (4.7)**	**0,102**	**3.3 (4.2)**	**0,101**	**3.1 (4.7)**	**0,100**
**Age category**										
0 - 4	9.4 (5.5)	2,30	2.9 (1.6)	0,8	5.0 (2.6)	0,14	4.1 (3.5)	1,14	4.5 (4.0)	0,33
5 - 9	8.4 (5.0)	2,43	3.5 (1.8)	0,8	3.9 (3.3)	0,17	3.4 (2.1)	0,25	3.9 (3.3)	0,31
10 - 14	9.5 (6.0)	1,29	3.2 (1.6)	0,12	3.9 (2.2)	0,9	4.0 (2.4)	0,14	4.8 (4.3)	0,39
15 - 19	10.4 (10.0)	1,50	3.2 (2.0)	0,8	4.4 (3.2)	0,20	3.2 (2.1)	0,14	4.0 (4.1)	0,39
20 - 24	6.1 (3.6)	1,35	2.0 (2.2)	0,16	3.3 (2.7)	0,20	4.6 (8.1)	0,27	3.8 (7.0)	0,84
25 - 34	9.8 (7.0)	1,58	1.7 (1.8)	0,13	3.0 (3.2)	0,18	3.0 (3.4)	0,34	3.7 (6.0)	0,36
35 - 44	9.6 (5.8)	1,38	2.1 (2.0)	0,11	3.3 (2.8)	0,33	3.6 (4.1)	0,18	2.2 (3.5)	0,42
45 - 54	13.1 (8.9)	1,55	1.8 (1.5)	0,9	3.4 (8.2)	0,102	4.4 (5.4)	0,36	2.9 (5.9)	0,100
55 - 64	7.4 (4.8)	1,25	1.8 (1.5)	0,12	3.3 (4.0)	0,42	3.0 (4.1)	0,46	3.2 (3.7)	0,39
65 - 69	7.0 (3.6)	1,40	1.6 (1.7)	0,11	3.4 (6.6)	0,93	3.6 (7.0)	0,101	2.8 (3.6)	0,43
70 - 74	6.0 (4.6)	1,20	1.1 (1.4)	0,8	2.6 (3.1)	0,25	2.8 (2.1)	0,11	2.2 (2.0)	0,16
75 - 79	5.0 (4.0)	1,14	1.5 (1.0)	0,5	2.6 (1.7)	0,9	2.8 (4.1)	0,32	1.9 (1.7)	0,8
80 +	4.2 (2.0)	2,8	0.7 (1.0)	0,3	0.7 (1.9)	0,9	0.2 (0.6)	0,4	0.5 (1.0)	0,4
**Sex of participants**										
Female	8.3 (5.9)	1,45	2 (1.9)	0,16	3.4 (5.5)	0,102	3.6 (4.6)	0,46	3.4 (5.4)	0,100
Male	8.6 (7.2)	1,58	2 (1.8)	0,12	3.2 (3.7)	0,93	3.0 (3.7)	0,101	2.8 (3.8)	0,84
**Household size**										
1	4.9 (5.4)	1,58	0.7 (1.3)	0,12	1.6 (2.4)	0,42	1.5 (2.2)	0,42	2.1 (6.2)	0,100
2	5.8 (4.5)	1,55	1.3 (1.5)	0,11	3.3 (7.6)	0,101	3.2 (5.0)	0,101	2.4 (4.1)	0,84
3	7.5 (5.1)	1,42	2.0 (1.4)	0,11	3.3 (3.5)	0,102	3.5 (4.7)	0,46	3.1 (4.9)	0,43
4 or more	10.8 (7.2)	1,50	3.4 (1.8)	0,16	4.1 (2.9)	0,32	4.5 (3.6)	0,34	4.3 (3.8)	0,39
**Weekdays**										
Monday	7.0 (4.3)	1,31	2.6 (1.7)	0,6	3.6 (3.2)	0,24	3.3 (3.7)	0,42	3.5 (5.0)	0,84
Tuesday	9.0 (8.0)	1,56	2.8 (2.4)	0,8	4.1 (5.2)	0,102	5.5 (6.0)	0,32	4.1 (5.3)	0,43
Wednesday	7.6 (8.3)	1,58	3.3 (3.2)	0,13	3.0 (3.5)	0,42	3.1 (3.6)	0,31	2.8 (3.3)	0,39
Thursday	7.3 (3.2)	1,30	1.9 (1.8)	0,16	3.2 (3.8)	0,93	3.5 (4.8)	0,101	2.8 (4.4)	0,36
Friday	6.1 (5.2)	1,55	2.1 (2.1)	0,8	4.4 (3.9)	0,32	3.6 (5.3)	0,27	3.0 (7.3)	0,100
Saturday	9.5 (6.2)	1,50	1.8 (1.4)	0,8	4.0 (9.8)	0,101	2.8 (2.5)	0,33	3.6 (5.1)	0,35
Sunday	14.4 (9.7)	1,45	1.9 (1.5)	0,12	2.5 (2.6)	0,21	2.3 (2.5)	0,34	2.2 (2.8)	0,43

3.2.b. Table 3.2.b Number of recorded overall contacts per different settings in the COVIMOD survey Waves 1 to 4 and the POLYMOD survey for the weighted analysis without group contacts.

Note: *The displayed educational contacts are based on the group of participants who attended an educational facility (kindergarten, school, university) and work contacts are based on the group of participants who reported to work full-/part-time*

	POLYMOD		COVIMOD							
			Wave 1		Wave 2		Wave 3		Wave 4	
	Mean (SD)	Min, Max	Mean (SD)	Min, Max	Mean (SD)	Min, Max	Mean (SD)	Min, Max	Mean (SD)	Min, Max
Overall	8.4 (6.7)	1,58	2.0 (1.9)	0,16	3.3 (4.7)	0,102	3.3 (4.2)	0,101	3.1 (4.7)	0,100
Home	3.3 (2.3)	0,26	1.6 (1.5)	0,12	1.8 (1.7)	0, 9	1.6 (1.5)	0, 13	1.6 (1.6)	0, 23
Educational	1.8 (3.8)	0,42	0.0 (0.2)	0, 3	0.2 (0.9)	0, 10	0.1 (0.9)	0, 17	0.4 (1.8)	0, 22
Work	2.8 (4.3)	0,56	0.2 (0.9)	0,11	1.5 (6.1)	0,100	1.8 (5.0)	0, 97	1.4 (5.1)	0,100
Transport	0.6 (1.7)	0, 8	0.0 (0.2)	0, 3	0.1 (0.4)	0, 4	0.0 (0.2)	0, 8	0.1 (0.5)	0, 12
Others	3.4 (4.6)	0,45	0.4 (1.0)	0,10	0.9 (1.8)	0, 33	1.0 (2.2)	0, 35	0.9 (2.1)	0, 38

3.3. Comparison of the relative reduction in transmission dynamics

3.3.a. Table 3.3a Comparison of the relative reduction in transmission dynamics based on different input. Given are the mean and bootstrapped 95% confidence interval of the relative percentage mean reduction of the R estimates from the RKI compared to the basic reproduction number and the relative reduction in the number of social contacts from COVIMOD compared to estimates from POLYMOD as well as of mobility estimates compared to pre-pandemic mobility for the weighted analysis without group contact.

	**COVIMOD**	**R (RKI)**	**Google**	**Apple**
	**Relative Mean Percentage Reduction (95%CI)**			
**Overall**				
30th April - 6th May 2020	75.81 (70.72,80.90)	67.00 (69.00,64.00)	24.31 (36.37,17.66)	36.77 (41.31, 32.79)
14th May - 21st May 2020	60.72 (50.99,70.01)	66.00 (69.00,62.00)	16.85 (26.87, 9.80)	15.65 (19.90, 10.67)
28th May - 4th June 2020	60.33 (49.98,69.69)	60.00 (64.00,55.00)	13.05 (23.80, 5.65)	-5.18 (-0.74,-10.19)
11th June - 22nd June 2020	62.53 (53.76,70.48)	68.00 (71.00,65.00)	10.63 (16.25, 6.28)	-11.65 (-8.31,-15.99)
**Home**				
30th April - 6th May 2020	52.76 (44.30,59.42)	-	-12.71 (-9.14,-18.42)	-
14th May - 21st May 2020	46.24 (35.89,55.22)	-	-8.62 (-6.00,-11.62)	-
28th May - 4th June 2020	49.86 (39.57,58.73)	-	-6.88 (-3.88,-10.12)	-
11th June - 22nd June 2020	52.46 (44.23,59.82)	-	-5.25 (-3.17, -7.91)	-
**Educational**				
30th April - 6th May 2020	98.08 (94.29,99.82)	-	-	-
14th May - 21st May 2020	86.86 (68.66,96.68)	-	-	-
28th May - 4th June 2020	93.44 (79.60,99.08)	-	-	-
11th June - 22nd June 2020	73.13 (40.84,89.58)	-	-	-
**Work**				
30th April - 6th May 2020	91.29 (84.71,95.54)	-	34.71 (52.42,23.58)	-
14th May - 21st May 2020	46.90 (11.32,70.40)	-	27.00 (45.10,13.87)	-
28th May - 4th June 2020	34.78 (-15.03,70.20)	-	24.12 (42.00, 9.00)	-
11th June - 22nd June 2020	50.74 (16.82,71.91)	-	16.50 (28.50, 5.17)	-
**Transport**				
30th April - 6th May 2020	91.43 (76.30,97.84)	-	42.14 (50.85,36.86)	-
14th May - 21st May 2020	75.61 (25.49,94.44)	-	31.75 (36.38,27.88)	-
28th May - 4th June 2020	91.45 (74.81,98.10)	-	25.12 (31.12,19.63)	-
11th June - 22nd June 2020	72.83 (20.80,93.26)	-	23.00 (27.00,19.67)	-
**Others**				
30th April - 6th May 2020	86.65 (79.68,91.55)	-	28.71 (49.35,16.14)	-
14th May - 21st May 2020	71.42 (57.99,81.92)	-	17.06 (34.31, 3.94)	-
28th May - 4th June 2020	69.46 (52.03,82.54)	-	11.44 (27.37, 0.32)	-
11th June - 22nd June 2020	71.83 (58.62,81.48)	-	9.46 (17.50, 3.09)	-

3.3.b. Table 3.3b Effective reproduction number at the timing of COVIMOD survey Waves 1 to 4, estimated effective reproduction number based on the reduction of social contacts at the times of COVIMOD survey Waves 1 to 4 assuming values of the basic reproduction number of Norm (2.6, SD=0.54) and the reduction in mobility at the times of the COVIMOD survey Waves, with 10000 bootstrapped samples in various settings and in different time frames for the weighted analysis without group contacts.

	COVIMOD		R (RKI)	
	**Mean reproduction number (95%CI)**	**Percent Mean Reduction Number (95%CI)**	**Mean reproduction number (95%CI)**	**Percent Mean Reduction Number (95%CI)**
**Overall**				
30th April - 6th May 2020	0.57 (0.31,0.89)	79.00 (89.00,66.00)	0.88 (0.82,0.94)	67.00 (69.00,64.00)
14th May - 21st May 2020	0.83 (0.45,1.28)	69.00 (83.00,51.00)	0.91 (0.81,1.00)	66.00 (69.00,62.00)
28th May - 4th June 2020	0.88 (0.46,1.43)	67.00 (83.00,46.00)	1.06 (0.94,1.19)	60.00 (64.00,55.00)
11th June - 22nd June 2020	0.81 (0.45,1.25)	69.00 (83.00,52.00)	0.84 (0.76,0.91)	68.00 (71.00,65.00)
**Home**				
30th April - 6th May 2020	1.40 (0.82,2.01)	47.00 (69.00,23.00)	-	-
14th May - 21st May 2020	1.38 (0.80,2.00)	47.00 (70.00,24.00)	-	-
28th May - 4th June 2020	1.45 (0.83,2.13)	45.00 (69.00,19.00)	-	-
11th June - 22nd June 2020	1.27 (0.75,1.84)	52.00 (72.00,30.00)	-	-
**Educational**				
30th April - 6th May 2020	0.05 (0.01,0.16)	99.00 (100.00,94.00)	-	-
14th May - 21st May 2020	0.26 (0.04,0.92)	90.00 (99.00,65.00)	-	-
28th May - 4th June 2020	0.15 (0.03,0.49)	95.00 (99.00,82.00)	-	-
11th June - 22nd June 2020	0.62 (0.15,1.90)	77.00 (95.00,27.00)	-	-
**Work**				
30th April - 6th May 2020	0.24 (0.09,0.53)	91.00 (97.00, 80.00)	-	-
14th May - 21st May 2020	0.82 (0.36,1.62)	69.00 (87.00, 38.00)	-	-
28th May - 4th June 2020	1.22 (0.36,2.87)	54.00 (87.00,-10.00)	-	-
11th June - 22nd June 2020	0.80 (0.34,1.60)	70.00 (87.00, 39.00)	-	-
**Transport**				

	COVIMOD		R (RKI)	
	**Mean reproduction number (95%CI)**	**Percent Mean Reduction Number (95%CI)**	**Mean reproduction number (95%CI)**	**Percent Mean Reduction Number (95%CI)**
30th April - 6th May 2020	0.80 (0.12,2.97)	70.00 (96.00, -14.00)	-	-
14th May - 21st May 2020	1.13 (0.16,4.27)	57.00 (94.00, -64.00)	-	-
28th May - 4th June 2020	0.73 (0.12,2.69)	72.00 (96.00, -3.00)	-	-
11th June - 22nd June 2020	1.46 (0.18,6.43)	44.00 (94.00,-147.00)	-	-
**Others**				
30th April - 6th May 2020	0.29 (0.13,0.56)	89.00 (95.00,79.00)	-	-
14th May - 21st May 2020	0.59 (0.27,1.09)	78.00 (90.00,59.00)	-	-
28th May - 4th June 2020	0.73 (0.30,1.48)	72.00 (89.00,44.00)	-	-
11th June - 22nd June 2020	0.59 (0.27,1.10)	78.00 (90.00,58.00)	-	-

3.4. Boxplots of the number of overall contacts in various settings

Boxes represent the 25th, 50th and 75th percentiles, the whiskers represent the 10th and 90th percentile and the white dots represent the mean. Participants with no contacts are displayed as 0 on the log-scale of the y-axis.

3.4.a. Figure 3.4a Displayed are the number of social contacts by setting: (a) the overall number of contacts, (b) home contacts, (c) educational contacts, (d) work contacts, (e) public transport contacts and (f) other contacts for the weighted analysis without group contacts in overall contacts.

Note: *The displayed educational contacts are based on the group of participants who attended an educational facility (kindergarten, school, university) and work contacts are based on the group of participants who reported to work full-/part-time*

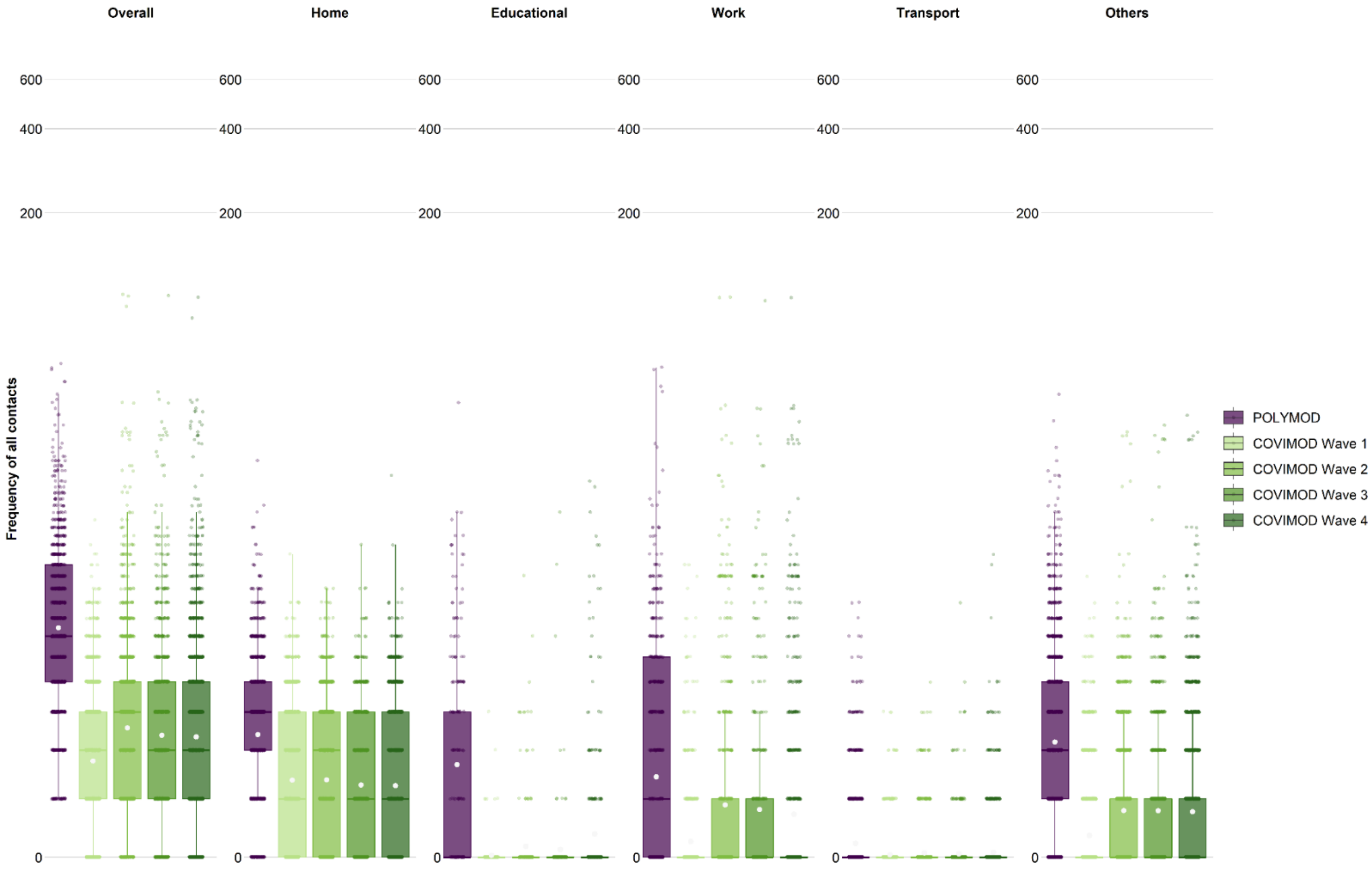

3.4.b. Figure 3.4b Displayed are the number of social contacts stratified by age group: (a) 0 - 7 years, (b) 8 – 15 years, (c) 16 – 23 years, (d) 24 – 38 years, (e) 39 – 60 years and (f) 61 years or more for the weighted analysis without group contacts in overall contacts.

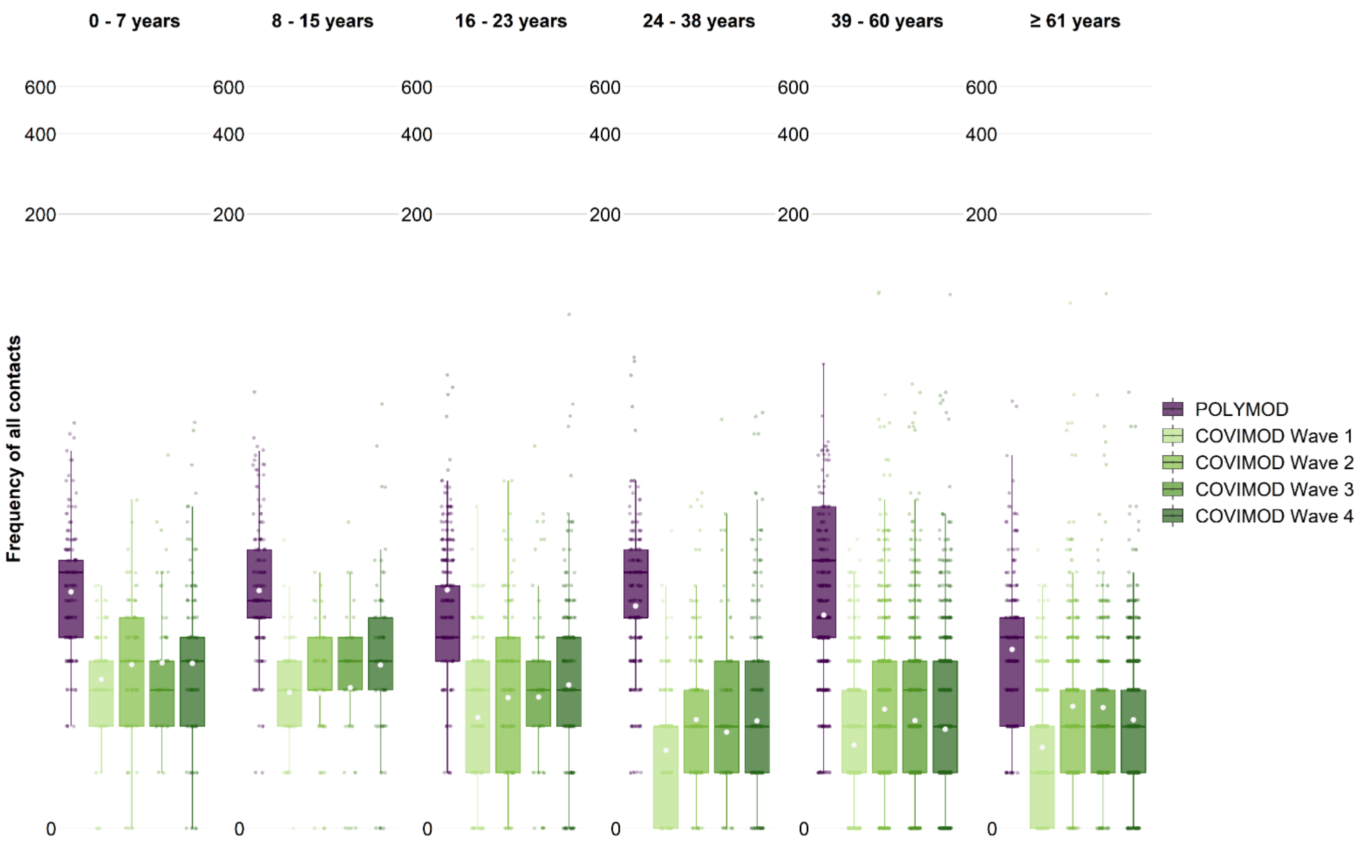

3.4.c. Figure 3.4c Displayed are the number of social contacts stratified by household size: (a) Household size of 1, (b) Household size of 2, (c) Household size of 3 and (d) Household size of 4 or more for the weighted analysis without group contacts in overall contacts.

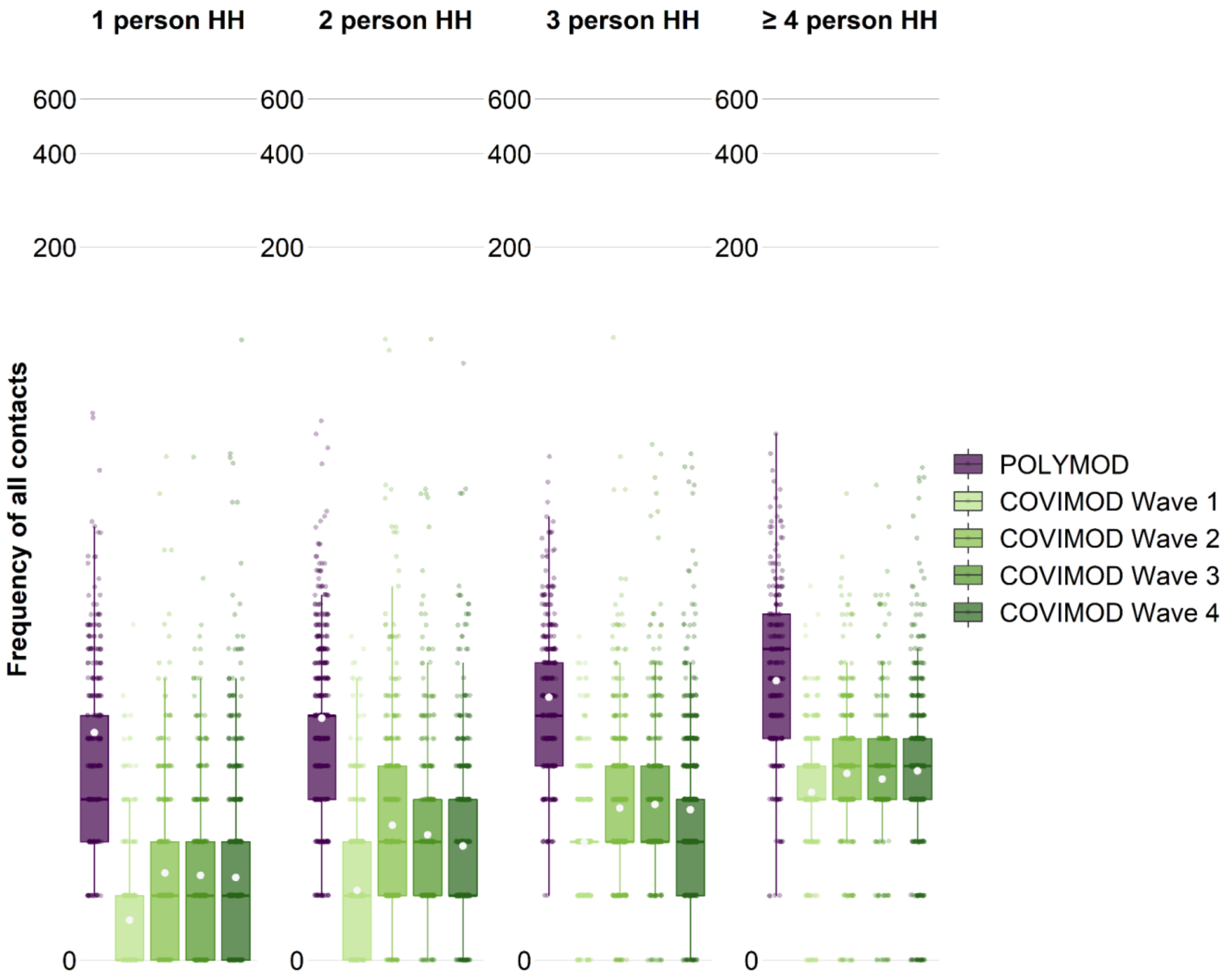

3.4.d. Figure 3.4d Displayed are the number of social contacts stratified by sex (a) Female and (b) Male for the weighted analysis without group contacts in overall contacts.

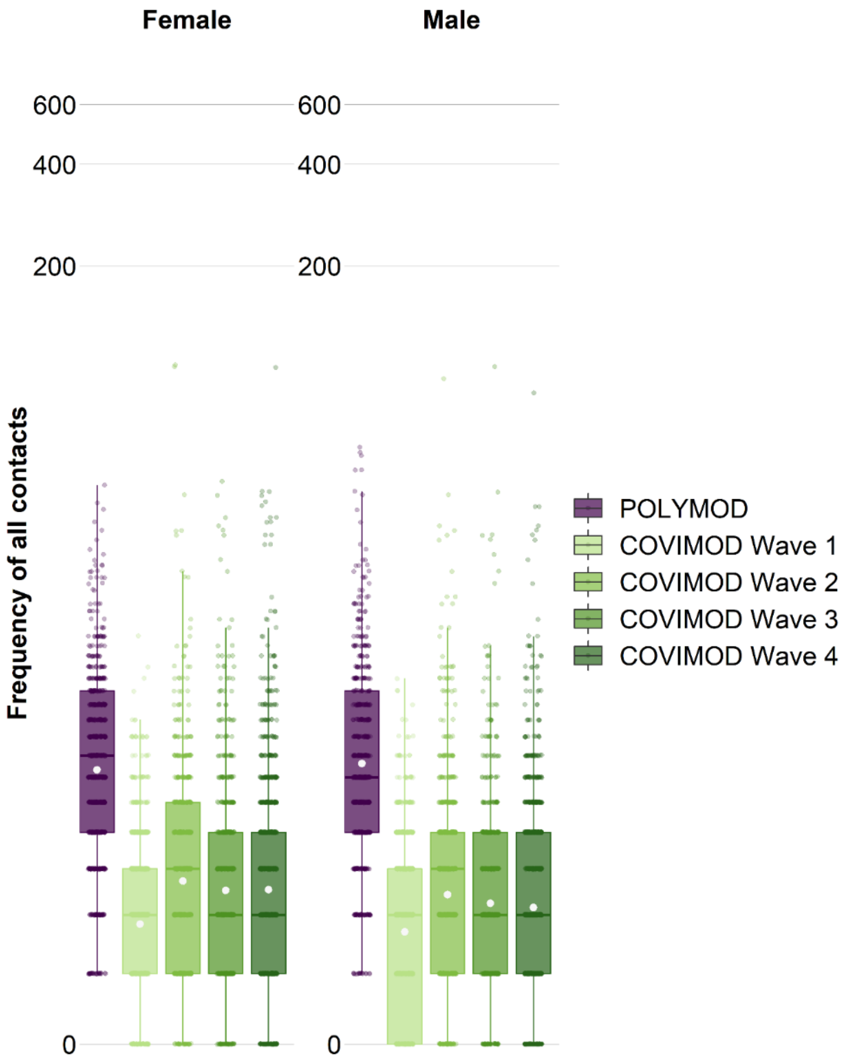

3.4.e. Figure 3.4e Displayed are the number of social contacts stratified by day of the week (a) Monday, (b) Tuesday, (c) Wednesday, (d) Thursday, (e) Friday, (f) Saturday and (g) Sunday for the weighted analysis without group contacts in overall contacts.

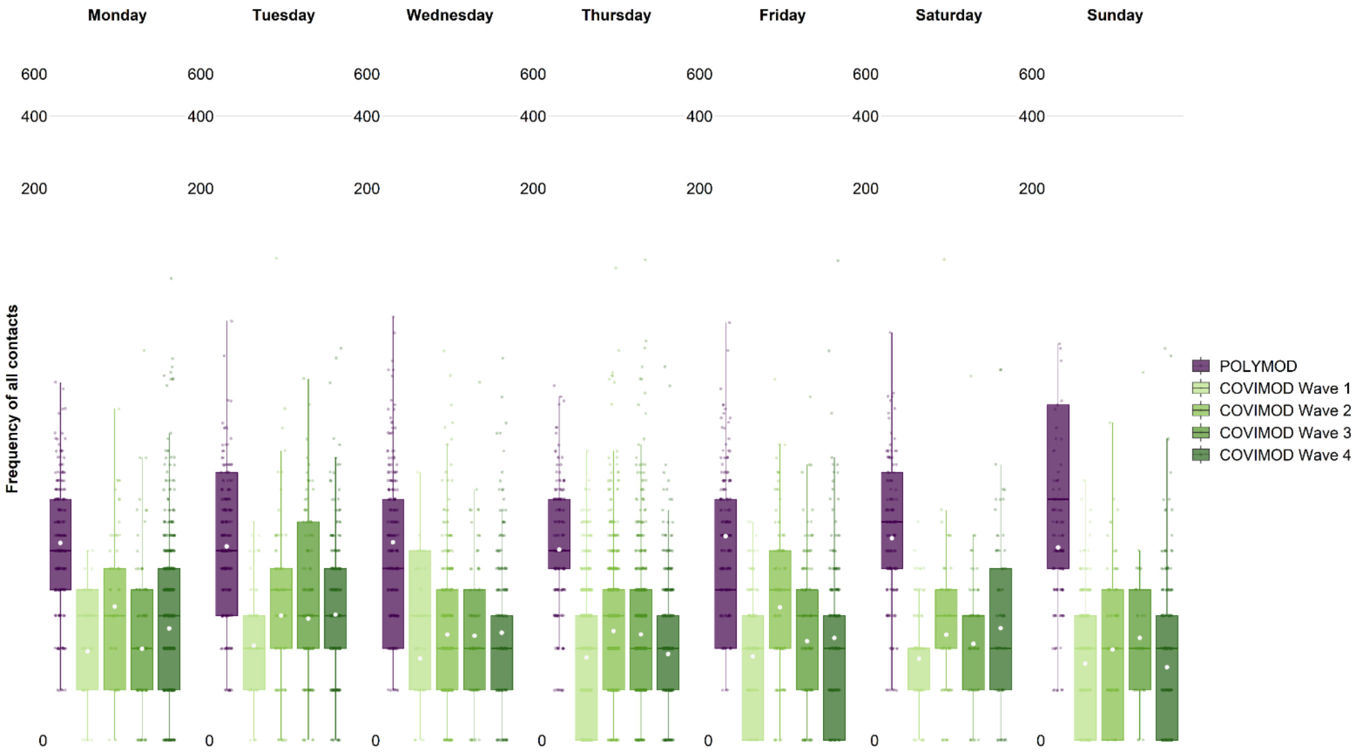

3.5. Social contact patterns

3.5. Figure 3.5 Social contact matrices with the mean total number of reported daily social contacts by participants in different age groups with individuals in other age groups in POLYMOD and COVIMOD survey waves 1 to 4 in various settings. Displayed are (a) the overall number of contacts, (b) home contacts, (c) educational contacts, (d) work contacts, (e) public transport contacts and (f) other contacts for the weighted analysis without group contacts.

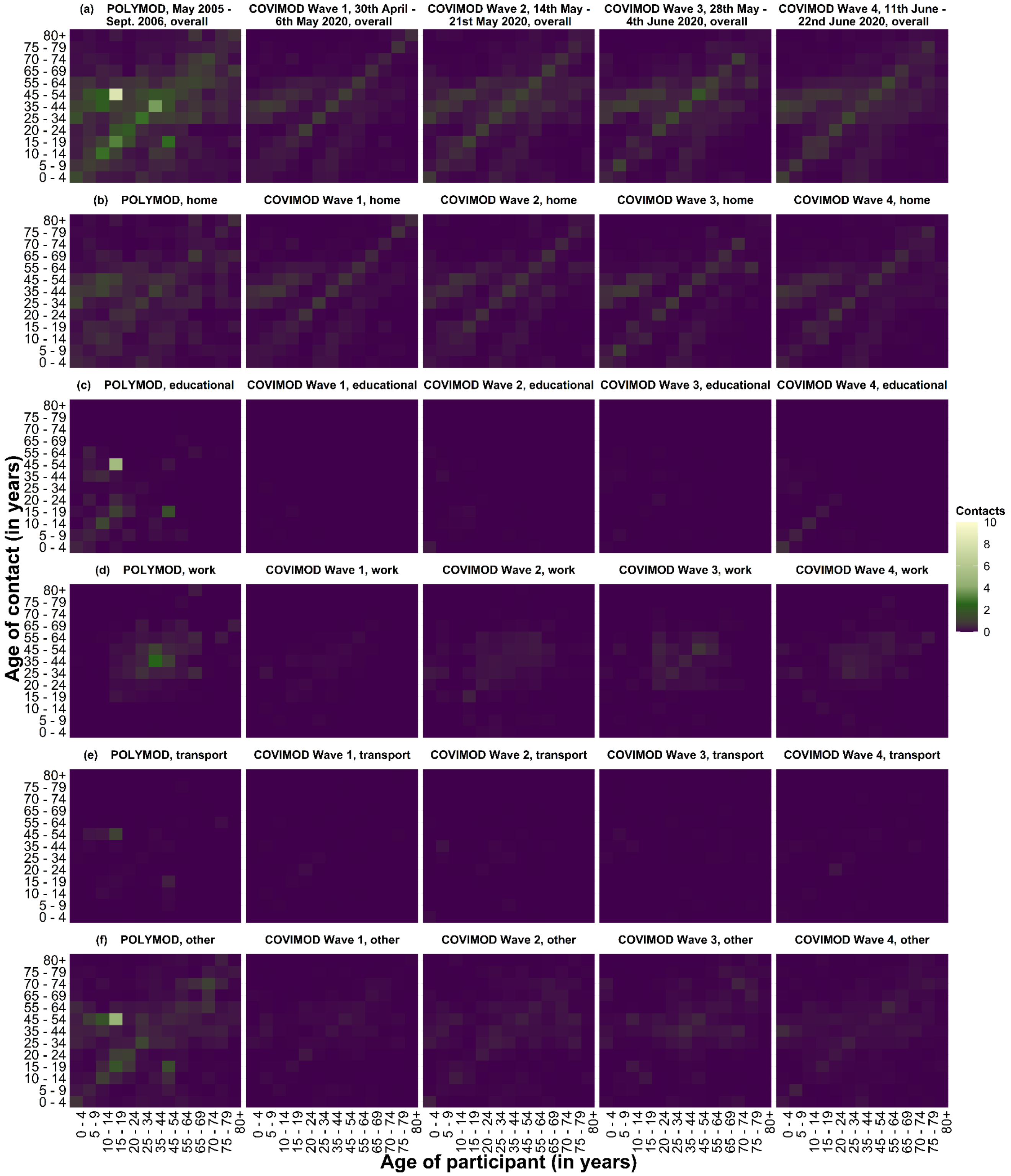

3.6. **Relative reductions in transmission dynamics of SARS-CoV-2**

3.6.a. Figure 3.6a Comparison of the relative reduction in transmission dynamics based on different input data.

Displayed are the mean and bootstrapped 95% confidence interval of the relative reduction of the R estimates from the RKI compared to the basic reproduction number and the relative reduction in the number of social contacts (COVIMOD-simple approach) as well as of mobility estimates compared to pre-pandemic times (A) displays the overall relative reduction and (B) displays the relative reduction by setting for the weighted analysis without group contacts.

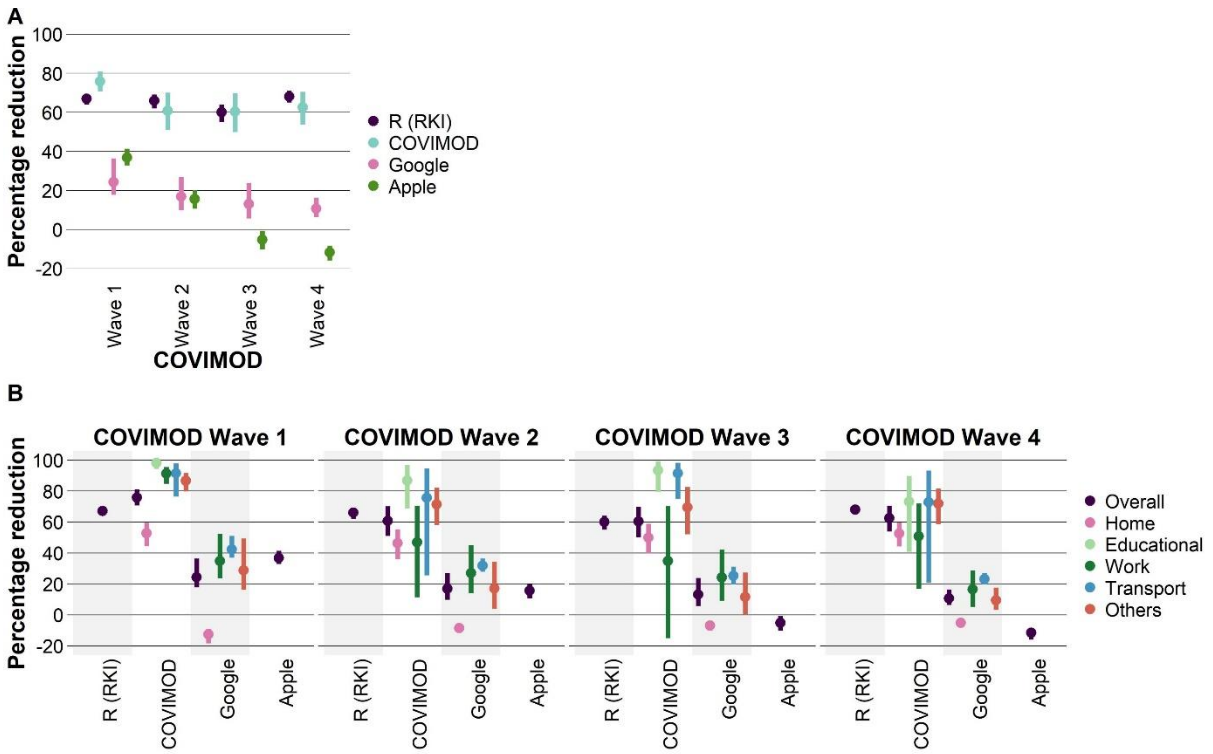

3.6.b. Figure 3.6b Comparison of the percentage reduction in transmission dynamics based on different input data.

Displayed are the mean and bootstrapped 95% confidence interval of the percentage reduction of the R estimates from the RKI and R estimates obtained from COVIMOD (complex approach) compared to the basic reproduction number and the percentage reduction of Google and Apple mobility data (A) displays the overall percentage reduction and (B) displays the percentage reduction by setting for the weighted analysis without group contacts.

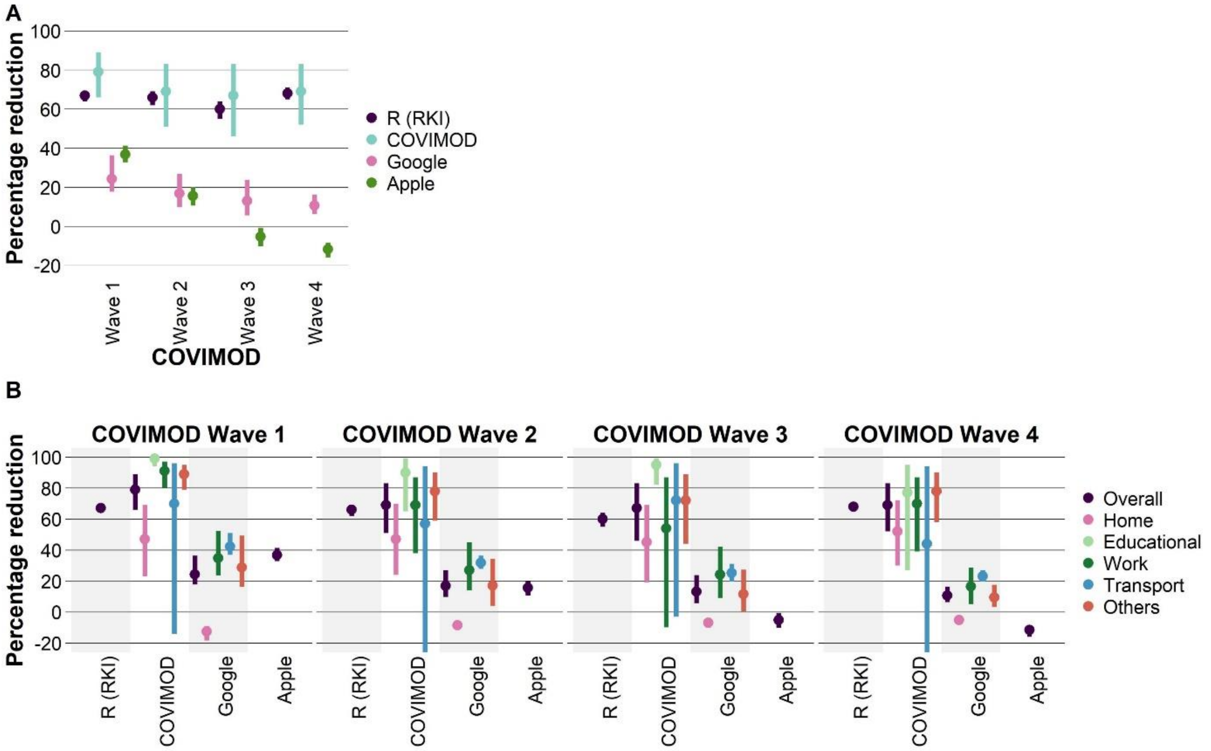

4. **UNWEIGHTED ANALYSIS WITHOUT GROUP CONTACTS**

4.2. **Number of social contacts**

4.2.a. Table 4.2a Number of recorded overall contacts per participant per day stratified by age, gender, household size, and day of the week in the COVIMOD survey Waves 1 to 4 and the POLYMOD survey for the unweighted analysis without group contacts.

	POLYMOD			COVIMOD											
				Wave 1			Wave 2			Wave 3			Wave 4		
	N	Mean (SD)	Min, Max	N	Mean (SD)	Min, Max	N	Mean (SD)	Min, Max	N	Mean (SD)	Min, Max	N	Mean (SD)	Min, Max
	**1341**	**7.9 (6.3)**	**1,58**	**1560**	**2.1 (1.9)**	**0,16**	**1356**	**3.6 (6.1)**	**0,102**	**1081**	**3.4 (5.4)**	**0,101**	**1890**	**3.3 (5.3)**	**0,100**
**Age category**															
0 - 4	89	9.1 (6.1)	2,30	46	3.8 (1.9)	0,8	36	4.7 (2.7)	0,14	21	5.0 (3.7)	1,14	56	4.7 (5.3)	0,33
5 - 9	92	8.4 (5.7)	2,43	48	3.5 (1.7)	0,8	41	4.4 (3.2)	0,17	30	4.8 (4.6)	0,25	62	5.5 (5.1)	0,31
10 - 14	110	9.0 (5.7)	1,29	73	3.2 (1.8)	0,12	63	3.3 (2.0)	0,9	45	3.6 (2.6)	0,14	87	5.1 (5.5)	0,39
15 - 19	121	10.0 (7.6)	1,50	95	3.3 (2.0)	0,8	66	4.1 (3.7)	0,20	45	3.7 (3.1)	0,14	108	4.6 (5.4)	0,39
20 - 24	117	7.9 (4.9)	1,35	83	2.0 (2.6)	0,16	60	3.0 (3.3)	0,20	28	4.1 (6.0)	0,27	109	4.6 (8.9)	0,84
25 - 34	132	9.0 (8.5)	1,58	173	1.7 (1.8)	0,13	148	2.8 (3.2)	0,18	96	2.7 (4.3)	0,34	219	3.3 (5.3)	0,36
35 - 44	156	8.1 (5.4)	1,38	137	1.9 (2.2)	0,11	124	3.0 (3.7)	0,33	91	2.5 (3.0)	0,18	164	2.4 (4.0)	0,42
45 - 54	184	7.9 (7.1)	1,55	235	1.8 (1.7)	0,9	209	4.0 (10.3)	0,102	174	2.9 (4.6)	0,36	275	2.9 (7.3)	0,100
55 - 64	160	6.0 (3.8)	1,25	265	1.9 (1.7)	0,12	244	3.7 (5.1)	0,42	237	3.6 (5.7)	0,46	321	3.0 (4.2)	0,39
65 - 69	74	6.3 (6.6)	1,40	270	1.8 (1.7)	0,11	245	3.9 (7.6)	0,93	199	3.8 (8.3)	0,101	313	3.0 (4.1)	0,43
70 - 74	33	5.1 (4.7)	1,20	89	1.8 (1.5)	0,8	73	3.1 (3.5)	0,25	79	3.0 (2.1)	0,11	118	2.4 (2.2)	0,16
75 - 79	14	4.6 (3.7)	1,14	35	1.7 (1.4)	0,5	34	2.6 (2.1)	0,9	29	3.7 (5.9)	0,32	48	2.8 (2.1)	0,8
80 +	13	3.7 (1.8)	2,8	11	1.1 (1.0)	0,3	11	2.0 (2.8)	0,9	7	1.7 (1.5)	0,4	10	1.5 (1.7)	0,4
**Sex of participants**															
Female	722	7.7 (5.5)	1,45	748	2.2 (2.0)	0,16	638	3.9 (6.8)	0,102	536	3.6 (5.0)	0,46	901	3.7 (6.0)	0,100
Male	581	8.3 (7.2)	1,58	806	2.0 (1.8)	0,12	717	3.3 (5.3)	0,93	544	3.2 (5.9)	0,101	985	3.0 (4.6)	0,84
**Household size**															
1	250	6.8 (6.8)	1,58	232	0.8 (1.5)	0,12	256	2.1 (4.2)	0,42	268	1.9 (3.4)	0,42	487	2.2 (6.2)	0,100
2	411	7.3 (6.2)	1,55	412	1.3 (1.6)	0,11	351	3.8 (8.4)	0,101	270	3.4 (7.6)	0,101	439	2.8 (5.4)	0,84

	POLYMOD			COVIMOD											
				Wave 1			Wave 2			Wave 3			Wave 4		
	N	Mean (SD)	Min, Max	N	Mean (SD)	Min, Max	N	Mean (SD)	Min, Max	N	Mean (SD)	Min, Max	N	Mean (SD)	Min, Max
	**1341**	**7.9 (6.3)**	**1,58**	**1560**	**2.1 (1.9)**	**0,16**	**1356**	**3.6 (6.1)**	**0,102**	**1081**	**3.4 (5.4)**	**0,101**	**1890**	**3.3 (5.3)**	**0,100**
3	339	8.2 (5.4)	1,42	514	2.2 (1.5)	0,11	447	3.6 (6.0)	0,102	343	3.8 (5.1)	0,46	544	3.5 (4.5)	0,43
4 or more	341	9.3 (6.5)	1,50	402	3.5 (1.9)	0,16	302	4.5 (3.5)	0,32	200	4.5 (4.1)	0,34	420	4.9 (4.8)	0,39
**Weekdays**															
Monday	227	7.6 (4.9)	1,31	50	2.2 (1.7)	0,6	60	4.3 (4.1)	0,24	128	2.7 (4.3)	0,42	642	3.6 (5.5)	0,84
Tuesday	237	7.7 (5.9)	1,56	63	2.4 (2.0)	0,8	89	5.0 (11.2)	0,102	143	4.0 (4.6)	0,32	246	4.1 (4.9)	0,43
Wednesday	222	8.5 (7.7)	1,58	54	2.3 (2.5)	0,13	293	3.3 (4.4)	0,42	87	3.1 (3.9)	0,31	172	3.6 (4.9)	0,39
Thursday	179	7.3 (4.9)	1,30	914	2.1 (1.9)	0,16	613	3.4 (5.5)	0,93	489	3.6 (6.6)	0,101	320	2.5 (3.4)	0,36
Friday	186	8.5 (6.7)	1,55	144	2.1 (1.8)	0,8	117	4.4 (4.5)	0,32	132	3.0 (3.5)	0,27	196	3.6 (8.1)	0,100
Saturday	152	8.4 (6.5)	1,50	88	2.0 (1.6)	0,8	63	4.2 (12.6)	0,101	66	2.8 (4.2)	0,33	81	3.9 (5.8)	0,35
Sunday	117	7.9 (7.3)	1,45	247	1.9 (1.9)	0,12	121	2.5 (2.8)	0,21	36	3.2 (5.5)	0,34	233	2.2 (4.4)	0,43

4.2.b. Table 4.2.b Number of recorded overall contacts per different settings in the COVIMOD survey Waves 1 to 4 and the POLYMOD survey for the unweighted analysis without group contacts.

Note: *The displayed educational contacts are based on the group of participants who attended an educational facility (kindergarten, school, university) and work contacts are based on the group of participants who reported to work full-/part-time*

	**POLYMOD**			**COVIMOD**											
				**Wave 1**			**Wave 2**			**Wave 3**			**Wave 4**		
	**N**	**Mean (SD)**	**Min, Max**	**N**	**Mean (SD)**	**Min, Max**	**N**	**Mean (SD)**	**Min, Max**	**N**	**Mean (SD)**	**Min, Max**	**N**	**Mean (SD)**	**Min, Max**
Overall	1341	7.9 (6.3)	1,58	1560	2.1 (1.9)	0,16	1356	3.6 (6.1)	0,102	1081	3.4 (5.4)	0,101	1890	3.3 (5.3)	0,100
Home	1341	2.8 (2.3)	0,26	1560	1.6 (1.5)	0,12	1356	1.6 (1.6)	0,9	1081	1.5 (1.5)	0,13	1890	1.5 (1.7)	0,23
Educational	199	2.8 (4.7)	0,42	310	0.0 (0.2)	0,3	247	0.2 (1.0)	0,10	179	0.2 (1.6)	0,17	385	0.7 (2.5)	0,22
Work	715	2.4 (5.2)	0,56	690	0.4 (1.2)	0,11	613	2.0 (7.2)	0,100	476	1.8 (6.3)	0,97	809	1.6 (5.7)	0,100
Transport	1341	0.3 (0.8)	0,8	1560	0.0 (0.3)	0,3	1356	0.1 (0.4)	0,4	1081	0.1 (0.4)	0,8	1890	0.1 (0.6)	0,12
Others	1341	3.0 (3.8)	0,45	1560	0.4 (1.0)	0,10	1356	1.1 (2.4)	0,33	1081	1.1 (2.9)	0,35	1890	1.1 (2.5)	0,38

4.3. Comparison of the relative reduction in transmission dynamics

4.3.a. Table 4.3a Comparison of the relative reduction in transmission dynamics based on different input. Given are the mean and bootstrapped 95% confidence interval of the relative mean percentage reduction of the R estimates from the RKI compared to the basic reproduction number and the relative reduction in the number of social contacts from COVIMOD compared to estimates from POLYMOD as well as of mobility estimates compared to pre-pandemic mobility for the unweighted analysis without group contact.

	COVIMOD	R (RKI)	Google	Apple
	Relative Mean Percentage Reduction (95%CI)			
**Overall**				
30th April - 6th May 2020	73.71 (72.09,75.18)	67.00 (69.00,64.00)	24.31 (36.37,17.66)	36.77 (41.31, 32.77)
14th May - 21st May 2020	55.12 (50.61,59.29)	66.00 (69.00,62.00)	16.85 (26.87, 9.80)	15.64 (19.91, 10.65)
28th May - 4th June 2020	57.56 (52.65,61.83)	60.00 (64.00,55.00)	13.05 (23.80, 5.65)	-5.18 (-0.74,-10.19)
11th June - 22nd June 2020	58.18 (54.38,61.53)	68.00 (71.00,65.00)	10.63 (16.25, 6.28)	-11.66 (-8.32,-15.99)
**Home**				
30th April - 6th May 2020	43.63 (40.03,47.36)	-	-12.71 (-9.14,-18.42)	-
14th May - 21st May 2020	43.41 (39.37,47.27)	-	-8.62 (-6.00,-11.62)	-
28th May - 4th June 2020	47.64 (43.41,51.55)	-	-6.88 (-3.88,-10.12)	-
11th June - 22nd June 2020	46.84 (43.08,50.24)	-	-5.25 (-3.17, -7.91)	-
**Educational**				
30th April - 6th May 2020	98.67 (97.46,99.53)	-	-	-
14th May - 21st May 2020	91.20 (85.43,95.35)	-	-	-
28th May - 4th June 2020	91.36 (81.29,98.28)	-	-	-
11th June - 22nd June 2020	75.22 (62.76,84.68)	-	-	-
**Work**				
30th April - 6th May 2020	85.42 (79.97,89.50)	-	34.71 (52.42,23.58)	-
14th May - 21st May 2020	16.68 (-12.12,40.87)	-	27.00 (45.10,13.88)	-
28th May - 4th June 2020	27.25 (1.12,49.73)	-	24.12 (42.00, 9.00)	-
11th June - 22nd June 2020	35.81 (14.93,53.41)	-	16.50 (28.50, 5.17)	-
**Transport**				
30th April - 6th May 2020	83.16 (76.87,88.39)	-	42.14 (50.85,36.86)	-
14th May - 21st May 2020	70.82 (61.43,78.57)	-	31.75 (36.38,27.88)	-
28th May - 4th June 2020	75.32 (65.63,84.26)	-	25.12 (31.12,19.63)	-
11th June - 22nd June 2020	61.91 (49.49,72.63)	-	23.00 (27.00,19.67)	-
**Others**				
30th April - 6th May 2020	85.78 (83.87,87.57)	-	28.71 (49.35,16.14)	-
14th May - 21st May 2020	65.22 (60.16,69.86)	-	17.06 (34.31, 3.94)	-
28th May - 4th June 2020	62.69 (55.85,68.21)	-	11.44 (27.37, 0.32)	-
11th June - 22nd June 2020	65.16 (59.78,69.25)	-	9.46 (17.50, 3.09)	-

4.3.b. Table 4.3b Effective reproduction number at the timing of COVIMOD survey Waves 1 to 4, estimated effective reproduction number based on the reduction of social contacts at the times of COVIMOD survey Waves 1 to 4 assuming values of the basic reproduction number of Norm (2.6, SD=0.54) and the reduction in mobility at the times of the COVIMOD survey Waves, with 10000 bootstrapped samples in various settings and in different time frames for the unweighted analysis without group contacts

	COVIMOD		R (RKI)	
	**Mean reproduction number (95%CI)**	**Percent Mean Reduction Number (95%CI)**	**Mean reproduction number (95%CI)**	**Percent Mean Reduction Number (95%CI)**
**Overall**				
30th April - 6th May 2020	0.70 (0.42,0.99)	74.00 (84.00,62.00)	0.88 (0.82,0.94)	67.00 (69.00,64.00)
14th May - 21st May 2020	0.99 (0.58,1.41)	62.00 (78.00,46.00)	0.90 (0.81,0.99)	66.00 (69.00,62.00)
28th May - 4th June 2020	0.96 (0.56,1.36)	64.00 (79.00,48.00)	1.06 (0.94,1.19)	60.00 (64.00,55.00)
11th June - 22nd June 2020	1.00 (0.59,1.44)	62.00 (78.00,45.00)	0.84 (0.76,0.91)	68.00 (71.00,65.00)
**Home**				
30th April - 6th May 2020	1.60 (0.96,2.27)	39.00 (64.00,13.00)	-	-
14th May - 21st May 2020	1.58 (0.93,2.26)	40.00 (65.00,14.00)	-	-
28th May - 4th June 2020	1.56 (0.90,2.25)	40.00 (66.00,14.00)	-	-
11th June - 22nd June 2020	1.41 (0.84,2.00)	46.00 (68.00,24.00)	-	-
**Educational**				
30th April - 6th May 2020	0.07 (0.02,0.16)	98.00 (100.00,94.00)	-	-
14th May - 21st May 2020	0.34 (0.12,0.78)	87.00 (96.00,70.00)	-	-
28th May - 4th June 2020	0.42 (0.08,1.19)	84.00 (97.00,55.00)	-	-
11th June - 22nd June 2020	1.10 (0.49,2.01)	58.00 (82.00,23.00)	-	-
**Work**				
30th April - 6th May 2020	0.28 (0.15,0.43)	90.00 (95.00,84.00)	-	-
14th May - 21st May 2020	0.96 (0.52,1.51)	64.00 (80.00,42.00)	-	-
28th May - 4th June 2020	0.96 (0.48,1.62)	64.00 (82.00,38.00)	-	-
11th June - 22nd June 2020	1.03 (0.54,1.72)	61.00 (80.00,34.00)	-	-
**Transport**				

	COVIMOD		R (RKI)	
	**Mean reproduction number (95%CI)**	**Percent Mean Reduction Number (95%CI)**	**Mean reproduction number (95%CI)**	**Percent Mean Reduction Number (95%CI)**
30th April - 6th May 2020	0.64 (0.31,1.14)	76.00 (89.00,57.00)	-	-
14th May - 21st May 2020	0.91 (0.43,1.62)	65.00 (84.00,38.00)	-	-
28th May - 4th June 2020	0.72 (0.33,1.32)	73.00 (88.00,50.00)	-	-
11th June - 22nd June 2020	1.18 (0.54,2.33)	55.00 (80.00,11.00)	-	-
**Others**				
30th April - 6th May 2020	0.40 (0.22,0.65)	85.00 (92.00,75.00)	-	-
14th May - 21st May 2020	0.72 (0.42,1.04)	73.00 (84.00,60.00)	-	-
28th May - 4th June 2020	0.91 (0.48,1.57)	65.00 (82.00,40.00)	-	-
11th June - 22nd June 2020	0.76 (0.44,1.12)	71.00 (84.00,57.00)	-	-

4.4. **Boxplots of the number of overall in various settings**

Boxes represent the 25th, 50th and 75th percentiles, the whiskers represent the 10th and 90th percentile and the white dots represent the mean. Participants with no contacts are displayed as 0 on the log-scale of the y-axis.

4.4.a. Figure 4.4a Displayed are the number of social contacts by setting (a) the overall number of contacts, (b) home contacts, (c) educational contacts, (d) work contacts, (e) public transport contacts and (f) other contacts for the unweighted analysis without group contacts in overall contacts.

Note: *The displayed educational contacts are based on the group of participants who attended an educational facility (kindergarten, school, university) and work contacts are based on the group of participants who reported to work full-/part-time*

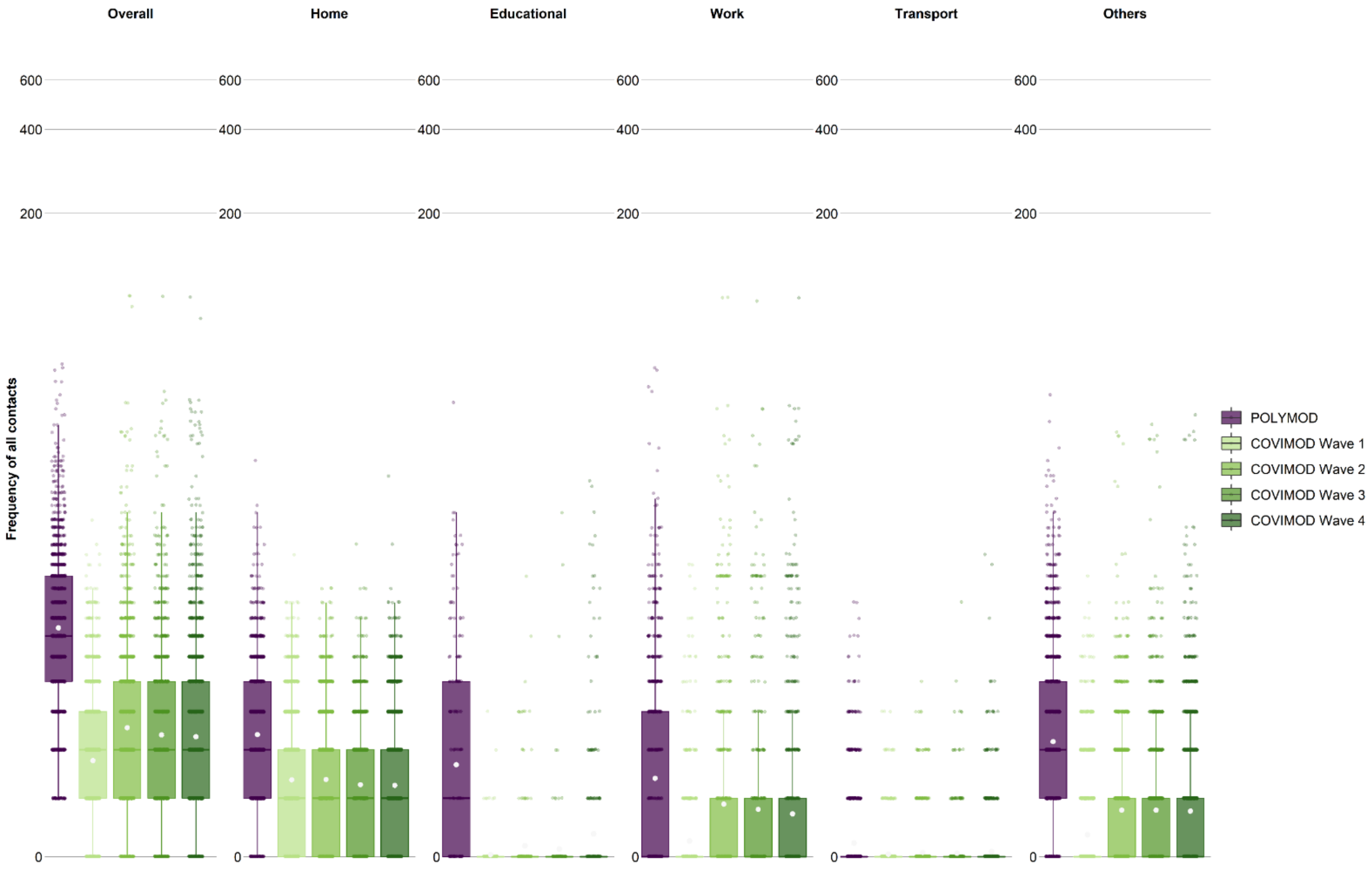

4.4.b. Figure 4.4b Displayed are the number of social contacts stratified by age group (a) 0 - 7 years, (b) 8 – 15 years, (c) 16 – 23 years, (d) 24 – 38 years, (e) 39 – 60 years and (f) 61 years or more for the unweighted analysis without group contacts in overall contacts.

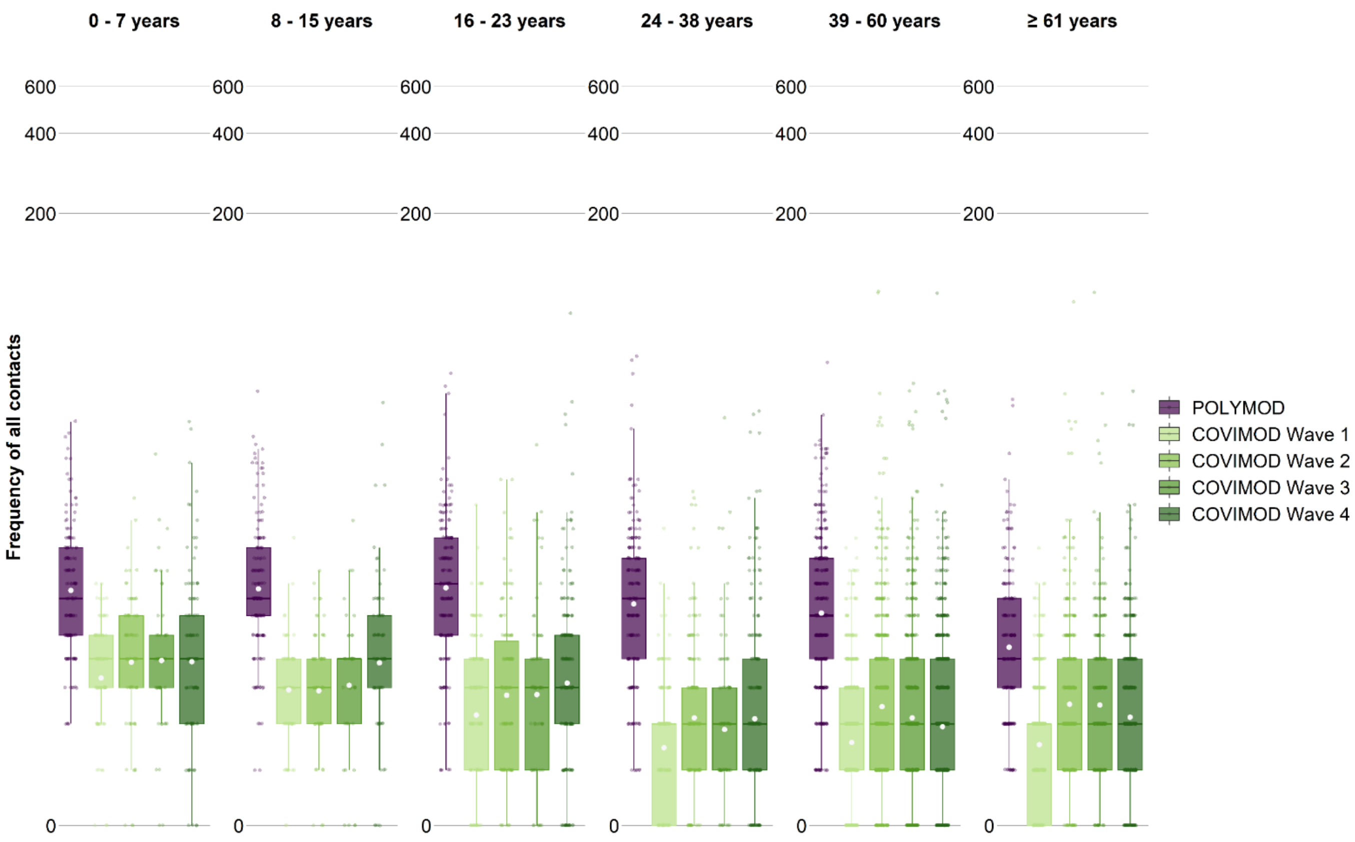

4.4.c. Figure 4.4c Displayed are the number of social contacts stratified by household size (a) Household size of 1, (b) Household size of 2, (c) Household size of 3 and (d) Household size of 4 or more for the unweighted analysis without group contacts in overall contacts.

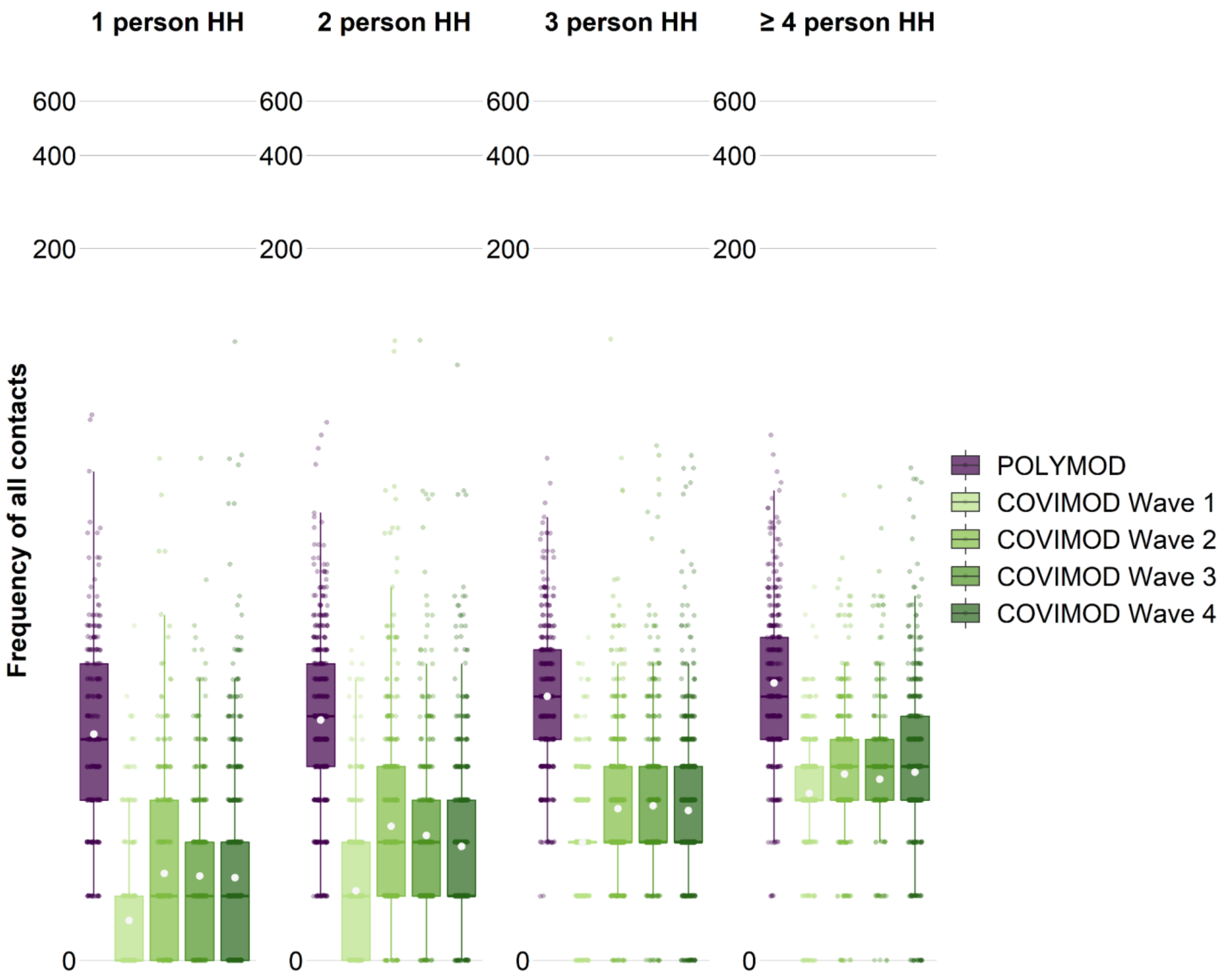

4.4.d. Figure 4.4d Displayed are the number of social contacts stratified by sex (a) Female and (b) Male for the unweighted analysis without group contacts in overall contacts.

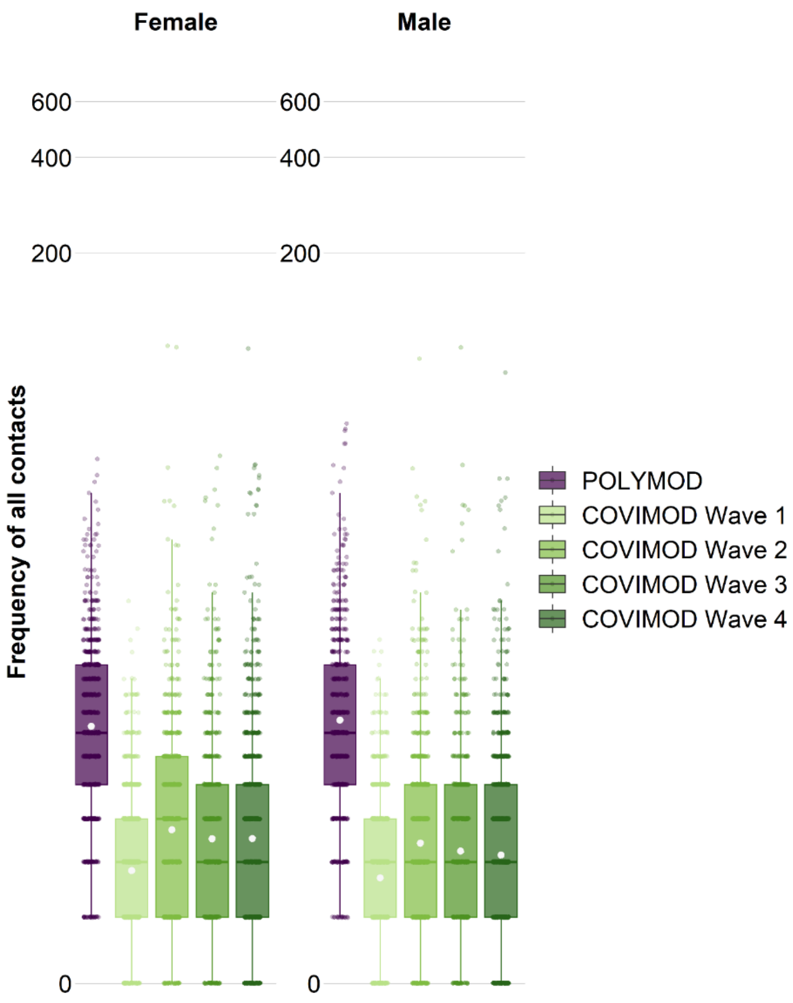

4.4.e. Figure 4.4e Displayed are the number of social contacts stratified by day of the week (a) Monday, (b) Tuesday, (c) Wednesday, (d) Thursday, (e) Friday, (f) Saturday and (g) Sunday for the unweighted analysis without group contacts in overall contacts.

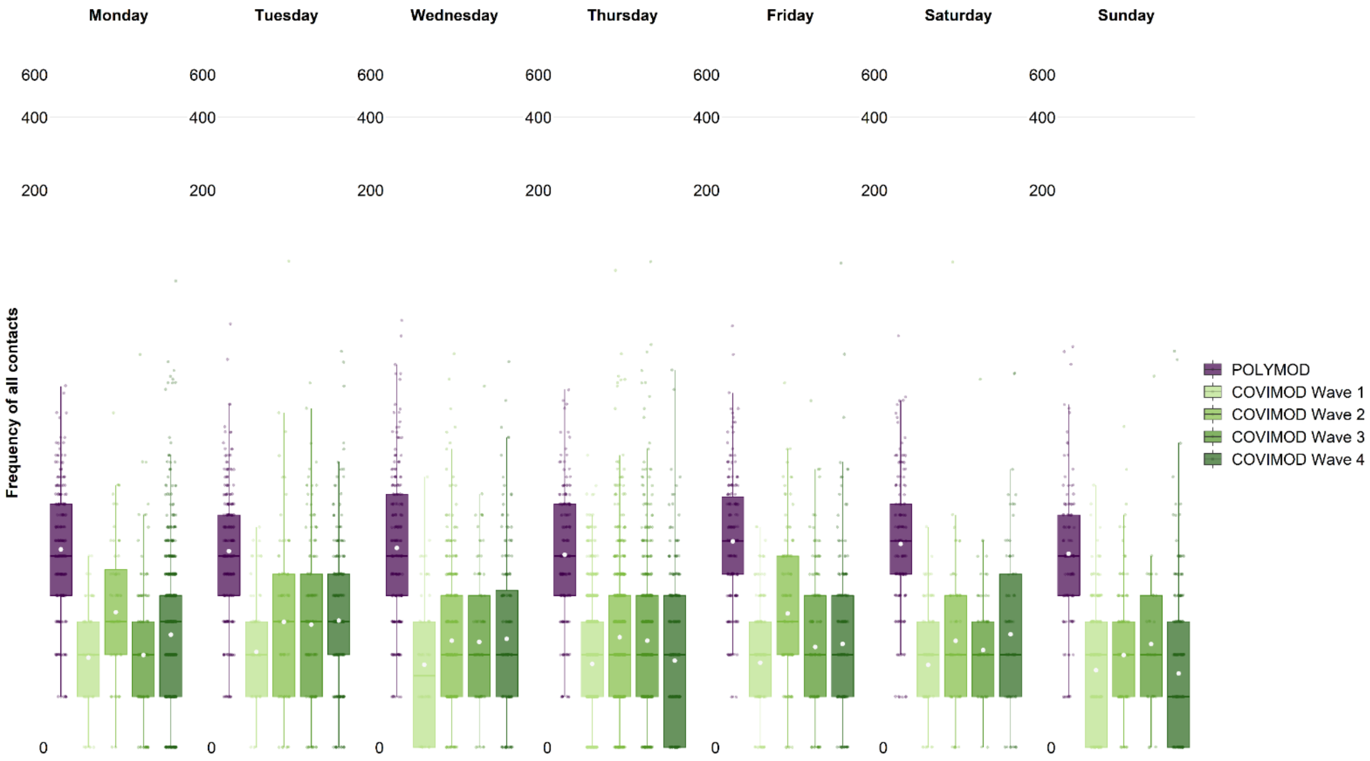

4.5. **Social contact patterns**

4.5. Figure 4.5. Social contact matrices with the mean total number of reported daily social contacts by participants in different age groups with individuals in other age groups in POLYMOD and COVIMOD survey waves 1 to 4 in various settings. Displayed are (a) the overall number of contacts, (b) home contacts, (c) educational contacts, (d) work contacts, (e) public transport contacts and (f) other contacts for the unweighted analysis without group contacts.

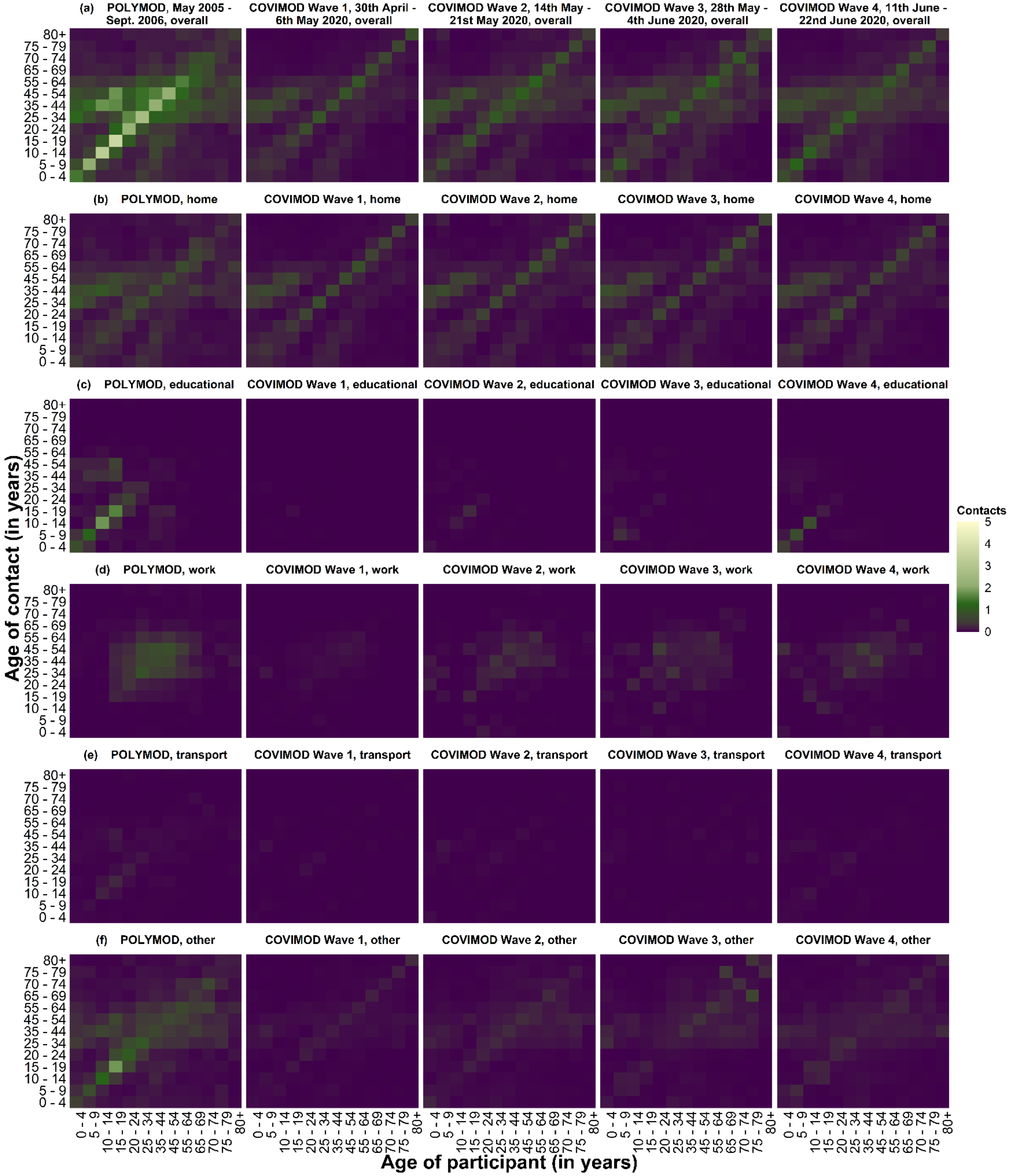

4.6. Relative reductions in transmission dynamics of SARS-CoV-2

4.6.a. Figure 4.6a Comparison of the relative reduction in transmission dynamics based on different input data.

Displayed are the mean and bootstrapped 95% confidence interval of the relative reduction of the R estimates from the RKI compared to the basic reproduction number and the relative reduction in the number of social contacts (COVIMOD-simple approach) as well as of mobility estimates compared to pre-pandemic times (A) displays the overall relative reduction and (B) displays the relative reduction by setting for the unweighted analysis without group contacts.

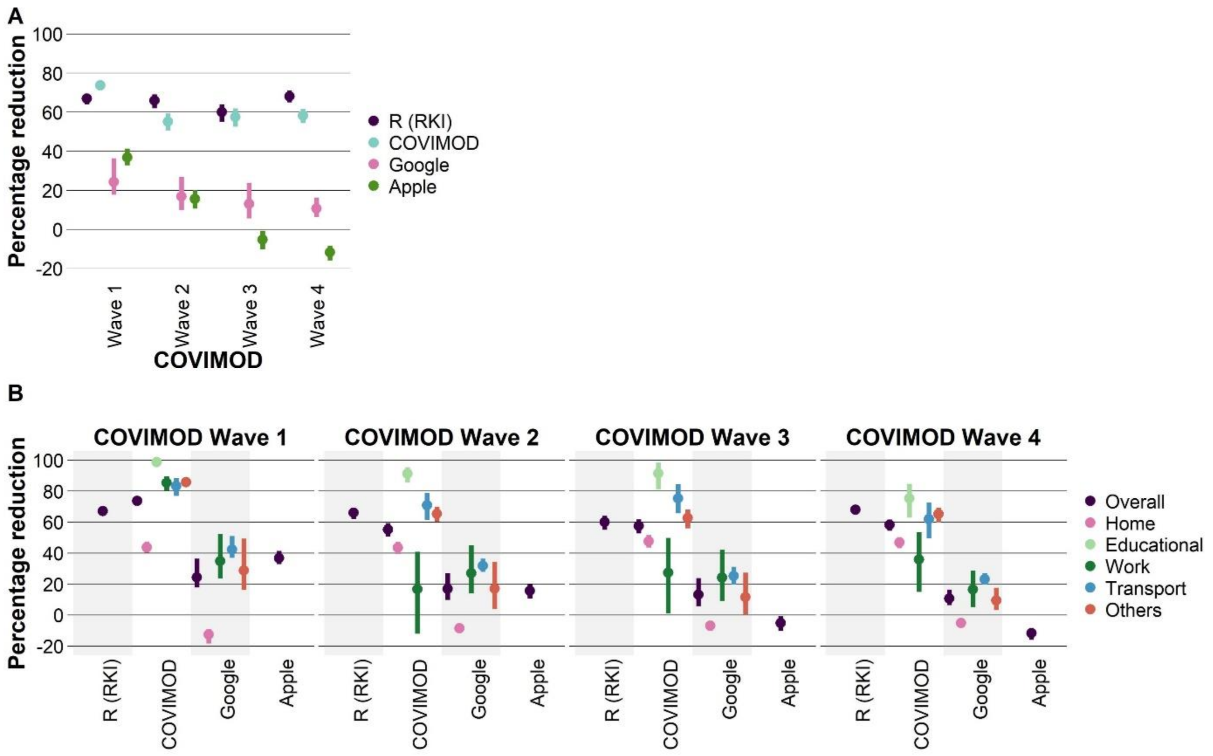

4.6.b. Figure 4.6b Comparison of the percentage reduction in transmission dynamics based on different input data.

Displayed are the mean and bootstrapped 95% confidence interval of the percentage reduction of the R estimates from the RKI and R estimates obtained from COVIMOD (complex approach) compared to the basic reproduction number and the percentage reduction of Google and Apple mobility data (A) displays the overall percentage reduction and (B) displays the percentage reduction by setting for the unweighted analysis without group contacts.

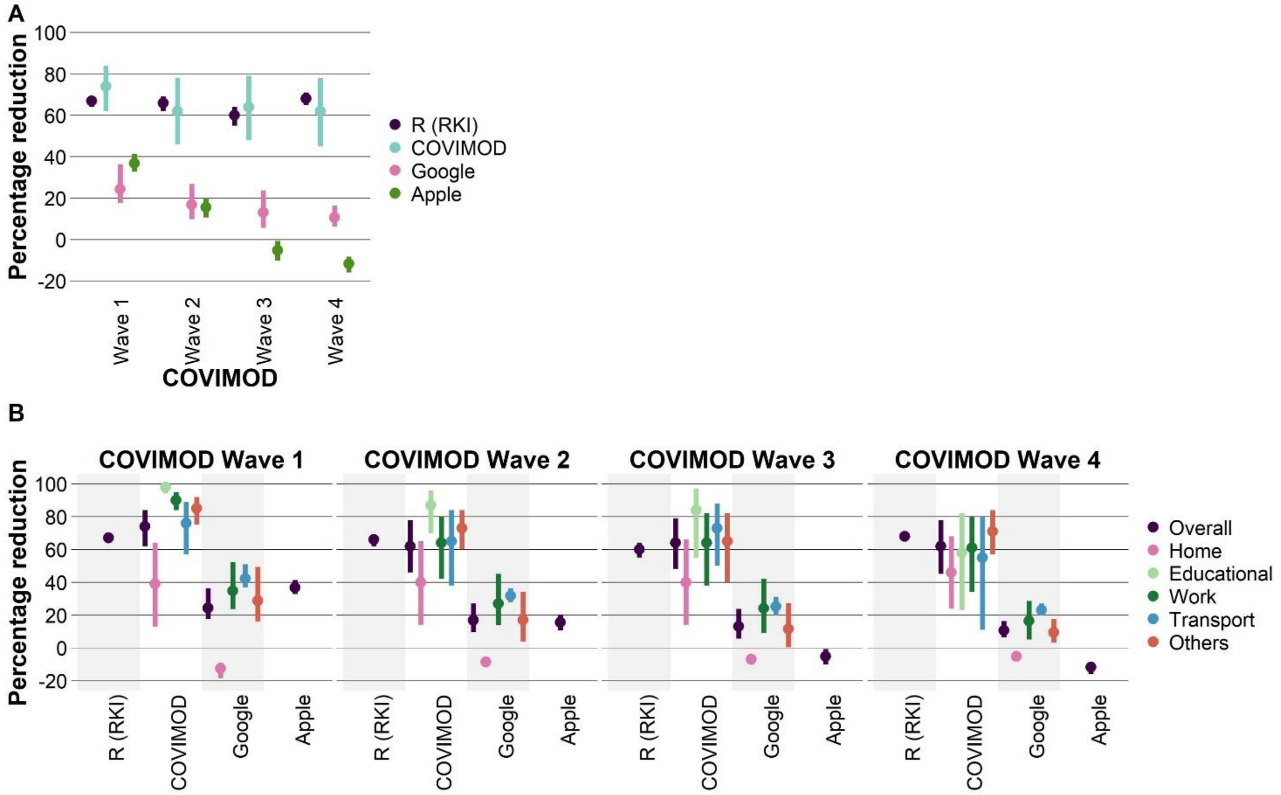

Supplement: Supplementary file 3 — Additional file 3. Additional results. This file provides results additional to the ones provided in the manuscript. [file 12916_2021_2139_MOESM3_ESM.pdf]
