# Supplementary material for: Individual social contact data and population mobility data as early markers of SARS-CoV-2 transmission dynamics during the first wave in Germany—an analysis based on the COVIMOD study
Source: BMC Med. 2021 Oct 14;19:271. doi: 10.1186/s12916-021-02139-6 (PMC8515158; doi:10.1186/s12916-021-02139-6)
Supplement: Supplementary file 4 — Additional file 4. Additional data analyses information. This file provides more data analyses details. [file 12916_2021_2139_MOESM4_ESM.pdf]

Individual social contact data and population mobility data as early markers of SARS-CoV-2 transmission dynamics during the first wave in Germany – an analysis based on the COVIMOD study

Additional File 4: Additional data analyses information

Damilola Victoria Tomori[1], Nicole Rübsamen[1], Tom Berger[1], Stefan Scholz[2], Jasmin Walde[1], Ian Wittenberg[3], Berit Lange[4,5], Alexander Kuhlmann[6,7], Johannes Horn[3], Rafael Mikolajczyk[3], Veronika K Jaeger[1*], André Karch[1*]

[1]Institute of Epidemiology and Social Medicine, University of Münster, Münster, Germany
[2]Immunization Unit, Robert Koch-Institute, Berlin, Germany
[3]Institute for Medical Epidemiology, Biostatistics and Informatics, University of Halle, Halle, Germany
[4]Department of Epidemiology, Helmholtz Centre for Infection Research, Braunschweig, Germany
[5]German Center for Infection Research, Hannover-Braunschweig site, Germany
[6]Center for Health Economics Research Hannover (CHERH), Leibniz Universität Hannover, Hanover, Germany
[7]Biomedical Research in End-Stage and Obstructive Lung Disease Hannover (BREATH), German Center for Lung Research (DZL), Hanover, Germany

Corresponding author
Veronika K. Jaeger, PhD
Institute of Epidemiology and Social Medicine
University of Münster
Domagkstraße 3
48149 Münster
veronika.jaeger@ukmuenster.de

1 Reproduction number of the German Public Health Institute (Robert Koch Institute (RKI))

The method applied by the RKI to obtain R values is based on the reported numbers of individuals notified for being newly infected with SARS-CoV-2. Incident cases are attributed to the day of the first symptoms. If this information is not available, it is imputed taking into account the age of the case, the day and week of the notification, and the estimated delays from the day of the first symptom to the notification date of the cases. The estimation of the notification delay was based on cases for which all information was available; changes in these delays over the course of the pandemic are taken into account [1–3].

As for recent SARS-CoV-2 cases, only those with a short time period between a positive test and the notification are reported, a nowcasting approach is applied [4]. The nowcasting produces an estimate of the additional number of SARS-CoV-2 cases that have already occurred but have not yet been identified by the surveillance system in Germany, taking into account the delay in diagnosis, reporting and transmission. For this purpose, the proportion of cases that were reported after a certain number of days, x, after the onset of the disease, were determined. This proportion is then used to correct the number of cases submitted with the onset of illness x days before the analysis [1–3].

The imputation and the nowcasting lead to an estimated epidemic curve based on which the time-dependent reproduction number (R) can be estimated. With an assumed constant generation time of 4 days, R is defined as the quotient of the number of new infections in two consecutive time frames of 4 days each.

$$\mathrm{R} = \frac{\bar{E}_t^4}{\bar{\mathrm{E}}_{t-4}^4}$$

where $\bar{E}_t^4$ is the sliding average of the number of infected cases over 4 days [1, 2].

In this study, the 4-day reproduction number calculated by the RKI was used. This provides information on the transmission dynamics 8 to 13 days prior [1–3]. The R values are continuously corrected by the RKI retrospectively for delayed notifications. As we extracted the estimated R values over a year after the day they were calculated for, all delayed notifications were already accounted for in the estimates used in this study.

2 Estimating the relative reduction in contacts (COVIMOD), population mobility (Google and Apple) and the reproduction number obtained from the RKI

NOTE: We assumed R0 pre-pandemically to follow a normal distribution with mean 2.6 and standard deviation 0.54 [5].

Google and Apple data provided were compared to pre-pandemic times. The baseline for Google is the median value of the corresponding day of the week during the 5-week period January 3 to February 6, 2020. Apple base volume is January 13, 2020 [6, 7].

The below analysis produced Figure 2 and Figure 4a in the manuscript. The same analysis was performed for Figure 3, where we considered a relative reduction of mobility/contacts in Google and COVIMOD for different settings (i.e. home, school, work, transport, and others).

STEP 1 - weighing

For COVIMOD and POLYMOD we assigned survey weights based on participants' age, sex, household size, day of the week the questionnaire was filled in for and region of residence. We weighted the population mobility data (Google and Apple) and our reference reproduction number (RKI) by the day of the week only.

STEP 2 – weighted means

We generated the weighted relative reduction in population mobility for Google and Apple, $\bar{X}_{Google}\ and\ \bar{X}_{Apple}$, the weighted mean reproduction number, $\bar{X}_{RKI}$, the weighted mean of the reported number of contacts during the SARS-CoV-2 pandemic (COVIMOD - simple approach), $\bar{X}_{COVIMOD\ simple}$, and the weighted mean number of contacts before the pandemic (POLYMOD) $\bar{X}_{POLYMOD}$.

From the COVIMOD data, we also estimated the mean reproduction number from the eigenvalue obtained from the symmetric next generation matrix by assuming R0 pre-pandemically to follow a normal distribution with mean 2.6 and standard deviation 0.54 and taking the survey weights into account (COVIMOD – complex approach; $\bar{X}_{COVIMOD\ complex}$).

STEP 3 – relative reductions in transmission dynamics

Reproduction number (RKI):

We estimated the relative reduction in the reproduction number from the basic reproduction number for the reproduction number estimated by the RKI by assuming R0 to follow a normal distribution with mean 2.6. That is,

$$\text{RKI}_{\text{ relative reduction}} = \frac{(2.6 - \bar{X}_{RKI})}{2.6} * 100$$

Population mobility:

As both Google and Apple mobility data already describe the relative reduction in mobility compared to mobility before the SARS-CoV-2 pandemic in Germany, the weighted means obtained above are describing the relative reduction.

$$\text{Google}_{\text{ relative reduction}} = \bar{X}_{Google}$$

$$\text{Apple}_{\text{ relative reduction}} = \bar{X}_{Apple}$$

COVIMOD simple approach:

We estimated the relative reduction in contacts during the pandemic compared to the pre-pandemic using the weighted mean reported number of contacts from COVIMOD and using the weighted mean reported number of contacts from the POLYMOD data as a baseline.

$$\text{COVIMOD simple approach}_{\text{relative reduction}} = \frac{(\bar{X}_{POLYMOD} - \bar{X}_{COVIMOD\ simple}) * 100}{\bar{X}_{POLYMOD}}$$

COVIMOD complex approach:

For the complex approach we used the estimated reproduction number based on the COVIMOD data and calculated the relative reduction compared to the basic reproduction number:

$$\text{COVIMOD complex approach}_{\text{relative reduction}} = \frac{(2.6 - \bar{X}_{COVIMOD\ complex})}{2.6} * 100$$

We used the package *boot* to compute 95% confidence intervals around the weighted mean of the relative reductions (1000 samples) [8].

3 Relative reduction in transmission dynamics with a scaling factor but without separate weighing for home/non-home contacts

The below analysis produced Figure 4b in the manuscript

The following estimates obtained under (2) are used as a base: $\text{RKI}_{\text{relative reduction}}$, COVIMOD simple $\text{approach}_{\text{relative reduction}}$, COVIMOD complex $\text{approach}_{\text{relative reduction}}$, $\text{Google}_{\text{relative reduction}}$, $\text{Apple}_{\text{relative reduction}}$.

STEP 1

We performed an analysis in which we fitted a scaling factor with the same weight for all types of contacts and mobility. For this, we minimised the residual sum of squares across the four survey waves using the *optim* function in R, i.e.:

$$\sum_{wave=1}^{4} (\delta * DATA_{wave} - RKI_{relative\ reduction;\ wave})^2$$

Where δ is the scaling factor and DATA is either COVIMOD simple $\text{approach}_{\text{relative reduction}}$, COVIMOD complex $\text{approach}_{\text{relative reduction}}$, $\text{Google}_{\text{relative reduction}}$ or $\text{Apple}_{\text{relative reduction}}$.

The relative reduction including the scaling is therefore:

$$DATA_{scaled} = DATA * \beta$$

where DATA is either COVIMOD simple $\text{approach}_{\text{relative reduction}}$, COVIMOD complex $\text{approach}_{\text{relative reduction}}$, $\text{Google}_{\text{relative reduction}}$ or $\text{Apple}_{\text{relative reduction}}$ and β is the scaling factor.

STEP 2

To compute confidence intervals, we run the *optim* function inside a bootstrap-routine with 1000 bootstrapped samples, i.e.

$$\sum_{wave=1}^{4} (\delta * DATA\_bootstrapped_{wave} - RKI\_bootstrapped_{relative\ reduction;\ wave})^2$$

Where *_bootstrapped* denotes the bootstrapped COVIMOD simple $\text{approach}_{\text{relative reduction}}$, $\text{Google}_{\text{relative reduction}}$, $\text{Apple}_{\text{relative reduction}}$ or $\text{RKI}_{\text{relative reduction}}$.

For the estimation of the reproduction number based on COVIMOD data (COVIMOD complex approach) already 10,000 bootstrapped samples were drawn, we used those instead, i.e. we calculated the relative reductions for each of the estimated reproduction numbers of the 10,000 samples and multiplied that by the scaling factor for the COVIMOD complex approach in step 1.

4 Relative reduction in transmission dynamics with fitted weights for home/non-home contacts/mobility

The below analysis produced Figure 4c in the manuscript

NOTE: Apple mobility data could not be used for these analyses as there is no differentiation in home/non-home mobility available

The following estimates obtained under (2) are used as a base: $RKI_{relative\ reduction}$, COVIMOD simple $approach_{relative\ reduction}$, COVIMOD complex $approach_{relative\ reduction}$, $Google_{relative\ reduction}$.

STEP 1 – weighted means home/non-home contacts

We generated the weighted relative reduction in population mobility for Google separately for home and non-home mobility, $\bar{X}_{Google\_home}$ and $\bar{X}_{Google\_non-home}$ and the weighted mean of the reported number of home and non-home contacts during the SARS-CoV-2 pandemic (COVIMOD - simple approach), $\bar{X}_{COVIMOD\ simple\_home}$ and $\bar{X}_{COVIMOD\ simple\_non-home}$, and before the pandemic $\bar{X}_{POLYMOD\_home}$ and $\bar{X}_{POLYMOD\_non-home}$.

From the COVIMOD data, we also estimated the mean reproduction number from the eigenvalue obtained from the symmetric next generation matrix by assuming R0 pre-pandemically to follow a normal distribution with mean 2.6 and standard deviation 0.54 and taking the survey weights into account also separately for home and non-home contacts (COVIMOD – complex approach; $\bar{X}_{COVIMOD\ complex\_home}$ and $\bar{X}_{COVIMOD\ complex\_non-home}$).

STEP 2 – relative reductions in transmission dynamics separately for home and non-home contacts

<u>Population mobility:</u>

As Google mobility data already describe the relative reduction in mobility compared to mobility before the SARS-CoV-2 pandemic in Germany, the weighted means obtained above are describing the relative reduction.

$Google_{relative\ reduction\_home} = \bar{X}_{Google\_home}$

$Google_{relative\ reduction\_non-home} = \bar{X}_{Google\_non-home}$

We then computed a weighing variable (W_{Google}), so that:

$(Google_{relative\ reduction\_home} + W_{Google} * Google_{relative\ reduction\_non-home})/2 = Google_{relative\ reduction}$

COVIMOD simple approach:

We estimated the relative reduction in contacts during the pandemic compared to the pre-pandemic using the weighted mean reported number of contacts from COVIMOD and using the weighted mean reported number of contacts from the POLYMOD data as a baseline.

COVIMOD simple approach$_{\text{relative reduction_home}}$ = $\frac{(\bar{X}_{POLYMOD\_home} - \bar{X}_{COVIMOD\ simple\_home}) * 100}{\bar{X}_{POLYMOD\_}home}$

COVIMOD simple approach$_{\text{relative reduction_non-home}}$ = $\frac{(\bar{X}_{POLYMOD\_non-home} - \bar{X}_{COVIMOD\ simple\_non-home}) * 100}{\bar{X}_{POLYMOD\_}non-home}$

We then computed a weighing variable ($W_{\text{COVIMOD simple}}$), so that:

(COVIMOD simple approach$_{\text{relative reduction_home}}$ + $W_{\text{COVIMOD simple}}$ *COVIMOD simple approach$_{\text{relative reduction_non-home}}$)/2 = COVIMOD simple approach$_{\text{relative reduction}}$

COVIMOD complex approach:

For the complex approach, we used the estimated reproduction number based on the COVIMOD data and calculated the relative reduction compared to the basic reproduction number:

COVIMOD complex approach $_{\text{relative reduction\_home}}$ = $\frac{(2.6 - \bar{X}_{COVIMOD\ complex\_home})}{2.6} * 100$

COVIMOD complex approach $_{\text{relative reduction\_non-home}}$ = $\frac{(2.6 - \bar{X}_{COVIMOD\ complex\_non-home})}{2.6} * 100$

We than computed a weighing variable ($W_{\text{COVIMOD complex}}$), so that:

(COVIMOD complex approach$_{\text{relative reduction_home}}$ + $W_{\text{COVIMOD comples}}$ *COVIMOD complex approach$_{\text{relative reduction_non-home}}$)/2 = COVIMOD complex approach$_{\text{relative reduction}}$

STEP 3

We performed an analysis in which we fitted the relative reductions in contacts/mobility to the reference (RKI estimates) and allowed independent scaling factors for home contacts/mobility and non-home contacts/mobility. For this, we minimised the residual sum of squares across the four survey waves using the *optim* function in R, i.e.:

$$\sum_{wave=1}^{4}((\beta(1) * DATA_{wave_{home}} + \beta(2) * W * DATA_{wave_{non-home}})/2 - RKI_{relative\ reduction;\ wave})^2$$

Where $\beta(1)$ and $\beta(2)$ are the scaling factors and $DATA_{\text{home}}$/$DATA_{\text{non-home}}$ are either COVIMOD simple approach$_{\text{relative reduction_home}}$/COVIMOD simple approach$_{\text{relative reduction_non-home}}$, COVIMOD complex

approach$_{relative\ reduction_home}$/COVIMOD complex approach$_{relative\ reduction_non-home}$, Google$_{relative\ reduction_home}$/Google$_{relative\ reduction_non-home}$ and W is W_{Google} or $W_{COVIMOD\ simple}$ or $W_{COVIMOD\ complex}$.

The relative reduction including the scaling for home and non-home contacts/mobility is, therefore:

$$DATA_{scaled} = (DATA_{home} * \beta(1) + DATA_{non-home} * W * \beta(2))/2$$

Where $\beta(1)$ and $\beta(2)$ are the scaling factors and DATA$_{home}$/DATA$_{non-home}$ are either COVIMOD simple approach$_{relative\ reduction_home}$/COVIMOD simple approach$_{relative\ reduction_non-home}$, COVIMOD complex approach$_{relative\ reduction_home}$/COVIMOD complex approach$_{relative\ reduction_non-home}$, Google$_{relative\ reduction_home}$/Google$_{relative\ reduction_non-home}$ and W is W_{Google} or $W_{COVIMOD\ simple}$ or $W_{COVIMOD\ complex}$.

STEP 4

To compute confidence intervals, we run the *optim* function inside a bootstrap-routine with 1000 bootstrapped samples, i.e.

$$\sum_{wave=1}^{4}((\beta(1) * DATA\_bootstrapped_{wave_{home}} + \beta(2) * W * DATA\_bootstrapped_{wave_{non-home}})/2 - RKI\_bootstrapped_{\ relative\ reduction;\ wave})^2$$

Where *_bootstrapped* denotes the bootstrapped COVIMOD simple approach$_{relative\ reduction_home}$/ COVIMOD simple approach$_{relative\ reduction_non-home}$, Google$_{relative\ reduction_home}$/ Google$_{relative\ reduction_non-home}$, or RKI$_{relative\ reduction}$.

For the estimation of the reproduction number based on COVIMOD data (COVIMOD complex approach) already 10,000 bootstrapped samples were drawn, we used those instead, i.e. we calculated the relative reductions for each of the estimated reproduction numbers of the 10,000 samples and multiplied those by the scaling factors for the COVIMOD complex approach in step 3.

5 Relative reduction in transmission dynamics with normalised weights for home/non-home contacts/mobility as well as allowing a scaling factor.

We used normalised weights based on setting-specific secondary attack rates (SAR) based on a meta-analysis by Thompson *et al* [9]. The household SAR was estimated to be 21.1 (home), and the average of healthcare, workplace and casual close contacts was estimated to be 2.23 (non-home).

Therefore:

weight_home = 21.1/(21.1+2.23)

weight_non_home = 2.23/(21.1+2.23)

The below analysis produced Figure 4d in the manuscript

NOTE: Apple mobility data could not be used for these analyses as there is no differentiation in home/non-home mobility available

The following estimates obtained under (2) are used as a base: $\text{RKI}_{\text{relative reduction}}$, $\text{COVIMOD simple approach}_{\text{relative reduction}}$, $\text{COVIMOD complex approach}_{\text{relative reduction}}$, $\text{Google}_{\text{relative reduction}}$.

STEP 1 – weighted means home/non-home contacts

We generated the weighted relative reduction in population mobility for Google separately for home and non-home mobility, $\bar{X}_{Google\_home}$ and $\bar{X}_{Google\_non-home}$ and the weighted mean of the reported number of home and non-home contacts during the SARS-CoV-2 pandemic (COVIMOD - simple approach), $\bar{X}_{COVIMOD\ simple\_home}$ and $\bar{X}_{COVIMOD\ simple\_non-home}$, and before the pandemic $\bar{X}_{POLYMOD\_home}$ and $\bar{X}_{POLYMOD\_non-home}$.

From the COVIMOD data, we also estimated the mean reproduction number from the eigenvalue obtained from the symmetric next generation matrix by assuming R0 pre-pandemically to follow a normal distribution with mean 2.6 and standard deviation 0.54 and taking the survey weights into account also separately for home and non-home contacts (COVIMOD – complex approach; $\bar{X}_{COVIMOD\ complex\_home}$ and $\bar{X}_{COVIMOD\ complex\_non-home}$).

STEP 2 – relative reductions in transmission dynamics separately for home and non-home contacts

<u>Population mobility:</u>

As Google mobility data already describe the relative reduction in mobility compared to mobility before the SARS-CoV-2 pandemic in Germany, the weighted means obtained above describe the relative reduction.

$\text{Google}_{\text{relative reduction\_home}} = \bar{X}_{Google\_home}$

Google$_{\text{relative reduction_non-home}}$ = $\bar{X}_{Google\_non-home}$

We then computed a weighing variable (W_{Google}), so that:

(Google$_{\text{relative reduction_home}}$ + W_{Google}*Google$_{\text{relative reduction_non-home}}$)/2 = Google$_{\text{relative reduction}}$

COVIMOD simple approach:

We estimated the relative reduction in contacts during the pandemic compared to the pre-pandemic using the weighted mean reported number of contacts from COVIMOD and using the weighted mean reported number of contacts from the POLYMOD data as a baseline.

COVIMOD simple approach$_{\text{relative reduction_home}}$ = $\frac{(\bar{X}_{POLYMOD\_home} - \bar{X}_{COVIMOD\ simple\_home}) * 100}{\bar{X}_{POLYMOD\_home}}$

COVIMOD simple approach$_{\text{relative reduction_non-home}}$ = $\frac{(\bar{X}_{POLYMOD\_non-home} - \bar{X}_{COVIMOD\ simple\_non-home}) * 100}{\bar{X}_{POLYMOD\_non-home}}$

We then computed a weighing variable ($W_{\text{COVIMOD simple}}$), so that:

(COVIMOD simple approach$_{\text{relative reduction_home}}$ + $W_{\text{COVIMOD simple}}$ *COVIMOD simple approach$_{\text{relative reduction_non-home}}$)/2 = COVIMOD simple approach$_{\text{relative reduction}}$

COVIMOD complex approach:

For the complex approach, we used the estimated reproduction number based on the COVIMOD data and calculated the relative reduction compared to the basic reproduction number:

COVIMOD complex approach $_{\text{relative reduction\_home}}$ = $\frac{(2.6 - \bar{X}_{COVIMOD\ complex\_home})}{2.6} * 100$

COVIMOD complex approach $_{\text{relative reduction\_non-home}}$ = $\frac{(2.6 - \bar{X}_{COVIMOD\ complex\_non-home})}{2.6} * 100$

We then computed a weighing variable ($W_{\text{COVIMOD complex}}$), so that:

(COVIMOD complex approach$_{\text{relative reduction_home}}$ + $W_{\text{COVIMOD comples}}$ *COVIMOD complex approach$_{\text{relative reduction_non-home}}$)/2 = COVIMOD complex approach$_{\text{relative reduction}}$

STEP 3

In addition to the normalised weights, we allowed for an additional scaling factor per contact survey approach, i.e. COVIMOD simple approach, COVIMOD complex approach and Google mobility data.

For this, we minimised the residual sum of squares across the four survey waves using the *optim* function in R, i.e.:

$$\sum_{wave=1}^{4}((\alpha * weight\_home * DATA_{wave_{home}} + weight\_non\_home * \alpha * W * DATA_{wave_{non-home}})/2 - RKI_{relative\ reduction;\ wave})^2$$

Where α is the scaling factor and $DATA_{home}$/$DATA_{non\text{-}home}$ are either COVIMOD simple approach$_{relative\ reduction_home}$/COVIMOD simple approach$_{relative\ reduction_non\text{-}home}$, COVIMOD complex approach$_{relative\ reduction_home}$/COVIMOD complex approach$_{relative\ reduction_non\text{-}home}$, Google$_{relative\ reduction_home}$/Google$_{relative\ reduction_non\text{-}home}$ and W is W_{Google} or $W_{COVIMOD\ simple}$ or $W_{COVIMOD\ complex}$.

The relative reduction including the scaling for home and non-home contacts/mobility is, therefore:

$$DATA_{scaled} = (weight\_home * DATA_{home} * \alpha + weight\_non\_home * DATA_{non-home} * W * \alpha)/2$$

Where α is the scaling factor and $DATA_{home}$/$DATA_{non\text{-}home}$ are either COVIMOD simple approach $_{relative\ reduction\_home}$/COVIMOD simple approach$_{relative\ reduction_non\text{-}home}$, COVIMOD complex approach $_{relative\ reduction\_home}$/COVIMOD complex approach $_{relative\ reduction\_non\text{-}home}$, Google $_{relative\ reduction\_home}$/Google $_{relative\ reduction\_home}$ and W is W_{Google} or $W_{COVIMOD\ simple}$ or $W_{COVIMOD\ complex}$.

STEP 4

To compute confidence intervals, we run the *optim* function inside a bootstrap-routine with 1000 bootstrapped samples, i.e.

$$\sum_{wave=1}^{4}((weight\_home * \alpha * DATA_{bootstrapped_{wave_{home}}} + weight\_non\_home * \alpha * W * DATA\_bootstrapped_{wave_{non-home}})/2 - RKI\_bootstrapped_{\ relative\ reduction;\ wave})^2$$

Where *_bootstrapped* denotes the bootstrapped COVIMOD simple approach$_{relative\ reduction_home}$/ COVIMOD simple approach$_{relative\ reduction_non\text{-}home}$, Google $_{relative\ reduction\_home}$/ Google $_{relative\ reduction\_non\text{-}home}$, or RKI $_{relative\ reduction}$.

For the estimation of the reproduction number based on COVIMOD data (COVIMOD complex approach) already 10,000 bootstrapped samples were drawn, we used those instead, i.e. we calculated the relative reductions for each of the estimated reproduction numbers of the 10,000 samples and multiplied those by the scaling factors for the COVIMOD complex approach in step 3.

6 Repeated measures ANOVA

Repeated measures ANOVA was used to assess differences between error rates provided by the different data sources (COVIMOD, Google, Apple). The repeated measures ANOVA generated an F-statistic used in determining the statistical significance. The F statistic is represented as

$$F = \frac{MS_{data}}{MS_{error}} = \frac{\frac{SS_{data}}{k-1}}{\frac{SS_{error}}{(n-1)(k-1)}}$$

$$SS_{data} = \sum_{i=1}^{k} n_i \, (\bar{x}_i - \bar{x})^2$$

$$SS_{waves} = k * \sum(\bar{y}_i - \bar{x})^2$$

$$SS_{within-group} = \sum\nolimits_1 (x_{i1} - \bar{x}_1)^2 + \sum\nolimits_2 (x_{i2} - \bar{x}_2)^2 + \ldots + \sum\nolimits_k (x_{ik} - \bar{x}_k)^2$$

$$SS_{error} = SS_{within-group} - SS_{waves}$$

where MS_{model} and MS_{error} are the mean sum of squares for data and mean sum of squares for error between related data sources.

k is the number of data category (e.g., Google and Apple we are considering), n_i is the number of waves (wave 1, wave 2, wave 3, wave 4) under each (i^{th}) data category. $\bar{x}_i$ is the mean score for each (i^{th}) data category, $\bar{x}$ is the grand mean and $\bar{y}_i$ is the mean of waves i.

The R *aov* function was used for this analysis.

R version 4.0.2 was used for all analyses [10]

References

1. Robert Koch Institut. Schätzung der aktuellen Entwicklung der SARS-CoV-2-Epidemie in Deutschland - Nowcasting. https://www.rki.de/DE/Content/InfAZ/N/Neuartiges_Coronavirus/Projekte_RKI/Nowcasting.html. Accessed 18 Mar 2021.

2. an der Heiden M, Hamouda O. Schätzung der aktuellen Entwicklung der SARS-CoV-2- Epidemie in Deutschland – Nowcasting. Epidemiol Bull. 2020;17:10–5.

3. Robert Koch Institut. Erläuterung der Schätzung der zeitlich variierenden Reproduktionszahl R. 2020. https://www.rki.de/DE/Content/InfAZ/N/Neuartiges_Coronavirus/Projekte_RKI/R-Wert-Erlaeuterung.html. Accessed 6 Oct 2020.

4. Höhle M, An Der Heiden M. Bayesian nowcasting during the STEC O104: H4 outbreak in Germany, 2011. Biometrics. 2014;70:993–1002.

5. Jarvis CI, Van Zandvoort K, Gimma A, Prem K, Auzenbergs M, O'Reilly K, et al. Quantifying the impact of physical distance measures on the transmission of COVID-19 in the UK. BMC Med. 2020;18:124.

6. Google. Mobilitätsberichte zur Coronakrise. https://www.google.com/covid19/mobility/. Accessed 20 Aug 2020.

7. Apple. COVID-19 – Berichte zu Mobilitätstrends. 2020. https://covid19.apple.com/mobility. Accessed 20 Aug 2020.

8. Canty A, Ripley BD. boot: Bootstrap R (S-Plus) Functions. 2021.

9. Thompson HA, Mousa A, Dighe A, Fu H, Arnedo-Pena A, Barrett P, et al. Severe Acute Respiratory Syndrome Coronavirus 2 (SARS-CoV-2) Setting-specific Transmission Rates: A Systematic Review and Meta-analysis. Clin Infect Dis. 2021.

10. R Core Team. R: A Language and Environment for Statistical Computing. 2020.
